# Lessons From the COVID-19 Pandemic to Improve the Health, Social Care, and Well-being of Minoritized Ethnic Groups With Chronic Conditions or Impairments: Protocol for a Mixed Methods Study

**DOI:** 10.2196/38361

**Published:** 2022-07-01

**Authors:** Carol Rivas, Kusha Anand, Alison Fang-Wei Wu, Louise Goff, Ruth Dobson, Jessica Eccles, Elizabeth Ball, Sarabajaya Kumar, Jenny Camaradou, Victoria Redclift, Bilal Nasim, Ozan Aksoy

**Affiliations:** 1 Social Research Institute University College London London United Kingdom; 2 Department of Nutritional Sciences King’s College London London United Kingdom; 3 Wolfson Institute of Population Health Queen Mary University of London London United Kingdom; 4 Department of Neuroscience Brighton and Sussex Medical School Brighton United Kingdom; 5 School of Health Sciences City University London London United Kingdom; 6 Department of Obstetrics and Gynaecology, The Royal London Hospital Barts Health National Health Service Trust London United Kingdom; 7 Department of Political Science University College London London United Kingdom

**Keywords:** racism, minoritized ethnic group, disabled, social care, intersectional, pandemic, social networks, public health, migrant, COVID-19

## Abstract

**Background:**

The COVID-19 pandemic has inequitably impacted the experiences of people living with ill health/impairments or from minoritized ethnic groups across all areas of life. Given possible parallels in inequities for disabled people and people from minoritized ethnic backgrounds, their existence before the pandemic and increase since, and the discriminations that each group faces, our interest is in understanding the interplay between being disabled AND being from a minoritized ethnic group.

**Objective:**

The overarching aim of the Coronavirus Chronic Conditions and Disabilities Awareness (CICADA) project, building on this understanding, is to improve pandemic and longer-term support networks, and access to and experiences of care, services, and resources for these underserved groups, both during the pandemic and longer term, thereby reducing inequities and enhancing social, health, and well-being outcomes.

**Methods:**

This mixed methods study involves three “sweeps” of a new UK survey; secondary analyses of existing cohort and panel surveys; a rapid scoping review; a more granular review; and qualitative insights from over 200 semistructured interviews, including social network/map/photo elicitation methods and two subsequent sets of remote participatory research workshops. Separate stakeholder cocreation meetings, running throughout the study, will develop analyses and outputs. Our longitudinal study design enables the exploration of significant relationships between variables in the survey data collected and to the assessment of changes in variables over time, including consideration of varying pandemic contexts. The qualitative data will provide more granular detail. We will take a strengths and assets–based approach, underpinned by the social model of disability and by intersectional considerations to challenge discrimination. Our exploration of the social determinants of health and well-being is framed by the social ecological model.

**Results:**

The CICADA project was funded by the Health and Social Care Delivery Research (HSDR) Programme of the United Kingdom (UK) National Institute for Health and Care Research (NIHR) in March 2021 and began in May 2021. Further work within the project (84 interviews) was commissioned in March 2022, a substudy focusing on mental health, specifically in Northeast England, Greater Manchester, and the Northwest Coast of the United Kingdom. Data collection began in August 2021, with the last participants due to be recruited in September 2022. As of January 2022, 5792 survey respondents and 227 interviewees had provided data. From April 2022, the time of article submission, we will recruit participants for the substudy and wave 2 of the surveys and qualitative work. We expect results to be published by winter 2022.

**Conclusions:**

In studying the experiences of disabled people with impairments and those living with chronic conditions who come from certain minoritized ethnic groups, we are aiming for transformative research to improve their health and well-being.

**International Registered Report Identifier (IRRID):**

DERR1-10.2196/38361

## Introduction

### Background and Rationale

The particular challenges faced by vulnerable groups during the COVID-19 pandemic, including people from minoritized ethnic backgrounds and people with underlying health conditions/impairments [1-7], are now well recognized. In the United Kingdom, 17.2% of the population were recorded as disabled in 2020, but represented 59.5% of all UK COVID-19 deaths up to November 2020 [8]. Similarly, although 13% of the UK population are from minoritized ethnic backgrounds, they represented 33% of critically ill COVID-19 patients between February and August 2020 [5,6]. This was partly because chronic conditions such as diabetes and cardiovascular disease are disproportionately prevalent in some minoritized ethnic groups [9] as well as being risk factors for serious illness or death from COVID-19 [10]. Moreover, perceived discrimination and perceived lower socioeconomic status are also associated with a greater COVID-19 health risk [11]. These findings show the importance of considering different intersecting factors that compromise good health outcomes. As shown in Textbox 1, the pandemic has inequitably impacted the experiences of people living with ill health/impairments or from minoritized ethnic groups across all areas of life and not only in relation to COVID-19 illness [1,2,11].

**Inequities for minoritized ethnic groups, and those with chronic conditions/impairments, increasing their risk of poor pandemic health and well-being outcomes.**

**1. Increased risk of isolation, abuse, or neglect**, **and poor access to informal emotional and well-being support** due to national pandemic responses, stigma, changed activities, priorities, attitudes of others, a state of “normalized absence, pathologized presence,” among other factors [12].

**2. Inequitable formal treatment, support, and care** from attitudinal, structural, policy, cultural, linguistic, communication, and economic barriers, leading for example to difficulties implementing recommended COVID-19 avoidance strategies, vaccine mistrust, and risk of severe illness.

**3. Psychosocial factors raising COVID-19 risks, reducing the capacity to cope** with social, economic, and psychological pandemic impacts, including worries about people “back home.”

**4. Unemployment/reduced income** (eg, zero-hour contracts, overrepresentation in the unskilled service sector, “no recourse” to welfare).

Given parallels in the inequities for disabled people and people from minoritized ethnic backgrounds, the increase in these inequities since the pandemic, and the discrimination that each group faces, we were interested in understanding the interplay between being disabled AND being from a minoritized ethnic group. This has been a neglected area in research and policy. Certainly before COVID-19 vaccination programs were rolled out, there was more focus on the COVID-19 mortality rates of discriminated-against groups than on their general health and well-being during the pandemic. Moreover, international concern about pandemic-induced mental health issues has tended to take a population-wide focus, sidelining the especially poor pandemic-related mental health experienced by some people from different minoritized ethnic groups [13] (for an example, see [14]).

Most peer-reviewed published articles on chronic conditions/impairments and the pandemic have been survey- or audit-based considerations of reduced patient footfall for, or access to, consultations. In a global COVID-19 survey, 17% of 548 respondent rheumatologists estimated that 25% of their patients had no access to telehealth [15] and therefore little clinical support. It is increasingly recognized worldwide that the rapid move to remote health care has accentuated inequities for some. Problems with pandemic telehealth services are currently under scrutiny in the United Kingdom and have been experienced by Coronavirus Chronic Conditions and Disabilities Awareness (CICADA) study clinical team members [16], although remote consultations also have recognized benefits.

Interviews in Italy with representatives from seven voluntary organizations that specialized in disability highlighted bureaucratic challenges, and shortfalls in advice, coordinated care plans, and interagency coordination to compensate for reduced services in the pandemic [17]. Similar issues have been reported in the grey literature. Systemic prepandemic failures were perceived by respondents to a European Federation of Neurological Associations global survey to have led to the collapse of normal neurology care pathways during the pandemic [18]. Health care services for people with rare and complex conditions have fared especially badly according to the European H-CARE Survey [19]. In the United Kingdom, the organization National Voices collated 2020 data from a range of third-sector pandemic surveys specializing in disability and health conditions, reporting issues with mental health; managing symptoms and/or deteriorating health and finances; access to medication, food, health, and social care; impacts on carers; and problems with accessing or understanding information [20].

There are several examples in peer-reviewed journals of small surveys internationally that have shown how reduced access to treatment negatively impacts patients’ symptomatic control, including cases of Parkinson disease [21], migraine [22], rheumatology [23], and chronic refractory neuropathic pain [24], leading to an increased reliance on support networks [25].

Even within these studies, there is very little recognition of the way the particular challenges faced as a result of belonging to a minoritized ethnic group might intersect with, or be compounded by, the challenges faced by having underlying health conditions/impairments. Minoritized ethnic groups with a chronic condition or impairment are more likely to die from COVID-19 [3-8,10,25] in the historical context of poorer health outcomes more generally [4,7,26-28]. The unifying explanation is ingrained racism. Twenty-five percent of doctors responding in a US survey reported that preexisting socioeconomic issues caused by structural racism, combined with institutional racism, when added to pandemic constraints on care, made it even more challenging to care for Black patients with asthma than others in the pandemic [29]. Another US survey showed that pandemic telehealth was used by Black patients more than by White patients. This was attributed to the need of Black patients to compensate for prior health and health care disparities caused by systemic racism [30].

### Aim and Research Questions

Given the current evidence gaps and the pressing need for these to be filled, the broad questions underlying this research project are therefore: (1) Are the pandemic-related issues faced in different aspects of daily living summative, additive, or broadly similar in people from minoritized ethnic groups who also have chronic conditions/impairments as compared to people belonging to either one of these two categories? (2) What can we learn about how different people successfully draw on different assets, coping strategies, and other strengths or developed solutions to deal with these issues in different pandemic contexts? (3) Which intersecting social categories are the most significant in shaping these answers? (4) How can a systematic, living map of existing evidence contribute to understanding the pandemic-relevant experiences of having an impairment/chronic condition and belonging to a minoritized ethnic group?

In aiming to answer these questions, we will undertake primary and secondary research to improve our understanding of the pandemic-related issues faced by minoritized ethnic groups with chronic conditions/impairments in different aspects of daily living, and the different assets, strengths, and solutions they have drawn on. To better understand their particular experiences, we compare their perspectives with those of people self-identifying as being of White British ancestry, with and without chronic conditions/impairments, and people from minoritized ethnic groups with no chronic conditions/impairments. We will use our findings to help to mitigate inequities, and improve their experiences; support networks; and access to and experiences of care, services, and resources. We plan to achieve this by developing and informing evidence-based formal and informal strategies, guidelines, recommendations, and interventions for health and social care policy and practice. These outputs are intended to improve social, health, and well-being outcomes for underserved groups, both during the COVID-19 pandemic and in the longer term.

### Theoretical Underpinnings

#### Theoretical Framework

**Figure 1:**
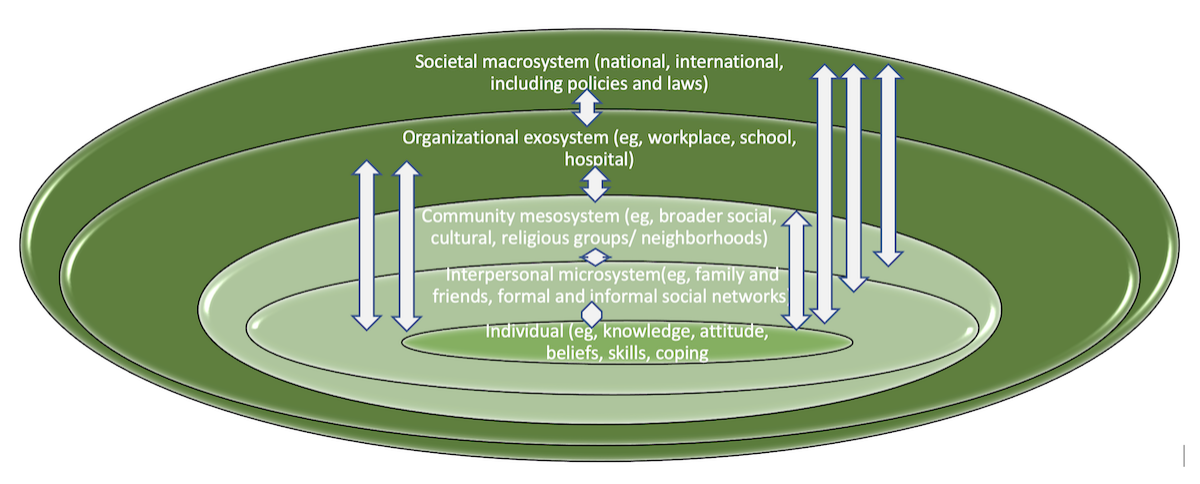
The social ecological model (adapted from Bronfenbrenner [35]); arrows show the bidirectional flows of interactions between levels as a complex system.

We will take a strengths and assets–based approach, underpinned by the social model of disability and by intersectional considerations to challenge discrimination [31,32]. Our exploration of the social determinants of health and well-being is framed by the social ecological model [33,34] (Figure 1).

#### Disability Models

Medical or biopsychosocial models of disability have led to the continued disenfranchisement and marginalization of people with physiological impairments, through the conflation of pathoanatomical diagnostic criteria with disability itself [35,36]. In other words, the two are inseparable and the person with the diagnosis is only seen as dysfunctional. This leads to ableism (discrimination in favor of nondisabled people) [37] and disablism, defined as “discriminatory, oppressive, or abusive behaviors arising from the belief that disabled people are inferior to others” [38] (page 9).

The CICADA study resists the use of these deficit-focused disability models, instead taking as its starting point the social model of disability because of its currency, usefulness in driving transformative outputs, and relevance to much-needed revisions in continuing discriminatory statute and law [39]. This continued discrimination persists despite the United Nations Convention on the Rights of Persons with Disabilities [40]. In the social model, impairments, as physiological problems of the body, are decoupled from disability, which results from exclusionary social oppression and prejudices [37]. Society, by accommodating impairments through the removal of iniquitous barriers to inclusion, can enable the full participation of all, across every area of life.

While important, the social model has limitations that are the topic of much discussion [41,42]. For example, Siebers [41] argues for: (1) more complex understandings of embodied variation and (2) more dynamic problematization of the liminal spaces occupied by lived reality than afforded by the social model. Arendt’s [42] criticism of the social model is that more account needs to be taken of the ways people with impairments internalize and make meaning of their lived experiences. We will therefore also draw on alternative nondeficit models in our analyses. Our overall interest is in how individuals with chronic conditions or impairments experience and make meaning of the world through their embodiment within it at the intersection with various other simultaneously and variably interacting social factors.

#### Intersectionality

Rather than separately considering the multiple social categories of “identity, difference, and disadvantage” [43] (p.171) such as gender, racial/ethnic minoritization, disability, and occupation, we consider them as coexisting interacting systems of oppression. In other words, they work together (are mutually constitutive) under discriminatory institutional and structural conditions to create [44-47] lower levels of physical and mental health, poor access to quality health care, and poorer health outcomes [48-51]. Citizenship status adds an infrequently considered further important layer of complexity that we explore in the CICADA study [50]. There is a particular lack of research on the ways that health outcomes are shaped for undocumented migrants through their structural construction as “illegal” [51] within a hostile environment [52] with “no recourse” to welfare and housing support.

We consider both individual experiences of day-to-day discrimination and the wider context. We use the term “minoritized ethnic groups” to emphasize the stigmatization and oppression that a racialized society bestows on particular ethnic groups as racialized “others” [53], rather than to necessarily ascribe to them a “minority” status. An alternative term, “racialized communities,” is also used in some study documents to indicate the nature of this oppression.

Shifting identities among people who have recently migrated, in the face of racism, can include the racialization process of “becoming White.” This process tends to be neglected in the health literature [54] and is one that we also consider. We hypothesize negative consequences for recent immigrants of Arab or Central and East European ancestry, who may experience the tensions of being symbolically included in a White ethnic category but are excluded from many of its benefits [54], in a manner that is often invisible because of the lack of its exploration.

Through considerations such as these, built into the study design, intersectionality theory will allow us to develop complex nuanced insights into differences, while minimizing the risks of essentializing some combinations as inherently problematic or considering the minoritized experience as homogenous.

#### Social Ecological Approach

Intersectionality conceptualizes the ways an individual’s social interactions are shaped by their multiple subject positions (eg, as a female, recent migrant, disabled person). The social ecological model [33,34] highlights the ways this individual is positioned at the center of a system of mutually influencing sets of social determinants, incorporating their personal, community, regional, and national (policy and society) ecosystems of norms and practices. Embodied experiences of migration, citizenship, chronic conditions, and impairments are necessarily intersectional with areas of potential discrimination and oppression across the different levels of the social ecological model. Hence, there is a need for a range of comparisons and involvement of multiple stakeholders in our study to ensure that any potential strategies and recommendations we develop will apply both within and across the different levels [55]. This also fits with the new UK National Health Service (NHS) tiered Integrated Care Plan [56], which is relevant as the CICADA study is set in the United Kingdom.

Intersectional interactions across the levels of the social ecological model are in constant flux, which Bronfenbrenner [57] represented by the chronosystem in development of his original model. Recognizing the importance of these changes over time, our study is longitudinal. Our work is also underpinned by the Consolidated Framework for Implementation Research (CFIR) [58]. This is an amalgamation of various implementation theories that target different levels of the social ecological model, and we use it to comprehensively explore the feasibility of implementation of our recommendations and other outputs. The CFIR is easy to operationalize, flexible (the user selects relevant themes from a pool of 39), and facilitates actionable findings across multilevel implementation contexts.

### Assets-Based Approach

Our intersectional and critical disabilities approaches facilitate the interrogation of our data for participant assets and strengths as well as the barriers they face. For example, small cross-sectional analyses suggest that some chronic conditions and impairments may confer resilience to mental health or well-being effects of the pandemic [59,60]. In a UK analysis of chronic fatigue during the pandemic, Reddit reported more severe symptoms in some people but also more accessible opportunities to interact (through online video calls) [61]. Strengths/assets-based approaches involve a holistic focus on both personal strengths (internal factors such as resilience and external factors such as material assets) and social and community networks. This opens up spaces for individuals who experience disadvantage to be viewed as important partners in the development of change processes rather than problems to be acted upon. Our approach falls under a branch of assets-based work sometimes termed “positive deviance.” This looks for positive outcomes in the face of adversity, as well as behavior and community development needs, where further support could develop or add to assets and strengths. We are mindful to ensure this does not reduce the need for state intervention (we will take pains not to deproblematize contexts or suggest that improvements should be a community, rather than a policy, responsibility). A strengths-based approach does not try to take the focus away from the structural causes of inequities [62] but rather aims to empower communities and individuals [62] in meaningful and sustainable ways. It is based on salutogenic theory [63], which positions people as coproducers of health, rather than consumers of health services [64], and recognizes the need to consider that individuals have intersectional identities. This approach has greater transformative potential than deficit-focused approaches [65].

## Methods

### Ethics Approval

The study has Institute of Education, University College London, Research Ethics Approval (UCL IoE REC 1372, and amendment 1450 Covid-19; Data protection registration number: Z6364106/2020/06/24) and will follow FAIR Open Science principles of accountability and transparency [66]. We also have NHS ethics approval to recruit participants at NHS sites (IRAS: 310741, CPMS ID: 51755–CICADA recruitment).

### Overall Design

We will use a longitudinal mixed methods approach to develop a rich understanding of study participants’ mental and physical health, coping strategies, access to resources, and informal and formal social and health care support experiences. We will explore relevant assets and strengths for well-being enhancement, and examine variations through the lens of intersectionality. Analyses, outputs, dissemination, and implementation plans will be cocreated with key stakeholders.

**Figure 2:**
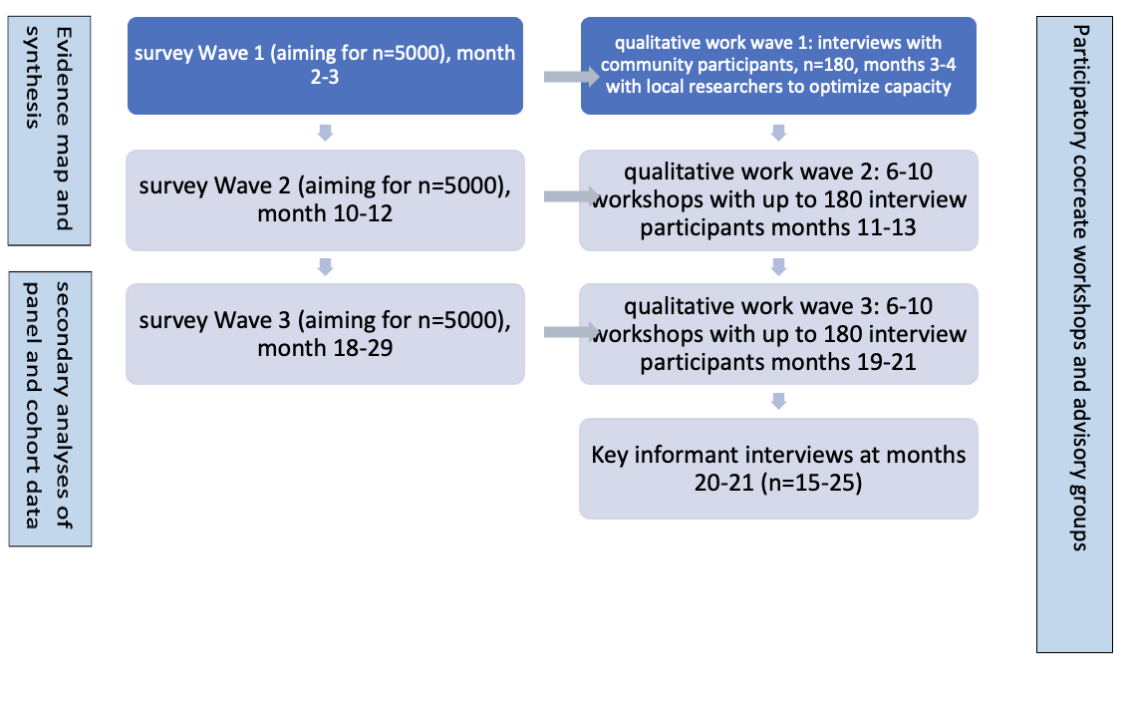
Flowchart for the design of the CICADA study.

Our design (Figure 2) includes three “sweeps” (ie, repetitions) of a new UK survey, secondary analyses of existing cohort and panel surveys, a rapid scoping review, and a more granular review. We will incorporate qualitative insights from 210 semistructured interviews, including network/map/photo elicitation methods, and two subsequent sets of remote participatory research workshops that roughly coincide with survey sweeps two and three, designed instead of second and third sweep interviews to minimize participant burden. Stakeholder cocreation meetings will run throughout the study and are key to implementation of outputs. Policymakers such as those within Public Health England (now the UK Health Security Agency), and practitioners such as clinicians and social support workers specify an urgent need for participatory work with minoritized ethnic groups [67]. Our embedded social network analysis will provide important insights on how to improve information channels, routes into health/social care and support, resilience to stress, and postdisaster recovery [13,65]. We will include consideration of new service delivery models, some of which are already planned to continue beyond the pandemic (eg, telemedicine [68,69]). Our longitudinal study design enables us to explore significant relationships between variables in the survey data we collect and the changes in these variables over time. We will include consideration of varying pandemic contexts such as lockdowns, restrictions, and their relaxation. The qualitative data will provide more granular detail. 

### Primary Data Collection

#### Topic Guides for the Survey, Interviews, and Research Workshops

The lefthand column of Table 1 shows the eight topics that run throughout the study. These topics have informed the reviews, topic guides, research workshops, and surveys. Instruments included within our surveys are shown in the righthand column of Table 1 as an example; they are mostly validated by their developers in different newly migrated populations, and thus are particularly appropriate for use in this study.

**Table 1:** Eight topics that run through all parts of the study, with the corresponding survey instruments/items.

Topics across all study stages that structure the reviews, topic guides, and surveys	Survey instruments/items
Intersectionalities	A range of demographic variables [70,71]
Behavioral responses to COVID-19 risk-reduction measures, including vaccination	“Control of life” (including COVID-19–related aspects)
Access to resources, support, care, vaccines, including digital transformation, service innovations	QOCS-ID^a^ [72], Vulnerability Assessment Framework [73] for care needs, UK government SAGE group–recommended questions
Social networks	Developed from the Close Persons questionnaire [74] to contextualize other topics
Mental and physical well-being/quality of life as primary outcomes	WHOQOL-BREF-ID^b^ [72].
Coping	Including tolerance to uncertainty, positive appraisal style, attitudes to being ill (WHO^c^ ADS^d^ [72]), pandemic health and mental health consequences (Global Mental Health Assessment Tool [75]), why they arose, and how issues can be mitigated
Local and regional differences in responses linked to policies/ interventions and associated impacts	Apart from within-survey analysis, we will match respondents’ area code of postcode with area-level (1) ONS^e^-registered COVID-19 cases, hospitalizations, and deaths; and (2) Google data on social distancing adherence
Vaccines, future policies	Free-text comment boxes

^a^QOCS-ID: World Health Organization Quality of Life-Disability.

^b^WHOQOL-BREF-ID: World Health Organization Quality of Life.

^c^WHO: World Health Organization.

^d^ADS: Anxiety and Depression Scale.

^e^ONS: Office for National Statistics.

#### Primary Outcomes

The primary online survey outcomes are: (1) formal/informal care measured using de novo questions and the World Health Organization (WHO) Quality of Care and Support-Disability questionnaire (QOCS-ID) at 4, 10, and 16 months; (2) quality of life measured using the World Health Organization Quality of Life tool (WHOQOL-BREF-ID) at 4, 10, and 16 months; (3) control of life measured using the “control of life” validated questionnaire at 4, 10, and 16 months; (4) physical and mental health measured using the WHO Anxiety and Depression Scale (ADS) at 4, 10, and 16 months; the Vulnerability Assessment Framework at 10 and 16 months; and the Global Mental Health Assessment Tool at 10 and 16 months; and (5) social networks measured using an adapted Close Persons questionnaire (for online work) at 4, 10, and 16 months in the online survey and as part of the semistructured interview (using closed questions, open questions, photographs taken by participants to represent their networks without any personal identifying information, and maps drawn of networks).

#### Secondary Outcomes

Secondary outcomes include fear of death measured using the Templer Death Anxiety Scale [76] at 10 and 16 months by online survey. Thematic qualitative data from survey free text, interviews, and workshops will be analyzed, including (1) patient experiences of health and social care, and other forms of formal and informal support during the pandemic, and their perspectives on the impacts on their health; (2) consideration of the impacts of their identity (eg, disabled, from a specific ethnicity, from a low-income background) on these experiences; (3) consideration of their beliefs (health beliefs, COVID-19–related beliefs, vaccination beliefs) and how these affect other themes; and (4) consideration of coping mechanisms, and strategies and assets used in relation to their access to and use of resources, services, and support as this affects their health and well-being.

Surveys will vary by sweep. Key outcome and exposure variables that we expect to change over time will be measured in all three sweeps to study trajectories. Theoretically stable concepts (eg, tolerance to uncertainty, demographic characteristics) will be measured only in one sweep. Key topics may be added to sweeps 2 and 3 that have been identified through our other work.

### Review

#### Aims and Process

Our two-stage review work will: (1) create a systematic (living) map to summarize the pandemic-relevant experiences of living with impairments/chronic conditions and/or being from a minoritized ethnic group across the topics listed in Table 1, and (2) undertake an in-depth analysis and synthesis on specific aspects determined according to the map.

This review will ground the research in current evidence and generate themes that can be incorporated in the primary data collection design.

In both stages, two reviewers will independently screen titles, abstracts, and full texts against inclusion criteria, and extract data. They will compare a subset of this work to check for consistency as quality control, with any disagreements to be resolved by a third reviewer. We will assess risk of bias using Cochrane-recommended checklists, also noting the provenance and publication status of sources. Data extraction will be managed in EPPI-Reviewer software and will reflect the inclusion criteria and the designated aims of the review.

#### Inclusion Criteria

The review inclusion criteria, using a modified SPIDER (Sample, Phenomenon of Interest, Design, Evaluation topics, Research source, Setting) [77] framework are summarized in Textbox 2.

The outcomes and the focus of the granular review will depend on the evidence available, and gaps in the evidence will be highlighted for future study. Reporting will follow PRISMA (Preferred Reporting Items of Systematic Reviews and Meta-analyses) guidelines. The review is registered with Prospero (CRD42021262590).

**Inclusion criteria according to the SPIDER framework.**

***S****ample:* People with any chronic condition/impairment and/or from a minoritized ethnic group within their country of residence (see “Setting” below).

***P****henomenon of****I****nterest:* Lived experience during the pandemic, social networks, and relationships between intersectional variables and health and social care outcomes. Testimony from informal and formal carers may be included where it: (1) directly relates to the topics, and (2) considers the perspective of people with a chronic condition/impairment and/or from a minoritized ethnic group.

***D****esign:* All study designs.

***E****valuation topics:* The topics listed in Table 1.

***R****esearch source:* All sources of research evidence, both peer-reviewed and preprint/grey literature, augmented by data from tweets (given a fast-moving pandemic-responsive field) and websites of relevant public bodies/agencies.

*Setting:* International studies (although the setting of our study is the United Kingdom, it is important to develop a broader knowledge that may be transferable to the United Kingdom, or may provide context, useful models, or lessons to be learned).

Filter restrictions are:

*Date:* Peer-reviewed articles published since 2000, grey literature since January 2019, and other sources since 2020 to balance currency of the data with the identification of a broad view of developing issues.

*Language:* English.

#### Data Analysis

Reporting of the data will depend on the types of included studies (eg, descriptive statistics, narrative synthesis, and diagrams). We will perform subgroup analyses where appropriate.

### Use of Existing Data Sets

We will undertake secondary analysis of data relevant to the topics in Table 1 from several existing data collections for triangulation and complementary insights. This includes the ActEarly City Collaboratory Consortium’s [78] pandemic surveys of families in Bradford and East London, and pandemic surveys within nationally representative cohort studies curated at the Centre for Longitudinal Research (CLS), University College London. None of these data sets has our overall focus but they do include some relevant questions. The overlapping variables between the ActEarly, CLS data, and our own survey will enable us to compare and assess data quality across surveys.

To place our primary survey data within existing and prior national contexts, we will perform our secondary quantitative analyses for three periods: before the pandemic (up to January 1, 2020), prior to relaxation of the winter-spring 2021 lockdown in the United Kingdom (up to May 12, 2021), and thereafter (up to autumn 2022). Should the data enable, we will also subdivide the third period to match the dates of the three sweeps of our primary survey. Since these are secondary analyses, we will be mindful of and discuss relevant biases, and will contextualize the results according to the evolution of the pandemic.

### Three-Sweep Primary Survey

#### Survey Sampling and Recruitment

The primary survey is online and will collect quantitative and qualitative (free text) data. Survey sampling across the four nations of the United Kingdom will be open to any adult living in the United Kingdom, but purposively targeted via selected sites and networks to encompass all conditions/impairments and organizations supporting minoritized ethnic groups, including recently arrived and undocumented migrants. Sampling will not depend on individual patient data such as those that could be obtained via electronic health records to ensure we include people who are self-diagnosed or who perceive themselves to have a different diagnosis to the one held in the electronic record, as well as participants not registered with primary care. We will be mindful of the different biases this may cause, and will collect data on whether or not a diagnosis exists and whether the person agrees with this diagnosis for in-depth understanding.

The three survey sweeps will be evenly spaced over 15 months, with each sweep open for 1 month. Recruitment will predominantly involve distribution of a survey link via social media, and specialist and national networks (such as academic, health service, third sector), as well as mailing lists and large databases of adults interested in health research across the United Kingdom. We recognize that this strategy, being nonrandomized, will be biased such as toward those already interested in research participation or who are active users of third-sector sites and have online access. We will make available print copies for community groups involving participants lacking internet access. We will compare respondent demographics to whole population estimates where possible to explore representativeness (although formal data are limited).

#### Survey Numbers and Power

Our new longitudinal survey will enable a description of the trajectories of key variables and outcomes and the links between them. Free-text data will also be analyzed for patient experience. The survey will not be used to test a particular treatment or focus on a single effect.

In our basic structural equation modeling (SEM), we have six core latent variables (factors) per sweep: (1) quality of life, (2) control of life, (3)access to care, (4) coping mechanisms, (5)mental health, and (6)social networks.

Considering statistical power in SEM [79], the required sample size increases with the number of latent variables, but at a decreasing rate (ie, the required sample size difference between a model with one vs two latent variables is larger than that between a model with three vs two latent variables). The required sample size also decreases strongly as the loadings on latent variables increase (ie, the magnitude of the association between latent and observed variables, where values below 0.9 are generally taken to show a confounding effect). Moreover, the power increases as the number of items (questions) used to measure each latent variable increases.

In terms of our study, each latent variable will be measured by several items (the average number being more than 8). In a worst-case scenario with average loadings of around 0.5 and an item missingness of 20% (as suggested from ActEarly work), a sample size of 800 per subgroup per sweep will yield useful analyses. We have four main subgroups (ie, self-identifying as minoritized ethnicities with a chronic condition/impairment, minoritized ethnicities without a chronic condition/impairment, of White British ancestry with a chronic condition/impairment, and of White British ancestry without a chronic condition/impairment). Using these four comparator groups enables us to fully understand the nature of relationships between different variables and the influence of chronic condition/impairment and minoritized ethnic group categories on each, both separately and combined. Thus, the required sample size is calculated at 800×4=3200, although we aim for 5000 for a more robust sample [79].

#### Survey Analysis

A descriptive statistical summary will be updated with each sweep. More in-depth analysis, using SPSS, R, Stata, or Python, will exploit all three sweeps of the data, with the following research questions:

1. How do outcomes (resource access, formal/informal care, quality of life, control of life, physical and mental health, social networks) and outcome trajectories differ by sample subgroups (minoritized group, condition/impairment, citizenship status) and intersectional combinations? This cuts across all three of our overarching research questions.
2. To what extent can COVID-19 prevalence and pandemic adherence to social distancing at the area level explain differences in outcomes and outcome trajectories across subgroups and in terms of intersectionalities (as a proxy for pandemic contextual factors)? This relates to our third overarching research question.
3. How do the outcomes interrelate within and across survey sweeps, and how does this differ across groups of the sample and in terms of intersectionalities? This cuts across all three of our overarching research questions.

For research questions 1 and 2, we will exploit the longitudinal nature of the data using latent growth modeling (LGM), with multiple group analysis, varying different combinations to consider the effect of intersectionalities on outcomes. Depending on geographical coverage and the numbers recruited, the LGM estimation for research question 2 could be carried out at the within-area level to examine the causal impact of the change in COVID-19 prevalence and pandemic social distancing across the sweeps on each key outcome, under the assumption that this is exogenous. We will examine the plausibility of this assumption and detail possible sources of endogeneity. This will be important for policy given our unique subgroups focus. For example, our data could help clarify why specific groups may find it unfeasible to adhere to recommended behavioral responses. For research question 3, we will estimate a developmental cascade model, including all three data sweeps and key variables, to explore how the key variables are associated with one another, both within survey sweeps and over time. We will fit the LGM models using SEM; this offers useful tools for dealing with missing data due to nonresponse and attrition.

The *social network support module* of our model will consider how respondents are connected to others who provide support to the respondent (eg, through relations such as friendship, kinship, exchanges, activities) [80,81]. Characteristics such as the participant network’s size, composition, and resources available will result in a latent “network capital” variable created through measurement analysis within the SEM as a novel contribution. These network metrics will be used to provide a descriptive presentation of the network(s) and any changes over time.

#### Missing Data

We will require completion of almost every question on every page for participants to proceed so that we can undertake the association analyses required. This means that there should generally be no *missing* items in any measures. However, this requirement may lead to *completion attrition*, with respondents giving up and logging off. We will try to mitigate this possibility in the design, which will allow participants to save responses and return to the survey later, and the survey will be developed and piloted with 30 people from our advisory group and patient advisory group (PAG). There is also the risk of attrition *between* sweep*s*. Participants will be asked to provide an email address upon enrolling online. The RedCap online secure system that we will use will automatically recontact them for sweep 2/3 follow-up questionnaires (with reminders). This automatic process makes for efficient and secure second and third sweep recruitment to reduce the risk of missing respondents. Lotteries appear effective in some online surveys [82] and we are including a £50 (~US $63) Amazon voucher as an incentive given at random.

To handle missing data, and address panel attrition and item nonresponse, we will use modern methods, including full information maximum likelihood; multiple imputation with chained equations that produce unbiased estimates under assumptions of missing at random (ie, missingness dependent on observable data only) and multivariate normality; and pattern mixture models that address missing not at random (ie, missingness dependent on unobserved data), assuming correct model specification [83]. These techniques, under certain assumptions, ameliorate loss of statistical power due to missing data and possible biases due to systematic missingness.

### Interviews

#### Interview Sampling Frame

Our interview sampling frame follows an intersectional approach that allows us to consider and compare assumed homogeneity: (1) across chronic conditions/impairments irrespective of heritage, and (2) across ancestries irrespective of chronic condition/impairment. The aim of this is to tease out intersectional factors and heterogeneity. To achieve this, we will use a purposive quota sampling approach. At analysis, the focus may switch to other commonalities such as shared barriers or enablers in accessing health and social care resources.

Possible attrition between sweeps (up to 20% based on ActEarly experience) may require further recruitment if theme/pattern saturation is not reached, or early saturation may lead us to more theoretical sampling. The main interview inclusion criteria are summarized in Textbox 3.

We recognize the heterogeneity within these groups, and the way these categories are laden with assumptions such as with regard to multimorbidities, the concept of being British, the ancestry of people who are born in particular countries, and the apparent essentialization of specific groups. However, to ensure in-depth data while keeping sample numbers to a feasible level, we decided to use these problematic categorizations as tools to better organize our research so that we can then unpack and critique them [84]. The groups have been chosen to reflect recent migration waves to the United Kingdom (albeit that some people from these groups may have lived in the United Kingdom for decades) and to capture those groups most at risk of hospitalization or death from COVID-19.

The main interview exclusion criteria are: (1) student migrants, as they are likely to have structured educational institution support; and (2) residents of detention centers/closed facilities linked to national migration policies (eg, new asylum-seekers/refugees, displaced or trafficked persons), as these are deemed complex cases with specific considerations.

**Inclusion criteria for the interview.**

- Any condition/impairment, including self-diagnosis, that chronically affects daily activities; the condition should have lasted for at least 12 weeks and have no defined endpoint. These will be categorized in an adaptation of the UK Government Statistical Service harmonized data recommendations as: mental, mobility, stamina/breathing/fatigue (including heart problems), hearing/vision loss, developmental/intellectual, and food-related. These categorizations will then be analyzed.
- People living in the United Kingdom who were born in, or whose parents were born in, Arabic, Central and Eastern European, South Asian, or sub-Saharan African countries, with people of self-defined White British ancestry as comparators.
- Aged 18+ years
- Self-identification of migrant status (with recruitment aiming to cover the range of people whose status is categorized as: undocumented, on temporary visas, with indefinite leave to remain, or British citizens).

#### Interview Sites

We sample from five interview sites within England for maximal sampling diversity in migrant population density, proportion of EU to non-EU migrants, and reasons for migration to enhance transferability, which we will explore against our four-nations survey findings. This is important as we only sample in England for qualitative work due to differences in the devolved nations’ responses to the pandemic and their health and social care systems. Sites for our qualitative research are London, Southeast England, Northeast England, West Midlands, and Yorkshire. While this means that some of our findings may be more relevant to England, we expect the principles to be similar across the four nations, and we will consider this in our reporting and outputs.

#### Recruitment to Interviews

We will recruit interview participants from advertisements/links distributed through a range of platforms and networks, as well as through local lay coresearchers. We will rely on participant self-identification of citizenship status and condition/impairment. Posters, advertisements, and snowball sampling will target those who lack resources or technology to be recruited via online messages; they can contact us by telephone or email. Collaborators will provide recruitment and data collection support through organizations such as Born in Bradford (BiB) in Yorkshire, a long COVID center [85] in Gateshead, the Bromley-by-Bow community center in East London, and migrant charities in London and Canterbury.

#### Interview Process

Respondents can choose to have their interviews by phone, remote video methods, or face to face, depending on extant pandemic restrictions. Our PAG leads will help train lay community members to undertake some interviews locally at our five sites, supported by the core team. Interviews will be recorded. Attention will be given to making the interviews fully accessible and inclusive, and all researchers will be vigilant to the participant needs such as requiring frequent breaks.

All potential participants will be informed about the study in plain English (read to them if needed) and told that interviews will be in English by default. Where a participant feels more comfortable being interviewed in another language, if a researcher fluent in that language is available, this will be arranged. Translated and accessible study documents will be provided if required to ensure that participants are able to give fully informed consent.

We will probe in interviews for the same topics as covered in the survey (Table 1). For social networks, we will discuss the data from the following participant preinterview tasks: (1) a brief questionnaire, data from which we will translate into network “maps” using Network Canvas software; and (2) photographs and sketch-maps of the local area where people live, and the places significant to their health care and social interactions, using their smartphones or disposable cameras that we will provide. These data will also be thematically analyzed. This ethnographic approach facilitates a safe social space to communicate difficult issues and has been used to explore migrant resettlement [86].

### Research Workshops and Cocreation Meetings

#### Design

The two research workshops and four cocreation meetings will all be participatory and designed for participants to work in equitable partnership. They will be led by a core team member and a PAG member. They will aim for outputs relevant to the “real world” that will maintain participant voices and will ensure the research outputs can be implemented. Each session will last approximately 4 hours (2 hours if held remotely).

#### Research Workshop Recruitment, Sampling, and Process

The make-up/number of research workshop groups per sweep (2 and 3) will be determined by considering any typologies (patterns in intersectionalities and outcomes) from sweep 1 data. Participants will be recruited from sweep 1 interviews.

Sweep 2 workshops will discuss scenarios, or structured vignettes, shown as short videos recorded by community members reading scripts. Content will be developed from sweep 1 data into a pandemic-relevant story, with accessible transcripts provided in advance. Discussion will consider changes from previous findings. Sweep 3 workshops will follow a similar pattern with updated vignettes. We will also use participatory scenario planning [87], a policy tool whereby participants are encouraged to explore alternative futures, their impacts, and relevant action plans. To ensure inclusivity, we will work with our PAG group on workshop accessibility and will offer repeat interviews as an alternative.

#### Cocreation Meeting Recruitment, Sampling, and Process

Cocreation meeting participants will include patients and carers (our aim is that they will be representative of our interview participants), as well as key stakeholders in their support and care. They will be recruited via professional or dedicated community networks such as government and policy, welfare, social and health care staff migrant, settlement, and racialization-specific services; third-sector organizations; and community leaders. We will aim for two representatives from each of these groups per workshop, thus with approximately 20 people in each meeting. We will be assisted in this process by our PAG.

The cocreation meetings will involve discussion of findings from each data sweep and their cocreated translation into outputs to feed into the next stages or final study outputs, depending on what is appropriate at the time each workshop is held. To enable inclusion and stimulate discussion and outputs, we will use arts-based and participatory approaches such as miro.com, Collaborative Poetics materials [88], and other cocreation tools [89,90].

### Key Informant Interviews

To explore how outputs can be implemented in policy and practice, 15-25 interviews (up to 5 per site) will be conducted with key informants. These will be identified from earlier phases of the study. These are likely to be drawn from the same categories as our cocreation meetings.

Recruitment plans and topic guides will be informed by our other findings and cocreated in our cocreation meetings, and with members of our advisory groups.

### Analysis of Interviews and Workshops

Deductive framework analysis of the workshop, interview, photo, and key informant data will be used for general dissemination and policy-relevant themes that can be mapped to the survey for added insight. We will also remain open to adding inductive themes throughout the analysis. Data collection and analysis will be concurrent for quick outputs and to test emerging and discordant themes.

Interview data will also undergo Keyword in Context (word frequency–based) analysis to compare specific constructs. We will undertake discourse and narrative analyses on a data subset, produced from participant pairs, matched on features identified as important in earlier analysis. Coding, using NVivo, will be undertaken by the core team, with feedback from the advisory groups and cocreation meetings. We will follow good practice for transparency, quality, and rigor. Anonymized data will be archived for secondary analyses.

### PAG Involvement

We have an active PAG; its members will take part in the cocreation meetings, as well as advising on all stages of the study. They will be supported to be coauthors in any publications we coproduce. We have two PAG coapplicant coleads. We will adhere to the seven principles of patient engagement [91], namely: shared purpose, respect and accessibility, representativeness, roles and responsibilities, capacity and capability for engagement, transparency in communication and documentation, and continuity and sustainability.

### Overall Data Synthesis and Dissemination

We will use cascaded dissemination at each data sweep, tailored to our key audiences, that emphasize practical solutions and implementation. The dissemination plan will be determined with our PAG and through our cocreation meetings.

Overall synthesis will provide an executive overview for easy assimilation by policymakers and practitioners. This will indicate where changes to health/social care policy and practice are likely to be most effective. Synthesis will be results-based; that is, tabulation will be derived from data analyses, with table columns for themes/topics and rows for each distinct set of quantitative and qualitative data. Some data will need to be transformed (quantified or qualitized) for tabulation, such as network graphs. We will interrogate the tabulated data using anchor questions based on the PerSPectif (Perspective, Setting, Phenomenon of interest/problem, Environment, [optional Comparison], Time/timing, Findings) framework [92] (eg, informed by patterns of data convergence/divergence).

An overview of findings and ideas for outputs will be presented to participating communities more widely via collaborator platforms, to give them the opportunity to reflect upon and interrogate researchers’ interpretations and analysis of the data. Findings and ideas for outputs will also be distributed through trusted community channels such as places of worship, trusted religious leaders, community champions—possibly tapping into the infrastructure developed from COVID-19 vaccine rollout—and community groups, including collaborators such as Bromley-by-Bow. This will enable broader community input into the final project outputs. All findings will be publicly available via our website in accessible forms for lay consumption with assistance from our PAG.

## Results

The CICADA project was funded by the Health and Social Care Delivery Research (HSDR) program of the National Institute for Health and Care Research (NIHR) in March 2021 and began in May 2021. Further work within the project was commissioned in March 2022. This will provide a subset of data focused on mental health specifically in Northeast England and will add Greater Manchester and the Northwest Coast to our sites, where the NIHR has identified a particular need. Data collection began in August 2021, with the last participants due to be recruited in September 2022. As of January 2022, at the close of wave 1, we had 5792 survey respondents with usable data from 4300 respondents, and had completed 227 interviews. We plan to collect 84 further interviews for the newly funded substudy. At the time of submission, beginning April 2022, we are recruiting participants for the substudy and wave 2 of the surveys and qualitative work. We expect all results to be submitted for publication by winter of 2022.

## Discussion

### Anticipated Findings and Potential Impact

In undertaking this study, we will fill a gap in the evidence about the pandemic experiences of disabled people and people living with chronic conditions, particularly those from minoritized ethnic groups. We expect to contribute considerable new knowledge through our mixed methods approach. We consider issues such as those relating to access to health and social care and resources, formal and informal networks of support, and discrimination and marginalization. However, we are particularly interested in the strengths and assets that have improved our participants’ capacity to cope with the pandemic.

We believe this is important, as many iniquitous pandemic health and well-being challenges, such as those faced by minoritized ethnic groups at the intersection with chronic conditions/impairments and insecure citizenship status, can be mitigated by small adjustments to health and social care service policy and delivery, formal networks such as community health services, and informal networks such as family and friends [25]. We expect to provide recommendations for these adjustments and for potential interventions through our longitudinal mixed methods analyses. We may also produce some simple interventions ourselves. To attempt to tease out the impact of the pandemic, we will: (1) model relationships between mediating variables (including social network features) and health and social outcomes; and (2) explore participant current and recent experiences, and recall of prepandemic experiences and inequities.

### Building on Prior Research

We are undertaking both primary research and secondary data analyses. While the design and focus of our study are unique, the pandemic has fostered the development of a number of contemporary studies looking at particular disabilities, particular “stakeholders” in disability experiences (eg, disabled people, health and social care services staff, carers, young people), and particular racial and ethnic groups. Our scoping review, which began in 2021 and includes grey literature, will be updated in autumn 2022 to ensure our findings are reported in the context of these other studies. We will publish our reviews. Our secondary analyses of other panel data will help to contextualize our own findings; for example, we are analyzing data from a survey that began before but overlapped with our own, which also includes some relevant data.

### Strengths and Limitations

We will provide rich quantitative and qualitative data, with a large sample size for qualitative interviews, providing in-depth information through quota sampling. Our creative participatory and equitable approach will be key to cocreating our outputs with relevant stakeholders. This will include members of the populations we hope will benefit, third-sector organizations, clinicians, social care staff, and policy staff. This will ensure outputs that have real credibility, real-world relevance and value, can be implemented, and are sustainable.

Although the ideal study design would include an experimental evaluation of outputs, we cannot undertake full feasibility testing and trialing of any interventions we suggest, as this is an 18-month study. We may, however, explore implementation enablers and barriers and acceptability in small proof-of-concept evaluations; these are likely to require ethical review amendment.

We are not using randomized sampling in any part of the study, which is likely to introduce bias. However, rigorous synthesis of the multiple types of data we produce, our strong patient representation, our participatory approach across stakeholder groups, our overall rigor and adherence to principles of Open Science, and a reflexive approach to biases should help to contextualize findings within these limitations.

We focus on specific minoritized ethnic groups and specific sites within England in our qualitative work. This has benefits in terms of the depth of analysis for particular groups and settings, but could reduce transferability of findings to other groups and settings, which we will explore through our survey and other existing data sets. Categorizations are laden with assumptions that need to be explored.

The survey, being primarily digital, will exclude people with poor access to the digital world, although we do offer alternatives such as paper-based copies. There is also the potential for pandemic survey fatigue.

In studying the experiences of disabled people with impairments and those living with chronic conditions who come from certain minoritized ethnic groups, we are aiming for transformative research. We are sensitive to the social constructionist nature of terms that are used to categorize particular groups, which can result in tensions. However, we need to disseminate our findings using terms that have meaning to our key audiences. We intend to report on the issues and tensions as part of our wider push for change.

### Conclusions

Current understandings and considerations are limited with regard to the health and social care and support received by disabled people or those living with chronic conditions who are from certain minoritized ethnic groups. Inequities existing before the pandemic have been made worse by it, and public and policy awareness of this exacerbation provides an opportunity for change. This study, using an intersectional assets-based approach and drawing on participatory and mixed methods, aims to fill a gap in the evidence to help inform changes that reduce inequities.

## Appendix

Multimedia Appendix 1 Peer-review report by the National Institute for Health Research.

## Abbreviations

ADS: Anxiety and Depression Scale
BiB: Born in Bradford
CFIR: Consolidated Framework for Implementation Research
CICADA: Coronavirus Chronic Conditions and Disabilities Awareness Study
CLS: Centre for Longitudinal Research (London)
HSDR: Health and Social Care Delivery Research
LGM: latent growth modeling
NHS: National Health Service
NIHR: National Institute for Health and Care Research
PAG: patient advisory group
PerSPectif: Perspective, Setting, Phenomenon of interest/problem, Environment, (optional Comparison), Time/timing, Findings
PRISMA: Preferred Reporting Items for Systematic Reviews and Meta-analyses
QOSC-ID: World Health Organization Quality of Care and Support-Disability
SEM: structural equation modeling
SPIDER: Sample, Phenomenon of Interest, Design, Evaluation topics, Research source, Setting
WHO: World Health Organization
WHOQOL-BREF-ID: World Health Organization Quality of Life

## References

[1] Orcutt M, Spiegel P, Kumar B, Abubakar I, Clark J, Horton R, Lancet Migration (2020). Lancet Migration: global collaboration to advance migration health. Lancet.

[2] Kuper H, Banks LM, Bright T, Davey C, Shakespeare T (2020). Disability-inclusive COVID-19 response: What it is, why it is important and what we can learn from the United Kingdom's response. Wellcome Open Res.

[3] Abuelgasim E, Saw LJ, Shirke M, Zeinah M, Harky A (2020). COVID-19: Unique public health issues facing Black, Asian and minority ethnic communities. Curr Probl Cardiol.

[4] Spencer A, Watermeyer B, Rogers A (2022). The importance of tackling the social determinants of health to address the unmet need within cancer services. Reflections from build back fairer: the COVID-19 Marmot Review. Clin Oncol (R Coll Radiol).

[5] Mathur R, Rentsch CT, Morton CE, Hulme WJ, Schultze A, MacKenna B, Eggo RM, Bhaskaran K, Wong AYS, Williamson EJ, Forbes H, Wing K, McDonald HI, Bates C, Bacon S, Walker AJ, Evans D, Inglesby P, Mehrkar A, Curtis HJ, DeVito NJ, Croker R, Drysdale H, Cockburn J, Parry J, Hester F, Harper S, Douglas IJ, Tomlinson L, Evans SJW, Grieve R, Harrison D, Rowan K, Khunti K, Chaturvedi N, Smeeth L, Goldacre B, OpenSAFELY Collaborative (2021). Ethnic differences in SARS-CoV-2 infection and COVID-19-related hospitalisation, intensive care unit admission, and death in 17 million adults in England: an observational cohort study using the OpenSAFELY platform. Lancet.

[6] Mirza M (2011). New issues in refugee research. Unmet needs and diminished opportunities: disability, displacement and humanitarian healthcare, Research report 212. UNHCR United Nations Refugee Agency.

[7] Clarke SK, Kumar GS, Sutton J, Atem J, Banerji A, Brindamour M, Geltman P, Zaaeed N (2021). Potential impact of COVID-19 on recently resettled refugee populations in the United States and Canada: perspectives of refugee healthcare providers. J Immigr Minor Health.

[8] Putz C, Ainsl D Coronavirus (COVID-19) related deaths by disability status, England, Wales March to 14 July 2020. Office for National Statistics.

[9] Harding S, Balarajan R (2000). Limiting long-term illness among black Caribbeans, black Africans, Indians, Pakistanis, Bangladeshis and Chinese born in the UK. Ethn Health.

[10] Razaq A BAME COVID-19 deaths? What do we know? Rapid Data Evidence Review. Centre for Evidence-Based Medicine.

[11] Thomason ME, Hendrix CL, Werchan D, Brito NH (2021). Social determinants of health exacerbate disparities in COVID-19 illness severity and lasting symptom complaints. medRxiv.

[12] Fang ML, Sixsmith J, Lawthom R, Mountian I, Shahrin A (2015). Experiencing 'pathologized presence and normalized absence'; understanding health related experiences and access to health care among Iraqi and Somali asylum seekers, refugees and persons without legal status. BMC Public Health.

[13] Iob E, Frank P, Steptoe A, Fancourt D (2020). Levels of severity of depressive symptoms among at-risk groups in the UK during the COVID-19 pandemic. JAMA Netw Open.

[14] Sheridan Rains L, Johnson S, Barnett P, Steare T, Needle JJ, Carr S, Lever Taylor B, Bentivegna F, Edbrooke-Childs J, Scott HR, Rees J, Shah P, Lomani J, Chipp B, Barber N, Dedat Z, Oram S, Morant N, Simpson A, COVID-19 Mental Health Policy Research Unit Group (2021). Early impacts of the COVID-19 pandemic on mental health care and on people with mental health conditions: framework synthesis of international experiences and responses. Soc Psychiatry Psychiatr Epidemiol.

[15] Mehta B, Jannat-Khah D, Fontana MA, Moezinia CJ, Mancuso CA, Bass AR, Antao VC, Gibofsky A, Goodman SM, Ibrahim S (2020). Impact of COVID-19 on vulnerable patients with rheumatic disease: results of a worldwide survey. RMD Open.

[16] CICADA: Coronavirus Chronic Conditions and Disabilities Awareness Study.

[17] Boldrini P, Garcea M, Brichetto G, Reale N, Tonolo S, Falabella V, Fedeli F, Cnops AA, Kiekens C (2020). Living with a disability during the pandemic. "Instant paper from the field" on rehabilitation answers to the COVID-19 emergency. Eur J Phys Rehabil Med.

[18] (2021). Addressing the impact of COVID-19 on the lives of people living with neurological disorders. European Federation of Neurological Associations (EFNA).

[19] Le Cam Y (2017). Results of the H-CARE Pilot Survey for the development of a validated scale and of a Common Feedback Mechanism to measure healthcare experience for rare diseases in Europe. EURORDIS-Rare Diseases Europe.

[20] Fiona Weir on behalf of the National Voices coalition What we know now: What people with health and care needs experienced during the first wave of COVID-19 A rapid review of data from over 66,000 responses to National Voices members? surveys, September 2020, National Voices coalition. National Voices.

[21] Cheong J, Goh Z, Marras C, Tanner C, Kasten M, Noyce A, Movement Disorders Society Epidemiology Study Group (2020). The Impact of COVID-19 on access to Parkinson's disease medication. Mov Disord.

[22] Al-Hashel J, Ismail I (2020). Impact of coronavirus disease 2019 (COVID-19) pandemic on patients with migraine: a web-based survey study. J Headache Pain.

[23] Ziadé N, el Kibbi L, Hmamouchi I, Abdulateef N, Halabi H, Hamdi W, Abutiban F, el Rakawi M, Eissa M, Masri B (2020). Impact of the COVID‐19 pandemic on patients with chronic rheumatic diseases: a study in 15 Arab countries. Int J Rheum Dis.

[24] Baranidharan G, Bretherton B, Eldabe S, Mehta V, Thomson S, Sharma ML, Vajramani G, Bojanic S, Gulve A, FitzGerald J, Hall S, Firth J (2021). The impact of the COVID-19 pandemic on patients awaiting spinal cord stimulation surgery in the United Kingdom: a multi-centre patient survey. Br J Pain.

[25] Loubaba M, Jones T (2020). The impact of COVID-19 on black, Asian and minority ethnic communities, NIHR special report, 20/05/2020, UoB_COVID1. National Institute for Health Research.

[26] Anderson B Citizenship: What is it and why does it matter?. The Migration Observatory.

[27] Williams DR, Collins C (2001). Racial residential segregation: A fundamental cause of racial disparities in health. Public Health Reports.

[28] Link BG, Phelan J (1995). Social conditions as fundamental causes of disease. J Health Soc Behav.

[29] Baptist AP, Lowe D, Sarsour N, Jaffee H, Eftekhari S, Carpenter LM, Bansal P (2020). Asthma disparities during the COVID-19 pandemic: a survey of patients and physicians. J Allergy Clin Immunol Pract.

[30] Campos-Castillo C, Anthony D (2021). Racial and ethnic differences in self-reported telehealth use during the COVID-19 pandemic: a secondary analysis of a US survey of internet users from late March. J Am Med Inform Assoc.

[31] Miller P, Parker S, Gillinson S (2004). Disablism: How to tackle the last prejudice.

[32] Crenshaw K (1989). Demarginalizing the intersection of race and sex: a Black feminist critique of antidiscrimination doctrine, feminist theory and antiracist politics. Univ Chic Leg Forum.

[33] Bronfenbrenner U (1986). Ecology of the family as a context for human development: research perspectives. Dev Psychol.

[34] Heise L L (1998). Violence against women: an integrated, ecological framework. Violence Against Women.

[35] Rivas C, Tomomatsu I, Gough D (2021). The many faces of disability in evidence for policy and practice: embracing complexity. Evid policy.

[36] Casanova EL, Widman CJ (2021). A sociological treatment exploring the medical model in relation to the neurodiversity movement with reference to policy and practice. Evid Policy.

[37] Oliver M (2013). The social model of disability: thirty years on. Disabil Soc.

[38] Goodley D (2018). The Dis/ability Complex. J Divers Gender Stud.

[39] Bunbury S (2019). Unconscious bias and the medical model: How the social model may hold the key to transformative thinking about disability discrimination. Int J Discrim Law.

[40] Resolution adopted by the General Assembly on 13 December 2006. 61/106. Convention on the Rights of Persons with Disabilities. United Nations.

[41] Siebers T (2008). Disability theory.

[42] Arendt H (2003). Responsibility and judgement.

[43] Cole ER (2009). Intersectionality and research in psychology. Am Psychol.

[44] Collins P (1990). Black feminist thought: knowledge, consciousness, and the politics of empowerment.

[45] Crenshaw K (1991). Mapping the Margins: intersectionality, identity politics, and violence against women of color. Stanford Law Review.

[46] Shulz AJ, Mullings L (2016). Gender, race, class & health: intersectional approaches.

[47] Bernstein KS, Park S, Shin J, Cho S, Park Y (2011). Acculturation, discrimination and depressive symptoms among Korean immigrants in New York City. Community Ment Health J.

[48] Frost DM (2020). Hostile and harmful: Structural stigma and minority stress explain increased anxiety among migrants living in the United Kingdom after the Brexit referendum. J Consult Clin Psychol.

[49] Martynowska K, Korulczyk T, Mamcarz PJ (2020). Perceived stress and well-being of Polish migrants in the UK after Brexit vote. PLoS One.

[50] Gee G, Ryan A, Laflamme DJ, Holt J (2006). Self-reported discrimination and mental health status among African descendants, Mexican Americans, and other Latinos in the New Hampshire REACH 2010 Initiative: the added dimension of immigration. Am J Public Health.

[51] Sanchez GJ (2018). Face the nation: race, immigration, and the rise of nativism in late twentieth century America. Int Migration Rev.

[52] Kirkup J (2012). Theresa May interview: 'We're going to give illegal migrants a really hostile reception'. The Telegraph.

[53] Viruell-Fuentes EA (2011). “It's a lot of work”. Du Bois Rev.

[54] Naber N (2010). Ambiguous insiders: an investigation of Arab American invisibility. Ethnic Racial Stud.

[55] McLeroy KR, Bibeau D, Steckler A, Glanz K (1988). An ecological perspective on health promotion programs. Health Educ Q.

[56] (2020). Integrating care: Next steps to building strong and effective integrated care systems across England. NHS England.

[57] Bronfenbrenner U, Ceci SJ (1994). Nature-nurture reconceptualized in developmental perspective: a bioecological model. Psychol Rev.

[58] Keith R, Crosson J, O?Malley A (2017). Using the Consolidated Framework for Implementation Research (CFIR) to produce actionable findings: a rapid-cycle evaluation approach to improving implementation. Implementation Sci.

[59] Umucu E, Tansey T, Brooks J, Lee B (2020). The protective role of character strengths in COVID-19 stress and well-being in individuals with chronic conditions and disabilities: an exploratory study. Rehab Counsel Bull.

[60] Ciaffi J, Brusi V, Lisi L, Mancarella L, D'Onghia M, Quaranta E, Bruni A, Spinella A, Giuggioli D, Landini MP, Ferri C, Meliconi R, Ursini F (2020). Living with arthritis: a "training camp" for coping with stressful events? A survey on resilience of arthritis patients following the COVID-19 pandemic. Clin Rheumatol.

[61] Brewer G, Stratton K (2020). Living with chronic fatigue syndrome during lockdown and a global pandemic. Fatigue: Biomed Health Behav.

[62] Foley W, Schubert L (2013). Applying strengths-based approaches to nutrition research and interventions in Indigenous Australian communities. J Critical Dietetics.

[63] Antonovsky A (1996). The salutogenic model as a theory to guide health promotion. Health Promot Int.

[64] Morgan A, Ziglio E (2007). Revitalising the evidence base for public health: an assets model. Promot Educ.

[65] Hombrados-Mendieta I, Millán-Franco M, Gómez-Jacinto L, Gonzalez-Castro F, Martos-Méndez MJ, García-Cid A (2019). Positive influences of social support on sense of community, life satisfaction and the health of immigrants in Spain. Front Psychol.

[66] Wilkinson M, Dumontier M, Aalbersberg IJJ, Appleton G, Axton M, Baak A, Blomberg N, Boiten JW, da Silva Santos LB, Bourne PE, Bouwman J, Brookes AJ, Clark T, Crosas M, Dillo I, Dumon O, Edmunds S, Evelo CT, Finkers R, Gonzalez-Beltran A, Gray AJG, Groth P, Goble C, Grethe JS, Heringa J, 't Hoen PAC, Hooft R, Kuhn T, Kok R, Kok J, Lusher SJ, Martone ME, Mons A, Packer AL, Persson B, Rocca-Serra P, Roos M, van Schaik R, Sansone SA, Schultes E, Sengstag T, Slater T, Strawn G, Swertz MA, Thompson M, van der Lei J, van Mulligen E, Velterop J, Waagmeester A, Wittenburg P, Wolstencroft K, Zhao J, Mons B (2016). The FAIR Guiding Principles for scientific data management and stewardship. Sci Data.

[67] (2020). After-care needs of inpatients recovering from COVID-19. NHS England.

[68] Iyengar K, Jain VVR, Vaishya R (2020). Pitfalls in telemedicine consultations in the era of COVID 19 and how to avoid them. Diabetes Metab Syndr.

[69] Cavagna L, Zanframundo G, Codullo V, Pisu MG, Caporali R, Montecucco C (2021). Telemedicine in rheumatology: a reliable approach beyond the pandemic. Rheumatology.

[70] Glover RE, van Schalkwyk MC, Akl EA, Kristjannson E, Lotfi T, Petkovic J, Petticrew MP, Pottie K, Tugwell P, Welch V (2020). A framework for identifying and mitigating the equity harms of COVID-19 policy interventions. J Clin Epidemiol.

[71] Aksoy O, Bann D, Fluharty M, Nandi A (2022). Religiosity and mental wellbeing among members of majority and minority religions: Findings from Understanding Society: the UK Household Longitudinal Study. Am J Epidemiol.

[72] WHOQOL Disabilities Group (2011). WHO QOL Disabilities.

[73] (2016). Vulnerability Assessment Framework Questionnaire Validation Workshop Summary. UNHCR.

[74] Stansfeld S, Marmot M (1992). Deriving a survey measure of social support: the reliability and validity of the close persons questionnaire. Soc Sci Med.

[75] Sharma VK, Lepping P, Cummins AGP, Copeland JRM, Parhee R, Mottram P (2004). The Global Mental Health Assessment Tool--Primary Care Version (GMHAT/PC). Development, reliability and validity. World Psychiatry.

[76] Templer DI (1970). The construction and validation of a Death Anxiety Scale. J Gen Psychol.

[77] Cooke A, Smith D, Booth A (2012). Beyond PICO: the SPIDER tool for qualitative evidence synthesis. Qual Health Res.

[78] Wright J, Hayward A, West J, Pickett K, McEachan RM, Mon-Williams M, Christie N, Vaughan L, Sheringham J, Haklay M, Sheard L, Dickerson J, Barber S, Small N, Cookson R, Garnett P, Bywater T, Pleace N, Brunner EJ, Cameron C, Ucci M, Cummins S, Fancourt D, Kandt J, Longley P, Morris S, Ploubidis G, Savage R, Aldridge R, Hopewell D, Yang T, Mason D, Santorelli G, Romano R, Bryant M, Crosby L, Sheldon T (2019). ActEarly: a City Collaboratory approach to early promotion of good health and wellbeing. Wellcome Open Res.

[79] Wolf EJ, Harrington KM, Clark SL, Miller MW (2013). Sample size requirements for structural equation models: an evaluation of power, bias, and solution propriety. Educ Psychol Meas.

[80] Powell W, Smith-Doerr LNL, Smelser NJ, Swedberg R (1994). Networks and economic life. The Handbook of Economic Sociology.

[81] Valente TW (2010). Social networks and health: models, methods, and applications.

[82] Calderwood L (2016). Reducing non-response in longitudinal surveys by improving survey practice, PhD thesis. University College London.

[83] Enders C (2010). Applied missing data analysis.

[84] Shim J, Darling K, Lappe M, Thomson L, Lee S, Hiatt R, Ackerman S (2014). Homogeneity and heterogeneity as situational properties: producing--and moving beyond?--race in post-genomic science. Soc Stud Sci.

[85] Decary S, Dugas M, Stefan T, Langlois L, Skidmore B, Bhe?reur A, LeBlanc A (2021). Care Models for Long COVID - A Rapid Systematic Review. SPOR Evidence Alliance, COVID-END Network.

[86] Sutherland C, Cheng Y (2009). Participatory-action research with (im)migrant women in two small Canadian cities: using photovoice in Kingston and Peterborough, Ontario. J Immigrant Refugee Stud.

[87] Oteros-Rozas E, Martín-López B, Daw TM, Bohensky EL, Butler JR, Hill R, Martin-Ortega J, Quinlan A, Ravera F, Ruiz-Mallén I, Thyresson M, Mistry J, Palomo I, Peterson GD, Plieninger T, Waylen KA, Beach DM, Bohnet IC, Hamann M, Hanspach J, Hubacek K, Lavorel S, Vilardy SP (2015). Participatory scenario planning in place-based social-ecological research: insights and experiences from 23 case studies. Ecol Soc.

[88] The Collaborative Poetics Network (2018). The Collaborative Poetics Network Resource Pack.

[89] The Co-Creating Welfare Project partners (2019). Co-creating Welfare: Training course material preparing professionals to co-create welfare solutions with citizens, 2019, Universidade do Minho. Instituto de Educação.

[90] Thomson A, Rivas C, Giovannoni G (2015). Multiple sclerosis outpatient future groups: improving the quality of participant interaction and ideation tools within service improvement activities. BMC Health Serv Res.

[91] (2018). Patient Engagement Keystone Summit 2018 White Paper. KLAS research.

[92] Booth A, Noyes J, Flemming K, Moore G, Tunçalp Ö, Shakibazadeh E (2019). Formulating questions to explore complex interventions within qualitative evidence synthesis. BMJ Glob Health.

