# Supplementary material for: Lessons From the COVID-19 Pandemic to Improve the Health, Social Care, and Well-being of Minoritized Ethnic Groups With Chronic Conditions or Impairments: Protocol for a Mixed Methods Study
Source: JMIR Res Protoc. 2022 Jul 1;11(7):e38361. doi: 10.2196/38361 (PMC9255363; doi:10.2196/38361)
Supplement: Multimedia Appendix 1 [file resprot_v11i6e38361_app1.pdf]

NIHR | National Institute for Health Research

NIHR Health Services and Delivery Research Programme

APPLICATION SUMMARY INFORMATION

**Programme Name**	HS&DR
**Funding Opportunity**	HS&DR Researcher Led
**Call**	20/02 April 2020 HS&DR Researcher-led (Standard)
**Contracting Organisation**	University College London

**Research Title**
CICADA-ME: Coronavirus Intersectionalities: Chronic Conditions and Disabilities And Migrants and other Ethnic minorities

**Research Type**	Primary Research
**Proposed start date, end date (duration)**	From: 01/04/2021 to: 30/09/2022 (18 months)
**Total research costs (not including NHS Support & Treatment Costs)**	£692,489.96
**Total NHS support & treatment costs**	£0.00
**Total Non-NHS intervention costs**	£0.00

LEAD APPLICANT DETAILS & CV

**Details of Lead Applicant**	Dr Carol Anne Rivas
**Job Position**	Associate Professor in Social Policy & Programme Evaluation
**Department**	UCL Social Research Institute, Institute of Education
**Email / Phone**	c.rivas@ucl.ac.uk 07803850273
**Organisation**	University College London

Lead Applicant Information – Qualifications

Degree / professional qualification - subject	Awarding body - date of award
Other - PGCAP	Higher Education Academy - 01/11/2013
BSc (Hons) - Zoology	Queen Mary University of London - 30/06/1981
MSc - Cognitive Neuropsychology specialising in autism	Birkbeck University - 30/06/1993
PhD - Medical Sociology	Queen Mary University of London - 01/04/2012

Lead Applicant Information – Recent Relevant Publications

Lead Applicant Information – Recent Relevant Publications

1. Goff, L.M., Moore, A., Harding, S., Rivas, C. (2020). Providing culturally sensitive diabetes self-management education and support for black African and Caribbean communities: a qualitative exploration of the challenges experienced by healthcare practitioners in inner London. BMJ Open Diabetes Research and Care, ;8:e001818. doi: 10.1136/bmjdrc-2020-001818

2. Ball, E., Newton, S., Rohricht, F., Steed, L., Birch, J., Dodds, J., . . . Rivas, C. (2020). mHealth: providing a mindfulness app for women with chronic pelvic pain in gynaecology outpatient clinics: qualitative data analysis of user experience and lessons learnt. BMJ Open, 10(3), e030711. doi:10.1136/bmjopen-2019-030711

3. Steed, L., Sohanpal, R., Todd, A., Madurasinghe, V. W., Rivas, C., Edwards, E. A., . . . Walton, R. T. (2019). Community pharmacy interventions for health promotion: effects on professional practice and health outcomes. Cochrane Database Syst Rev, 12, CD011207. doi:10.1002/14651858.CD011207.pub2

4. Moore, A. P., Rivas, C. A., Stanton-Fay, S., Harding, S., & Goff, L. M. (2019). Designing the Healthy Eating and Active Lifestyles for Diabetes (HEAL-D) self-management and support programme for UK African and Caribbean communities: a culturally tailored, complex intervention under-pinned by behaviour change theory. BMC Public Health, 19(1), 1146. doi:10.1186/s12889-019-7411-z

5. Wagland, R., Nayoan, J., Matheson, L., Rivas, C., Brett, J., Collaco, N., . . . Watson, E. (2019). Adjustment strategies amongst black African and black Caribbean men following treatment for prostate cancer: Findings from the Life After Prostate Cancer Diagnosis (LAPCD) study. European Journal of Cancer Care, 9 pages. doi:10.1111/ecc.13183

6. Rivas, C., Vigurs, C., Cameron, J., & Yeo, L. (2019). A realist review of which advocacy interventions work for which abused women under what circumstances. Cochrane Database Syst Rev, 6, CD013135. doi:10.1002/14651858.CD013135.pub2

Lead Applicant Information – Research Grants Held

£10,000 2020 Rivas (PI), Roberts, Yamamoto, Tomomatsu. CHIA: children made vulnerable through health conditions and disability, an international exploration of relevant issues and assets during the pandemic. Osaka-UCL Global Partnership

£78,000 2020 Rivas (mentor), Totsika Paulauskaite Developing a COS for relationship and sex education research and learning disabilities. ESRC Studentship

£3,000 2020 Rivas (PI). Collaboration to develop UNESCO partnership in Global Health and Education, Osaka. UCL International Funds

£50,000 2020 Mintz J et al. (Co-I) Enhancing teacher decision-making for children with autism using machine learning. IOE Collaborative Seed Funding

£6,000 2019 Ball, Rivas et al. Co-producing a laparoscopy consent video: a testimony. SME

£10,000 2019 Shucan-Bird, Rivas, Tomson (Co-I). Developing shared understanding of domestic violence evidence to inform policing. UCL Knowledge Exchange Fund

£165,595 2018 Ball E et al. (Co-PI).CRESCENDO: Creating a Clinical Prediction Model to predict Surgical Success in Endometriosis. NIHR RFPB

£76,000 2018 Rivas (mentor), Schucan-Bird, Dyke. Underlying factors affecting probation officer decisions about the risk posed by perpetrators of domestic violence and other abuse. ESRC

£3,000 2018 Rivas (PI). Santander Catalyst Award (Chile collaboration seed grant). Santander-UCL South America

£10,000 2017 Rivas (PI), Vigurs. Realist review of domestic violence advocacy interventions. NIHR Cochrane I2017 (ref. 17/62/37)

£2,000 2017 Johnson, Rivas et al. (Co-I). Collaborative poetics network for social justice. ISRF Networks Grant

£5,000 2017 Thompson, Rivas, Giavononni. #Thinkhand (multiple sclerosis). Barts Charity

£51,861 2017 Giovanonni et al (Co-I). NHS Small group clinics, multiple sclerosis. Barts Health Charity

£598,623 2017-20 Goff, Rivas (mentor, Co-I). Healthy Eating and Active Lifestyles for Diabetes (HEAL-

Lead Applicant Information – Research Grants Held
D) External: NIHR Senior CDA
£231,461 2016-19 Ball E et al (Co-PI). Mindfulness meditation using a smart-phone app for women with chronic pelvic pain. NIHR RfPB
£412,242 2015-17 Rivas (CI) et al. PRESENT: Patient Reported Experience Survey Engineering of Natural Text. NIHR HS&DR
£280,000 2014-20 Cooper, Rivas (Co-PI). Core outcome sets in gynaecology cancers. CRUK
£4,000 2015 Edwards, Rivas, Walton. Cigbreak developing and testing a smoking cessation app in the form of a game http://www.healthygames.co.uk/ Bart's Charity ACF Grant, Number 8582294
£4,000 2014 Duffy (Rivas and Khan mentees) Developing Core Outcome Sets for Clinical Trials in Obstetrics and Gynaecology Research (DOCTOR) – A Pump Priming Study to Achieve Successful Fellowship Application. Barts and the London charity Clinical Academic Research Fellowship awards
£190,000 2013-16 Duncan, Goff, Rivas (mentor Co-I). Prevention of Type 2 Diabetes in HIV: An Effectiveness and Acceptability Study. NIHR CRTF PhD
£60,000 2013-2015 Mentor to Neha Patel and Dr Alex Sohal. Domestic violence screening in sexual health clinics (IRIS project); Qualitative exploration of implementation of advocacy for abused women in sexual health clinics, framed by Normalisation Process Theory. Social Enterprise Funding https://irisi.org/

PREVIOUS APPLICATION HISTORY

Relevant NETS Programmes previous application information (within the last 3 years)

Application Type: Stage 2 Application GDPR

Programme: Crossprogramme

Project Title: NIHR132222 - CICADA-ME: Coronavirus Intersectionalities: Chronic Conditions and Disabilities And Migrants and other Ethnic minorities

Role: Chief Investigator   Outcome: Do Not Progress Application

Please indicate how your current research proposal differs from this previous application:

The previous application is largely similar but:

1. We have added several more clinicians and lay and community members, and stronger pathways to impact as well as two proof-of-concept training programme evaluations at study end.

2. This new application also takes account of changes in the pandemic, and in policy and practice and new Covid research findings. This means we now include secondary analysis of existing surveys for which we have access and have included members of the ActEarly consortium who provide increased process support for actualisation, reach and impact.

3. We have more emphasis on long covid.

If unsuccessful, please indicate why:

Out of remit as call intended to focus on Covid-19 per se not impacts of the pandemic on other conditions. Advised to resubmit to this current call.

Advised to include clearer paths to impact.

Other funders previous application information

Application Type: Outline Application

Funding Body: NIHR   Funding Scheme: RP (PCR-03-C19) Recovery, Renewal, Reset: Research to inform policyresponses to COVID-19

Project Title: 28982 - CICADA-NET: Coronavirus Intersectionalities: Chronic Conditions and Disabilities Networks for ethnic minorities

Outcome: Not Funded   Date of Outcome:

Please indicate how your current research proposal differs from this previous application:

This is a smaller single wave version of the current proposal that focused specifically on the PRP call aims and on social network analysis (and therefore also has less broad pathways to impact). If this were funded I would expect costs and activities for the current proposal to reduce across WP1 and wave 1 of WP1 and 2 but not to be eradicated.

If unsuccessful, please indicate why:

APPLICATION BACKGROUND

**Was this application submitted in response to a highlight notice?**	No

Highlight Notice:

**Previous Research Info**

CLINICAL TRIALS

Clinical Trials Unit's (CTU) Participation

**Is a Clinical Trials Unit involved with this research proposal?**	No

**Clinical Trials Unit's Information**

**If applicable, please describe how you have worked with a Clinical Trials Unit in developing you application and what support they will provide if funding is approved.**
N/a

**If a Clinical Trials Unit is not being used, please explain why and who/what will be involved instead, if applicable to this application.**
N/a

RESEARCH TEAM

Lead Applicant

Specify Lead Applicants role in research
lead the study, responsible for overall data collection, data management, quality control, qualitative lead in the project, accountable for the qualitative analysis, co-lead on review work

Lead Applicants % FTE Commitment	20%

Joint Lead & Co-Applicants

Name	Position Held	Role / % FTE	Department	Organisation
Dr Ozan Aksoy (Co-Applicant)	Associate Professor	Quantitative analysis co-lead, social network analysis lead<br><br>FTE - 20%	Social Research Institute	University College London
Mr Bilal Nasim (Co-Applicant)	Research Data Scientist	Co-lead quantitative analysis<br><br>FTE - 5%	Social Research Institute	University College London
Dr Ruth Dobson (Co-Applicant)	Clinical Lead for Neurology, Barts Health	Co-lead on clinical pathways to impact, lead on neuroscience disabilities<br><br>FTE - 5%	Wolfson Institute of Preventive Medicine	Queen Mary University of London
Dr Alison Thomson (Co-Applicant)	Lecturer in Patient Public Involvement and Public Engagement in Science	Lead on participatory work, co-lead London recruitment<br><br>FTE - 5%	Wolfson Institute of Preventive Medicine	Queen Mary University of London
Dr Jessica Eccles (Co-Applicant)	Clinical Senior Lecturer and MQ Arthritis Research UK Fellow	Co-lead on clinical pathways to impact, lead on liaison psychiatry and chronic pain disabilities<br><br>FTE - 5%	Trafford Centre for Medical Research,	Brighton and Sussex Medical School
Dr Louise Goff (Co-Applicant)	Reader in Nutritional Sciences	Lead in engaging BAME of African descent, and diet issues, and co-lead in co-design	Nutrition & Dietetics	King's College London

Name	Position Held	Role / % FTE	Department	Organisation
		FTE - 5%		
Dr Victoria Redclift (Co-Applicant)	Associate Professor of Political Sociology	Lead on migrants, citizenship and the intersections of 'race', class and gender, Canterbury recruit<br><br>FTE - 5%	Social Research Institute	University College London
Dr Josie Dickerson (Co-Applicant)	Director - Better Start Bradford Innovation Hub and Bradford Inequalities Research Unit	Lead for recruitment at the Bradford site. Expertise in engaging seldom heard communities<br><br>FTE - 10%	Better Start Bradford Innovation Hub and Bradford Inequalities Research Unit	Bradford Teaching Hospitals NHS Foundation Trust
Dr Elizabeth Ball (Co-Applicant)	Consultant	Leading on women's health issues and co-lead on clinical impact<br><br>FTE - 5%	Women’s Health Research Unit	Queen Mary University of London
Dr Vadivelu SARAVANAN, (Co-Applicant)	Contact Rheumatologist	Lead on rheumatology and co-lead on clinical impact<br><br>FTE - 5%	Rheumatology	Gateshead Health NHS Foundation Trust
Ms Jenny Camaradou (Co-Applicant – PPI)	PPI	Co-lead on PPI work<br><br>FTE - 5%	PPI	PPI Representative Based in England
Dr Sarabajaya Kumar (Co-Applicant – PPI)	PPI lead	PPI lead<br><br>FTE - 5%	Political Science	University College London

Joint Lead Applicant

Justification for Joint Lead Applicant

Relevant expertise and experience of Joint Lead Applicant

Supporting Roles

Name	Position Held	Role	Organisation	Agreed
Ms Claire Bensusan	Assistant Director, Research Support & Development	Administrative Authority or Finance Office	University College London<br><br>UCL Research Services	18/12/20
Professor Toby Seddon	Professor of Social Science and Head of Department	Head of Department	University College London<br><br>Social Research Institute, IoE	17/12/20
Professor Toby Seddon	Professor of Social Science and Head of Department	Sponsor	University College London<br><br>UCL Social Research Institute	17/12/20

**Please declare any conflicts or potential conflicts of interest that you or your research team may have in undertaking this research, including any relevant, non-personal & commercial interests that could be perceived as a conflict of interest.**
None to declare.

SCIENTIFIC ABSTRACT & PLAIN ENGLISH SUMMARY

Scientific Abstract

Two groups that experience similar societal inequities (expanded by the COVID-19 pandemic), including in social and health care, are people with chronic conditions/disabilities (PwCD) and ethnic minorities. The worst affected are both ethnic minority AND with chronic conditions/disabilities, a common group, as COVID-19 mortality statistics show. There is a largely unmet expressed need to explore this combined group's pandemic experiences with new or worsening conditions/disabilities including post-Covid syndrome in relation to reduced services, inequalities, lifestyle changes or health neglect and vaccine uptake. We aim to contribute and inform evidence-based formal and informal strategies, guidelines, recommendations and easily adopted interventions for pandemic-related and future health and social care policy and practice, to mitigate inequities and improve the experiences, health and wellbeing outcomes of minority ethnic groups at the intersection with chronic conditions/disabilities. To do so, we will develop a rich intersectional understanding of their mental and physical health, coping, access to resources, and informal and formal social and health care support experiences, and relevant assets and strengths, longitudinally over 18 months using mixed methods. Our 4 work packages involve a new UK survey in 3 waves, parallel qualitative insights, secondary analyses of other surveys, rapid review, and outputs for immediate use developed with participatory methods, with co-create workshops involving our PPI team and other stakeholders throughout. Our survey (n=5000) samples for 1st and 2nd generation community-dwelling minority ethnic groups and white British comparators, all with/without chronic conditions/disabilities, across the UK's 4 nations, to determine relationships between measured variables and their trajectories. After Survey Wave 1 we will interview 1st and 2nd generation ethnic minorities from Poland, India and Pakistan, sub-Saharan Africa, and the Middle East, and white British comparisons, all with and without chronic conditions/disabilities, about their pandemic experiences at 5 diverse sites in England, supporting transferability. Interviews (n=210) informed by survey analyses will include social network analysis, photovoice. We will train local lay people to help undertake these remotely; a transformative community migrant-majority research-active group will be our main London co-researcher. This group and a main co-applicant are members of an existing UKRI consortium and have undertaken complementary work at two of our sites, demonstrating the feasibility of our plans. At Waves 2 and 3 research workshops with interviewees will use video vignettes built from earlier study findings. Key informant interviews and co-create workshops will consider implementation. We will synthesise Keyword frequency analysis, Framework, discourse and narrative analyses, Latent Growth Modelling, Structural Equation Modelling, and social network analyses using tabulated evidence to decision methods, with interim findings reported at each wave for early delivery of benefits. Respondent and national UK demographic data will be compared for representativeness, and transferability explored at each stage. Data will be presented separately and combined for ethnic minorities and PwCD. We expect rapid impact from our strong networks and Co-A and collaborator existing strong pathways to influence in health and social policy and clinical practice.

Plain English Summary of Research

Many people from Black, Asian and Minority Ethnic groups (BAME), especially those with underlying (chronic) conditions/disabilities, face barriers to accessing networks of appropriate support, health and social care or vital 'resources', such as medicine and food. Around 50% lived in poverty in 2019; the pandemic has worsened their plight, highlighting the need for these barrriers to be removed. To determine where and how best to intervene to ensure this, we need to understand the problems - and successes - these groups have experienced, especially as health and social care tries to return to normal. We also need to understand what affects their vaccine uptake and if pandemic-related service changes that are retained further exclude them. Our approach is 'intersectional'. This means we recognise everyone is affected differently by the pandemic, according to the intersection (interplay) of factors such as ethnicity, citizenship, age, gender, their work, and health or disability.

To understand this complex situation, we will survey 4,000 UK BAME people and for contrast 1000 white British, 3 times over 15 months. We will compare their health, social networks (who they have

Plain English Summary of Research
contact with) and how these help or hinder them, ways they cope with pandemic changes and associated access to support, care and resources. We will consider how intersectional factors affect this.<br>After Survey 1 we will interview 210 more people in 5 diverse sites in England about the same topics, probing for coping strategies and ideas to inform health and social care policy and practice. Interviewees will also describe their networks using special brief questionnaires, photos and maps. We use remote working. We will find people for the study via social media, NHS clinics, charities, special patient and migrant groups, our own networks, and large databases of adults interested in health research across the UK.<br>We will focus on migrants from the Middle East, India, Pakistan, Poland or Africa, or whose parents were born there, as the most likely to have problems (e.g. to have limited citizenship rights or to die from COVID-19). We will look at the impact of also having a chronic condition/disability including 'long covid'. Our PPI leads will help train local community members to do some of the research.<br>After surveys 2 and 3, interviewees will be invited to workshops to discuss findings and more recent changes. We will also review published and informal (e.g. blog) articles about pandemic BAME and disability experiences, and data from other complementary COVID-19 surveys. All our findings will be combined. Over the 18-month study we will hold 5 participatory sessions with members of different BAME communities and key informants working together to help analyse our data and co-design solutions to issues, pragmatically including ‘life hacks’ and service adaptations for rapid impact. At 16 months we will interview 15-25 key informants such as support staff and community leaders to help us put our work into immediate practice.<br>Findings and solutions will be shared as they emerge at each of the 3 data waves, for early benefit. We will report changes over time in experiences, outcomes and solutions and consider how to apply our work across the UK. We aim for immediate, readily implemented, relevant useful change in UK pandemic health and social care service delivery. We include training outputs, strong networks and Co-As with direct influence on policy and practice.

CHANGES FROM FIRST STAGE

Changes From First Stage
CHANGES IN LIGHT OF NEW RESEARCH<br>We have put a much greater emphasis on long covid, which we mentioned before but for which there is considerably more evidence since our stage 1 application. We include PPI members with long covid and a clinician who is setting up one of the 40+ long covid clinics. As well as reflecting the importance of long covid within the population, this increases our recruitment capacity as we include the considerable long covid networks that have been formed, within our processes, and the PI belongs to two such networks. One PPI co-applicant is a lay member of the NICE long covid committee.<br><br>FEEDBACK AT STAGE 1<br>This study has a welcome focus on improving the experiences and services for BAME people with chronic health conditions. The new data from the survey should provide important data of the current problems and opportunities for more responsive services. There is good attention to PPI and diversity and service-facing outputs.<br>1. This is a complex and ambitious project. It would be strengthened by a clearer overall plan showing the overarching architecture (more than the schematic on p11) giving the aims and activities for the overlapping workstreams and longitudinal work.<br>Other areas which could be clarified include:<br>2 More consideration of the survey, which forms the core of the study, as a mechanism for collecting complex information on behaviour, coping and wellbeing. This may need preliminary testing and scoping out in relation to similar exercises and instruments.<br>3 Greater detail on plans for recruitment and promotion of the survey and mitigation if the sample not reached.<br>4 More clarity on the purpose and practicalities of longitudinal survey in three waves. What are the plans given likely attrition and movement?<br>5 A clearer rationale for, and detail on qualitative research capacity for carrying out 210 interviews in WP3.<br>6 More detail on the channels and networks for target audiences in the dissemination plan.<br><br>RESPONSE TO FEEDBACK<br>1. The schematic has been revised to better indicate the overall plan.<br>2. We have given more detail on the survey, including clarifying there will be piloting but that the survey waves will be mostly made up of already validated instruments so that the main focus is on format in piloting. We have also articulated the influence of existing complementary surveys such as the Born in Bradford work from our secondary analyses.<br>3. We have provided this detail and also included more co-applicants and more focus on long covid which increase our recruitment pathways and possibilities while staying true to the study aims and rationale.<br>4. We have provided more clarity on these points. This includes indications of minimum sample size, risks and mitigation of attrition.<br>5. We have included a better explanation of these points including an explanation of the effect of theme saturation. Importantly our study includes the addition of more PPI representatives as co-applicants and collaborators costed for possible co-researcher work, and the involvement of the Bromley-by-Bow community centre, which will undertake London recruitment and interviews and which

Changes From First Stage
has experience in and is confident of recruiting the required sample. This community group is run by lay people who belong to the communities of interest and is connected to an adjacent health centre. Born in Bradford, a city collaboratory and ActEarly partner, represented by co-applicant Dickerson, has access through its set-up to relevant 'research ready' participants including co-researchers.<br>6. We have provided considerably more detail and enhanced our channels and networks through the inclusion of more clinicians, including Saravanan who is helping to set up a long covid clinic, and more co-applicants and collaborators in key positions, including a lay member of the NICE long covid committee, a lay collaborator with previous work experience in and networks with London commissioning groups (CCGs), a London community centre which focuses on the populations of interest and which also belongs to the ActEarly consortium, and also we now include explicit training programmes as outputs, which will be modifications of existing programmes based on our findings and aimed at improving wellbeing and coping in our populations of interest.<br>7. We have changed one site from Coventry to Newcastle to improve our sampling of Polish and African participants. One co-applicant is from Gateshead-Newcastle, further facilitating recruitment.<br><br>RESPONSE TO FURTHER FEEDBACK<br>1. We have simplified our screening for language fluency. Potential participants will be informed that interviews will be in English, and it is their choice as to whether they feel able to take part. This is a well formulated and effective process in Born in Bradford. Where a participant is happy to interview in English, but feels more comfortable doing so in their home language, if a researcher fluent in that language is available this will be arranged (costs of £2250 are added to Bromley-by-Bow's consultancy budget to enable this).<br>2. We include plans to explore COVID vaccine uptake.<br>3. We have expanded our description of dissemination to include more dissemination to social care and statutory services (with a Knowledge Exchange event by Bromley-by-Bow now added, at further cost of £4,250).<br>4. We have clarified the novel nature of other disseminations, the use of theory and qualitative analyses and syntheses and their relation to practice, the training details for co-researchers, the granularity of our sampling frame and the feasibility checks and mitigations for our social network analysis.<br>Overall we have slightly increased the total by £5,061.60, which includes adding the costs mentioned in points 1 and 3 and slightly reducing others in mitigation.

PATIENT & PUBLIC INVOLVEMENT

Please describe how patient and the public have been involved in developing this proposal

We had a consultation as a minuted MedAct meeting item, others with small groups or individuals by email, or online by remote video chat including members of the public, and community groups. While this excluded some groups e.g. without internet, MedAct and Bromley-by-Bow contributed and are successful in engaging these groups. As a result:

1. We focus on particular minority ethnic groups; the choice and implications on study design were discussed and PPI contributors considered citizenship status influential, which is thus included as a consideration. There is no agreed definition of the term 'migrant', usually differentiated from asylum seekers in terms of 'choice' (https://www.unhcr.org/pages/49da0e466.html; https://www.unhcr.org/uk/5d9ed32b4). A refugee has had their lack of choice formally ratified (https://www.unhcr.org/uk/5d9ed32b4). The relevance of these definitions changes over time according to our PPI work so for simplicity, despite its problems, they suggested using the term migrant to encompass all these and to mean someone who was born outside the UK and intends to stay in the UK for at least a year. Our PPI contributors will help us discuss this in our final outputs.
2. PPI contributors suggested restricting interviews to English would not reduce the impact of the study but would usefully determine barriers for those who might be assumed OK because they are not in housing for the vulnerable and can communicate in English. The PI has undertaken work on service use in minority ethnic groups with limited English language fluency* and agrees language issues need dedicated in-depth analysis and specific responses, outside the remit of this study. But as per our PPI work we will include their broader influence where relevant, following the approach of collaborators with experience in this work with relevant groups, Bromley-by-Bow and MedAct Migrants Group (most of whose research is in English). It was agreed translated study documents will be available if required to ensure fully informed consent and that if this approach excludes intended participants, interpreters will need to be be used, also PPI members said to monitor need for formats for disabilities e.g. Braille.
3. Our PPI work suggests many potential participants will prefer a cautious remote approach even when government rules allow face to face work; they may find remote work avoids time and energy costs of travel. PPI team members have also advised us it will be a long time before they would be prepared to undertake normal research work. PI Rivas has led remote qualitative pandemic research successfully e.g. for the CoGS project (see Grants, cv), June 2020 and video vignettes for the NIHR-funded study CRESCENDO, July 2020. But we can easily revert to face-to-face work if appropriate.
4. We include any self-declared chronic health condition or disability as determined through brief screening questions, including self-diagnoses as considered vital in our PPI work. Our PPI contributors approved the use of our six disability 'impact' categories after ensuring diet encompassed eating disorders but asked to ensure multisystemic conditions are represented by multisystemic impacts – thus the survey does not restrict people to choosing one or the most dominant condition, and does not restrict the number of effects (symptoms).
5. The lay abstract was checked.

* Rivas et al, 2014, The interpreted diabetes consultation. Journal of Diabetes Nursing, 18(10), 422-424)

Please describe the ways in which patients and the public will be actively involved in the proposed research, including any training and support provided

PPI will continue through the study. We have taken care to involve lay people who represent our interview population in range of ethnicities and disabilities. Our PPI team members were recruited through existing networks and also through specific condition support and third sector groups. One lay co-A has contributed to the NICE long covid committee, but this was not a condition of their recruitment, though any call for volunteers is more likely to recruit people actively engaged in this way.

1. As well as standard tasks such as checking survey questions, contributing to topic guides and

Please describe the ways in which patients and the public will be actively involved in the proposed research, including any training and support provided
advising on the study, our PPI team will be involved in co-create workshops through the study as an important part of its design, as also is the involvement of local co-researchers.<br>a. Our five co-create workshops will be led by members of our PPI working group supported by the CI and a researcher from the team trained in the methods. Materials will be provided in advance, taking account of accessibility needs (something specified in PPI work). Practical activities will aim to produce 'negotiated' analyses and outputs that empower all those attending to contribute as equals and that our PPI lead is comfortable with. These workshops should not be confused with our research workshops which will also use participatory approaches but with our research participants (though members of our PPI team will be invited to join in running these).<br>b. In each of our five recruitment areas we will train a member of our focal community to undertake interviews locally – and remotely unless it is safe to do otherwise AND this is preferred - supported by our central team which will also undertake interviews remotely. Time has been costed in for the lay co-researchers for training as well as their remuneration for the research work they do.<br>2. At the study start, our PPI team will co-develop their memorandum of association and other documents relating to their role. They will be supported in this and asked to specify their precise training/support needs, which we will provide. Co-applicants have considerable experience in this and Goff, Rivas and Thomson have won relevant awards. In particular Thomson is a recognised expert in the field and the PI has collaborated with her since 2006; we are thus pioneering in PPI and public engagement work with many years of experience and also the sensitivity that makes us open to continued learning (as at the heart of all PPI work). The PI is currently undertaking separate work on engaging with children unable to communicate verbally and therefore has expertise that is inclusive across a range of contribution platforms and needs. PI Rivas also contributed sections to the INVOLVE lay co-applicant guidance as a core contributor.<br>3. All PPI members and lay co-researchers will be recompensed at £150 per day spent on work, pro rata.<br>4. We will fully involve our PPI team in dissemination and output work with full support, and with the opportunity to write or co-author papers; the PI's' department hosts a free-for-all journal called Research for All that is an ideal platform, being fully supportive of PPI contributors.<br>5. PPI team members will also run training workshops at study end as outputs, with full support (see Outputs section of Case for support).

In rare cases where proposals do NOT involve patients and the public, clear justification must be provided
n/a

JUSTIFICATION OF COSTS

Justification of Costs
All JoR costs are at 100% amounts. TOTAL £830,940.55<br><br>Post and Salaries (all Staff) - Overall Total £322,859.23<br>PI - Dr Carol Rivas (UCL) - 20% FTE for 18 months (Sub Total £25,937.54): lead and manager across study (see Project Management in Case for Support), qualitative lead. To oversee all outputs (supported by other Co-Is), and publish 2+ standalone papers and protocol in e.g. Social Science and Medicine, 2 practitioner articles with Co-Is and TBC PDRAs.<br>Co-I Dr Ozan Oksay (UCL) – 20% FTE for 18 months (Sub Total £23,823.32): co-lead on surveys (WP2), lead on social network work (WP2, WP3), supported by a TBC PDRA.<br>Co-I Bilal Nasim (UCL) – 5% FTE for 18 months (Sub Total £4,626.29): co-lead on survey work (WP3).<br>Co-I Dr Victoria Redclift (UCL) – 5% FTE for 18 months (Sub Total £6,126.84): Lead on migrants, citizenship, intersections, and Canterbury recruitment (via Runnymede Trust<br>Academic Forum and links to community migrant groups).<br>Co-I Dr Josie Dickerson (Bradford Teaching Hospitals NHS FT) - 10% FTE for 18 months (Sub Total £12,733.40): lead for Bradford site (WP3), contributes vast expertise in engaging the seldom heard as Director of the Better Start Bradford Innovation Hub and Inequalities Research Unit and Programme Manager of the Born in Bradford (BiB) partners of the ActEarly consortium of research-ready city collaboratories. Provides network opportunities for immediate impact (WP4). Supported by 2 shared staff, Alex Newsham (Sub Total £9,087.19) and BTHFT TBA (Sub Total £16,317.57).<br>Co-I Dr Alison Thomson (QMUL) – 5% FTE for 18 months (Sub Total £5,142.00): award-winning lead on participatory work (WP3,4 and cross-study workshops), co-lead, London recruitment (WP3), dissemination.<br>Co-I Dr Louise Goff (KCL) – 5% FTE for 18 months (Sub Total £6,785.84): lead diet issues and engaging BAME of African descent (WP2,3), co-design co-lead.<br><br>Other Co-Is chosen for condition specific advice across disabilities, tasks and WP. All co-lead on pathways to impact.<br>Co-I Dr Ruth Dobson (QMUL) – 5% FTE for 18 months (Sub Total £10,118.89): lead on neuroscience disabilities, co-authoring new guidelines.<br>Co-I Dr Jessica Eccles (Brighton and Sussex Medical School) – 5% FTE for 18 months (Sub Total £9,074.00): lead on liaison psychiatry and chronic pain disabilities.<br>Co-I Dr Elizabeth Ball (Barts NHS) – 5% FTE for 18 months (Sub Total £10,104.23): lead on women's health issues<br>Co-I Dr Vadivelu Saravanan (Gateshead Health NHS Foundation Trust) – 5% FTE for 18 months (Sub Total £0, in kind): lead on rheumatology, has set up a Gateshead long Covid clinic, an important route to impact. He holds North East CRN funded research time so has no costs, adding value.<br><br>Our 2 PPI co-leads from different participant groups co-lead on study recruitment, advise on all study aspects, lead the PPI group, help run workshops, be active in the launch and study end events. Each has important networks. Costs in PPI Budget<br><br>Unnamed Researcher Posts (Staff):<br>RA1 TBC (UCL) – 1.0 FTE for 9 months (Sub Total £34,790.81), PDRA2 TBC (UCL) – 1.0 FTE for 18 months (Sub Total £74,095.65) and PDRA3 TBC (UCL) – 1.0 FTE for 18 months (Sub Total £74,095.65): TBC PDRA1 and TBC PDRA2 will drive the qualitative and quantitative work on a day-to-day basis, with management support from Rivas and Aksoy. We reduce costs by recruiting an extra

Justification of Costs
RA only for WP1 and the first wave of WP3. Both PDRAs will support the project team on analyses, output development and events. Each will produce 1-2 articles and deliver 1 UK conference talk.<br><br>HEI Indirect Costs – Overall Total £310,265.50<br>UCL Sub Total £289,273.28.<br>KCL Sub Total £5,558.10<br>QMUL Sub Total £10,308.12.<br>Brighton and Sussex Medical School Sub Total £5,126.00.<br><br>Commercial and Other Partnership Organisation Costs - Overall Total £90,445.20<br>Bromley by Bow Community Centre - Consultant Catherine Godward (£51,505.20): hosts a GP practice, church, children's centre, community facilities for 2,000 people weekly as UK's first Healthy Living Centre. It supports community members to undertake health and wellbeing research, providing London co-researchers for interviews and workshops. Member of the ActEarly consortium London collaboratory. Citizen Scientist 1, day rate £175 x 34 days (£5950); Citizen Sci 2, day rate £175 x 34 (£5950);Res & Eval Coordinator, day rate £325 x 32.5 (£10562.50); Res & Eval Mgr, day rate £450 x 15 (£6750); Dir of Knowledge & Innovation, day rate £600 x 3 (£1800); Knowledge Share Exchange Mgr, day rate £450 x 6 (£2700); Other cost: mobile phone citizen scis @£30 pm - £180; Finance (£5083.88); End of project evaluation (£1694.63); Translation up to 9 interviews (£2250); VAT (£8584.20)<br><br>The Migrant Solidarity Group (£240.00) will support recruitment of participants not easily reached online; it comprises frontline clinicians with an interest in migrant health.<br>Consultant Prof Mohammed Abou-Saleh (£18,000.00) - expertise and influence in the WHO (Biological Psychiatry), Syrian Association for Mental Health (Refugees), Helen Bamber Foundation for assessment of refugees and asylum seekers and the Refugee Council, London.<br>The Ultimate You (£2,700.00 - £450 per hour x 5 hours plus VAT) - PPI member Gaulbert will modify her existing course to train multidisciplinary clinicians how to utilise and incorporate our findings into practice, as an output. Tough Cookie - Mental Resilience programme for the community (£18,000.00 - 5 x ½-days at £1,500 per ½-day;5 x training sessions at £1,500 per session plus VAT), costs to modify and test it as an output.<br><br>Travel, Subsistence and Dissemination – Overall Total £9,455.14<br>Sub Theme (steering and advisory group external members) Sub Total £0: Costs assume meetings all online.<br>Sub Theme (researcher travel to sites) Sub Total £2,076.30: One TBC PDRA to visit the 5 sites 3 times each for site support; we assume this will be possible. Costed as £75 per trip to Canterbury, £19.10 per visit within London, and £150 per trip to the other 3 sites, plus subsistence for one day (£37) per visit.<br><br>Sub Theme (international conference attendance) Sub Total £1,255.00: One person to attend Conference for International Health, University of Bergen, with return flights and internal travel to Bergen from London (£350), and accommodation for 4 nights at £180 a night. Subsistence £37 a day for meals for 4 days. To reach global migrant health experts.<br><br>Sub Theme (UK conference attendance) Sub Total £1,226.00: BSA Medical Society Conference, 1 person and SAPC in year 1 require return train travel to Newcastle and UCLAN from London costed at £250 respectively, with accommodation for 1 and 3 nights, respectively, at £180 per night. Additional subsistence, £37 a day, for 2 and 4 days respectively. For dissemination to UK academics and practitioners.<br>Sub Theme (1 international and 2 UK conference fees) Sub Total £1,550.

Justification of Costs
Dissemination Event Costs: Venue hire, Catering. AV hire (100 attendees) Sub Total £3,070.00.<br>Dissemination printing costs e.g. newsletters, posters Sub Total £277.84.<br><br>Patient & Public Involvement – Overall Total £72,759.68<br>Attendance time for 5 advisors (additional to our 2 lay co-Is) at the co-production workshops (£45,750), and final events (£320), community co-researcher time (£2,250) and participant remuneration for workshops and interviews (£10,260), all at INVOLVE-recommended rates.<br>Ms Jenny Camaradou (PPI) 18.5% FTE for 18 months (£9,150.00) 61 days at £150 per day (INVOLVE-recommended rate); Dr Sarabajaya Kumar (PPI/UCL) – 5% FTE for 18 months (Sub Total £5,029.68).<br><br>Other Direct Costs – Overall Total £25,155.80<br>Audio Recorders (5 units) Sub Total £950.00.<br>Disposable cameras (100 units) for Photovoice Sub Total £931.80.<br>Portable storage (1 TB unit) for field sites secure data transfer Sub Total £60.00<br>Transcription costs of recorded interviews and workshops Sub Total £19,494.00.<br>Website Design Costs Sub Total £3,720.00.

DETAILED BUDGET SUMMARY

Research Costs Required from Funder

	Direct costs	Indirect costs	Total costs	% Costs paid by NIHR	Amount requested
Total Higher Education Institution Costs	381,987.45	310,265.50	692,252.95	80%	553,802.36
Total NHS Costs	48,242.40		48,242.40	100%	48,242.40
Total Commercial Costs		90,445.20	90,445.20	100%	90,445.20
Total Other Partnership Organisation Costs			.00	100%	.00

**Total Research Costs Required from Funder**	**£692,489.96**

Total NHS Support & Treatment Costs / (Savings)

	Total costs
NHS Support Costs Required from Networks	£
NHS Treatment Costs Requested from the NHS	£.00
Total NHS Support & Treatment Costs / (Savings)	£.00

Total Funding Required

	Amount requested
Total Research Costs Requested (not including NHS Support & Treatment Costs)	£692,489.96
NHS Support & Treatment Costs / (Savings)	£.00
**Total Cost of Research (Research + NHS costs)**	**£692,489.96**

DETAILED BUDGET BREAKDOWN

Posts & Salaries – Summary

	Year 1	Year 2	Year 3	Year 4	Year 5	Year 6	Year 7	Year 8	Year 9	Year 10
Total Higher Education Institution Costs	£154,656.10	£65,037.37								
Total NHS Research Costs	£32,086.96	£16,155.44								
Total Commercial Costs										
Total Other Partnership Organisation Costs										
Total NIHR Awarded Costs	£186,743.06	£81,192.81								

Finance costs (summary)

Travel, Subsistence & Dissemination Costs - Summary

	Total Costs
**Total Higher Education Institution Costs**	£7,564.11
**Total NHS Costs**	£0.00
**Total Commercial Costs**	£0.00

**Total Other Partnership Organisation Costs**	£0.00
**Total NIHR Awarded Costs**	£7,564.11

Equipment - Summary

	Total Costs
**Total Higher Education Institution Costs**	£0.00
**Total NHS Costs**	£0.00
**Total Commercial Costs**	£0.00
**Total Other Partnership Organisation Costs**	£0.00

Consumables - Summary

	Total Costs
**Total Higher Education Institution Costs**	£0.00
**Total NHS Costs**	£0.00
**Total Commercial Costs**	£0.00
**Total Other Partnership Organisation Costs**	£0.00

Patient and Public Involvement Costs - Summary

	Total Costs
**Total Higher Education Institution Costs**	£58,207.74
**Total NHS Costs**	£0.00
**Total Commercial Costs**	£0.00
**Total Other Partnership Organisation Costs**	£0.00
**Total NIHR Awarded Costs**	£58,207.74

Other Direct Costs - Summary

	Total Costs
**Total Higher Education Institution Costs**	£20,124.64
**Total NHS Costs**	£0.00
**Total Commercial Costs**	£0.00
**Total Other Partnership Organisation Costs**	£0.00
**Total NIHR Awarded Costs**	£20,124.64

HEI Indirect Costs – Summary

Estates Costs

Description	Year 1	Year 2	Year 3	Year 4	Year 5	Year 6	Year 7	Year 8	Year 9	Year 10
UCL<br>Total: £34,703.46	£25,037.68	£9,665.78								
Kings College London<br>Total: £1,448.48	£965.65	£482.83								
Queen Mary University of London<br>Total: £2,158.38	£1,438.92	£719.46								
University of Sussex<br>Total: £1,185.00	£790.00	£395.00								
Total:	£28,232.25	£11,263.07								
Total NIHR Awarded Costs:	£22,585.80	£9,010.46								

Other Indirect Costs

Description	Year 1	Year 2	Year 3	Year 4	Year 5	Year 6	Year 7	Year 8	Year 9	Year 10
UCL<br>Total: £254,569.81	£183,665.79	£70,904.02								
Kings College London<br>Total: £4,109.63	£2,739.75	£1,369.88								
Queen Mary University of London<br>Total: £8,149.74	£5,433.16	£2,716.58								
University of Sussex<br>Total: £3,941.00	£2,627.33	£1,313.67								
Total:	£194,466.03	£76,304.15								

Total NIHR Awarded Costs:	£155,572.82	£61,043.32								

Commercial and Other Partnership Organisation Costs

Description	Year 1	Year 2	Year 3	Year 4	Year 5	Year 6	Year 7	Year 8	Year 9	Year 10

Bromley by Bow Community Centre: Consultant; Catherine Godward (£42,921 plus VAT = £51,505.20) Citizen Scientist 1, day rate £175 x 34 days (£5950); Citizen Sci 2, £175 x 34 (£5950);Res & Eval Coordinator, £325 x 32.5 (£10562.50); Res & Eval Mgr, £450 x 15 (£6750); Dir of Knowledge & Innovation, £600 x 3 (£1800); Knowledge Share Exchange Mgr, £450 x 6 (£2700);Other cost - mobile phone citizen scis @£30 pm (£180); Finance (£5083.88);End of project eval(£1694.63); Translation(£2250);VAT(£8584.20)<br>Total: £51,505.20	£34,336.80	£17,168.40								

The Ultimate You - Consultant Sheren Gaulbert (£450 per hour x 5 hours - £2,250 plus VAT = £2,700)<br>Total: £2,700.00	£1,798.36	£901.64									
Tough Cookie - Consultant Mental Resilience (5 x ½-days at £1,500 per ½-day; 5 x training sessions at £1,500 per session = £15,000 plus VAT = £18,000)<br>Total: £18,000.00	£11,989.05	£6,010.95									
Migrant Solidarity Group (MSG) - Consultancy Jonathan Broad (£100 per hour plus VAT = £120) x 2<br>Total: £240.00	£159.85	£80.15									
Psychiatric Consulting UK Ltd - Consultant Prof Mohammed Abou-Saleh (£100 per hour plus VAT = £120) x 180 hours<br>Total: £18,000.00	£11,989.05	£6,010.95									
Total:	£60,273.11	£30,172.09									
Total NIHR Awarded Costs:	£60,273.11	£30,172.09									

Finance costs (detail)

Posts and Salaries – Details

Lead or Co-Applicant

Lead or Co-Applicant Information	Further Details	Current Basic Salary	Geographical Weighting	Superann. And Nat. Insurance	Current Annual Costs
Aksoy, Ozan<br>UCL<br>Co-Applicant<br>Grade: UCLGR9<br>Inc. Date:		£55,210.00	£3,211.00	£20,235.00	£78,656.00
Nasim, Bilal<br>UCL<br>Co-Applicant<br>Grade: UCLGR8<br>Inc. Date:		£42,370.00	£3,211.00	£15,521.00	£61,102.00
Dobson, Ruth<br>Queen Mary University of London<br>Co-Applicant<br>Grade:<br>Inc. Date:		£90,275.00	£1,470.00	£33,765.00	£125,510.00
Thomson, Alison<br>Queen Mary University of London<br>Co-Applicant<br>Grade:<br>Inc. Date:		£49,884.00		£17,837.00	£67,721.00

Lead or Co-Applicant Information	Further Details	Current Basic Salary	Geographical Weighting	Superann. And Nat. Insurance	Current Annual Costs
Eccles, Jessica<br>University of Susses<br>Co-Applicant<br>Grade:<br>Inc. Date:		£98,481.00		£33,156.00	£131,637.00
Goff, Louise<br>Kings College London<br>Co-Applicant<br>Grade: R New/05<br>Inc. Date:		£60,905.00	£3,500.00	£22,982.00	£87,387.00
Redclift, Victoria<br>UCL<br>Co-Applicant<br>Grade: UCLGR9<br>Inc. Date:		£56,862.00	£3,211.00	£20,841.00	£80,914.00
Dickerson, Josie<br>Bradford Teaching Hospitals NHS Foundation Trust<br>Co-Applicant<br>Grade: XN1001<br>Inc. Date:		£63,751.00	£0.00	£21,138.00	£84,889.00
Ball, Elizabeth<br>Barts Health NHS Trust<br>Co-Applicant<br>Grade: YM72<br>Inc. Date:		£98,477.00	£2,162.00	£34,084.00	£134,723.00

Lead or Co-Applicant Information	Further Details	Current Basic Salary	Geographical Weighting	Superann. And Nat. Insurance	Current Annual Costs
Saravanan, Vadivelu<br>Gateshead Health NHS Foundation Trust<br>Co-Applicant<br>Grade:<br>Inc. Date:		£0.00	£0.00	£0.00	£0.00
Rivas, Carol<br>UCL<br>Lead Investigator<br>Grade: UCLGR9<br>Inc. Date:		£60,315.00	£3,211.00	£22,109.00	£85,635.00

Research Staff

Research Staff Information	Further Details	Current Basic Salary	Geographical Weighting	Superann. And Nat. Insurance	Current Annual Costs
TBA 1 (UCL)<br>UCL<br>Research Assistant (Qualitative)<br>Grade: UCLGR6B<br>Inc. Date:		£31,598.00	£3,211.00	£11,424.00	£46,233.00
TBA 2 (UCL)<br>UCL<br>Research Fellow (Qualitative)<br>Grade: UCLGR7<br>Inc. Date:		£33,470.00	£3,211.00	£12,254.00	£48,935.00

Research Staff Information	Further Details	Current Basic Salary	Geographical Weighting	Superann. And Nat. Insurance	Current Annual Costs
TBA 3 (UCL)<br>UCL<br>Researcher Fellow (Quantitative)<br>Grade: UCLGR7<br>Inc. Date:		£33,470.00	£3,211.00	£12,254.00	£48,935.00

Shared Staff

Shared Staff Information	Further Details	Current Basic Salary	Geographical Weighting	Superann. And Nat. Insurance	Current Annual Costs

Other Staff

Other Staff Information	Further Details	Current Basic Salary	Geographical Weighting	Superann. And Nat. Insurance	Current Annual Costs
Newsham, Alex<br>Bradford Teaching Hospitals NHS Foundation Trust<br>Data Management<br>Grade : XN0802<br>Inc. Date:		£45,753.00	£0.00	£14,828.00	£60,581.00
TBA (BTHFT)<br>Bradford Teaching Hospitals NHS Foundation Trust<br>Project Manager<br>Grade : XN0705<br>Inc. Date:		£40,894.00	£0.00	£13,125.00	£54,019.00

Annual Costs of Posts and Salaries

Lead or Co-Applicant

Information	Year 1	Year 2	Year 3	Year 4	Year 5	Year 6	Year 7	Year 8	Year 9	Year 10
**Aksoy, Ozan**<br>HEI<br>**Total: £23,823.32**	£15,731.25<br>20.00% FTE<br>12 Months	£8,092.07<br>20.00% FTE<br>6 Months								
**Ball, Elizabeth**<br>NHS<br>**Total: £10,104.23**	£6,736.15<br>5.00% FTE<br>12 Months	£3,368.08<br>5.00% FTE<br>6 Months								
**Dickerson, Josie**<br>NHS<br>**Total: £12,733.41**	£8,488.94<br>10.00% FTE<br>12 Months	£4,244.47<br>10.00% FTE<br>6 Months								
**Dobson, Ruth**<br>HEI<br>**Total: £10,118.89**	£6,745.93<br>5.00% FTE<br>12 Months	£3,372.96<br>5.00% FTE<br>6 Months								
**Eccles, Jessica**<br>HEI<br>**Total: £9,074.00**	£6,049.33<br>5.00% FTE<br>12 Months	£3,024.67<br>5.00% FTE<br>6 Months								
**Goff, Louise**<br>HEI<br>**Total: £6,785.84**	£4,477.22<br>5.00% FTE<br>12 Months	£2,308.62<br>5.00% FTE<br>6 Months								
**Nasim, Bilal**<br>HEI<br>**Total: £4,626.29**	£3,055.10<br>5.00% FTE<br>12 Months	£1,571.19<br>5.00% FTE<br>6 Months								
**Redclift, Victoria**<br>HEI<br>**Total: £6,126.84**	£4,045.71<br>5.00% FTE<br>12 Months	£2,081.13<br>5.00% FTE<br>6 Months								
**Saravanan, Vadivelu**<br>NHS<br>**Total: £0.00**	£0.00<br>5.00% FTE<br>12 Months	£0.00<br>5.00% FTE<br>6 Months								
**Thomson, Alison**<br>HEI<br>**Total: £5,142.00**	£3,428.00<br>5.00% FTE<br>12 Months	£1,714.00<br>5.00% FTE<br>6 Months								

**Rivas, Carol**<br>HEI<br>**Total: £25,937.54**	£17,126.99<br>20.00% FTE<br>12 Months	£8,810.55<br>20.00% FTE<br>6 Months								
**Total:**	£75,884.62	£38,587.74								
**Total NIHR Awarded Costs:**	£63,752.71	£32,392.70								

Research Staff

Information	Year 1	Year 2	Year 3	Year 4	Year 5	Year 6	Year 7	Year 8	Year 9	Year 10
**TBA 1 (UCL)**<br>HEI<br>**Total: £34,790.81**	£34,790.81<br>100.00% FTE<br>9 Months	£0.00<br>0.00% FTE<br>0 Months								
**TBA 2 (UCL)**<br>HEI<br>**Total: £74,095.65**	£48,934.89<br>100.00% FTE<br>12 Months	£25,160.76<br>100.00% FTE<br>6 Months								
**TBA 3 (UCL)**<br>HEI<br>**Total: £74,095.65**	£48,934.89<br>100.00% FTE<br>12 Months	£25,160.76<br>100.00% FTE<br>6 Months								
**Total:**	£132,660.59	£50,321.52								
**Total NIHR Awarded Costs:**	£106,128.47	£40,257.22								

Shared Staff

Information	Year 1	Year 2	Year 3	Year 4	Year 5	Year 6	Year 7	Year 8	Year 9	Year 10

Other Staff

Information	Year 1	Year 2	Year 3	Year 4	Year 5	Year 6	Year 7	Year 8	Year 9	Year 10
**Newsham, Alex**<br>NHS<br>**Total: £9,087.19**	£6,058.13<br>10.00% FTE<br>12 Months	£3,029.06<br>10.00% FTE<br>6 Months								
**TBA (BTHFT)**<br>NHS<br>**Total: £16,317.57**	£10,803.74<br>20.00% FTE<br>12 Months	£5,513.83<br>20.00% FTE<br>6 Months								
**Total:**	£16,861.87	£8,542.89								
**Total NIHR Awarded Costs:**	£16,861.87	£8,542.89								

Travel, Subsistence & Dissemination Costs – Details

Journey Costs

Description	Year 1	Year 2	Year 3	Year 4	Year 5	Year 6	Year 7	Year 8	Year 9	Year 10
Researcher travel to sites: Travel & Subsistence (5 sites, 9 trips, 1 person)<br>HEI<br>Total: £2,076.30	£1,382.94	£693.36								

EU Conference x 1: Travel, Accommodation, Subsistence (1 person, 4 nights)<br>HEI<br>Total: £1,255.00	£0.00	£1,255.00								
UK Conferences x 2: Travel, Accommodation, Subsistence (1 person, 4 nights)<br>HEI<br>Total: £1,226.00	£1,226.00	£0.00								
Total:	£2,608.94	£1,948.36								
Total NIHR Awarded Costs:	£2,087.15	£1,558.69								

Subsistence

Description	Year 1	Year 2	Year 3	Year 4	Year 5	Year 6	Year 7	Year 8	Year 9	Year 10

Dissemination Costs - Conference

Description	Year 1	Year 2	Year 3	Year 4	Year 5	Year 6	Year 7	Year 8	Year 9	Year 10

Conference Registration Costs: UK x 2 and EU x 1 (1 person per conference)<br>HEI<br>Total: £1,550.00	£1,000.00	£550.00								
Total:	£1,000.00	£550.00								
Total NIHR Awarded Costs:	£800.00	£440.00								

Dissemination Costs - Open Access

Description	Year 1	Year 2	Year 3	Year 4	Year 5	Year 6	Year 7	Year 8	Year 9	Year 10

Dissemination Costs - Other

Description	Year 1	Year 2	Year 3	Year 4	Year 5	Year 6	Year 7	Year 8	Year 9	Year 10
Dissemination Event Costs: Venue Hire, Catering, AV hire (100 attendees)<br>HEI<br>Total: £3,070.00	£0.00	£3,070.00								
Printing Costs for Dissemination Event<br>HEI<br>Total: £277.84	£0.00	£277.84								
Total:	£0.00	£3,347.84								

Total NIHR Awarded Costs:	£0.00	£2,678.27								

Equipment – Details

Description	Year 1	Year 2	Year 3	Year 4	Year 5	Year 6	Year 7	Year 8	Year 9	Year 10

Consumables – Details

Description	Year 1	Year 2	Year 3	Year 4	Year 5	Year 6	Year 7	Year 8	Year 9	Year 10

Patient and Public Involvement Costs – Details

Description	Year 1	Year 2	Year 3	Year 4	Year 5	Year 6	Year 7	Year 8	Year 9	Year 10
PPI Costs: Community co-researchers: £30 vouchers (75 people)<br>HEI<br>Total: £2,250.00	£1,498.63	£751.37								
PPI Costs: Co-Production Workshops: Patient Advisors x 5 x 61 days each @ £150 per day/ NIHR INVOLVE rates)<br>HEI<br>Total: £45,750.00	£30,466.61	£15,283.39								

PPI Costs: Interviews and advisory groups: £20 vouchers for interviews/ workshops (513 people)<br>HEI<br>Total: £10,260.00	£6,833.76	£3,426.24									
PPI Costs: Co-Production Workshops: £20 vouchers (16 people)<br>HEI<br>Total: £320.00	£213.14	£106.86									
PPI Cost: Salary Costs - Sarabajaya Kumar, UCL, PPI Co-Applicant (5% FTE)<br>HEI<br>Total: £5,029.68	£3,321.39	£1,708.29									
PPI Cost: Jenny Camaradou, PPI Co-Applicant, Independent Patient Adviser - £150 per day (INVOLVE-recommended rate) x 61 days<br>HEI<br>Total: £9,150.00	£6,100.00	£3,050.00									
Total:	£48,433.53	£24,326.15									
Total NIHR Awarded Costs:	£38,746.82	£19,460.92									

Other Direct Costs – Details

Description	Year 1	Year 2	Year 3	Year 4	Year 5	Year 6	Year 7	Year 8	Year 9	Year 10
Audio Recorders x 5<br>HEI<br>Total: £950.00	£950.00	£0.00								
Disposable Cameras for Photovoice x 100<br>HEI<br>Total: £931.80	£931.80	£0.00								
Portable Storage x 1<br>HEI<br>Total: £60.00	£60.00	£0.00								
Transcription Costs: 285 hours of Interviews (14 day turnaround)<br>HEI<br>Total: £19,494.00	£12,984.14	£6,509.86								
Website Design<br>HEI<br>Total: £3,720.00	£3,720.00	£0.00								
Total:	£18,645.94	£6,509.86								
Total NIHR Awarded Costs:	£14,916.75	£5,207.89								

NHS Support and Treatment Costs

**Have you discussed and agreed these support costs with The Lead Network?**	No
**Have you discussed and agreed these treatment costs with The Lead Trust?**	No

Is the patient care being provided different from the usual treatment for the condition?	No

NHS Support Costs

Description	Year 1	Year 2	Year 3	Year 4	Year 5	Year 6	Year 7	Year 8	Year 9	Year 10

NHS Treatment Costs

Description	Year 1	Year 2	Year 3	Year 4	Year 5	Year 6	Year 7	Year 8	Year 9	Year 10

Usual Treatment Costs

Description	Year 1	Year 2	Year 3	Year 4	Year 5	Year 6	Year 7	Year 8	Year 9	Year 10

Excess Treatment Costs / (Savings)

Description	Year 1	Year 2	Year 3	Year 4	Year 5	Year 6	Year 7	Year 8	Year 9	Year 10

MANAGEMENT & GOVERNANCE

**Is Clinical Trial Authorisation required?**	No
**Does your project require ethics approval?**	Yes
**If yes, has ethics approval already been obtained?**	Yes

UPLOADS

The following pages contain the following uploads:

Upload Name
Cover Letter (Response to Feedback)
Detailed Research Plan (Tracked Changes)
Detailed Research Plan (Final Clean Version)
Flowchart
References
Additional Documentation

Thank you for the feedback which we have found very helpful in ensuring clarity and good impact. As a result of two changes, we have slightly increased the total by £5,061.60, that is from £687,428.36. to £692,489.96. We hope this is acceptable. We have highlighted below where the additional costs have arisen; the itemised costs below add up to more than the increase because they were slightly offset by a small decrease in Bromley-by-Bow's consultancy costs.

1. *Thank you for your positive comments.*

2. *Thank you for your positive comments.*

3. ***Methodological and theoretical underpinning:***

3.1. There is a very brief account of the data synthesis*:* A fuller account, added to p11 of the Final research plan, is as follows:

Synthesis will be results-based, with thematic tabulation derived from WP1-3 data analyses, with table columns for themes, rows giving quantitative and qualitative data. Some data will need to be transformed (quantified or qualitised) for tabulation e.g. network graphs. We will interrogate the tabulated data using anchor questions based on the **PerSPectif** framework (50) (informed e.g. by data convergence/divergence patterns). This will form the basis of guidance and recommendations outputs and will inform interventions. An example question (PerSPectif highlighted) might be: *"From the* ***per****spective of a first generation single woman aged 18-30 of African descent, with complex dietary needs, in a rural Yorkshire* ***s****etting, how does the* ***p****henomenon of community-based food banks, within an* ***e****nvironment of poor transport infrastructure and geographically remote facilities (a food desert)* ***c****ompare with online shopping during a* ***t****ime of national lockdown in relation to* ***f****indings on their perspectives and key study outcomes*". Specific questions will be based on data findings, e.g. intersectional variables and outcomes. Disaggregation will be built in where possible and useful, though in a complex systems approach this may sometimes be misleading due to system interdependencies. Systems diagrams, logic models and other approaches will be used where appropriate. This evidence to decision approach supports our aim to report explanatory findings with practical application.

3.2. ***Multiple group analysis and developmental cascade models and Credibility, internal validity, transparency and reproducibility***

Multiple Group Analysis (MGA) is a technique within the Structural Equation Modelling (SEM) framework. In MGA, the same structural model is fitted to data from multiple groups, in this case different minority and disability status groups, whereby parameters of the model are allowed to differ across these groups. MGA then allows to test cross-group differences of model parameters (e.g. it will reveal if social support has the same effect on mental wellbeing or coping for the majority and minority groups). MGA is a flexible method to test *interactions* (in the statistical sense) between categorical variables (e.g. minority and disability status) and other categorical or continuous variables, including *latent* variables (e.g. social support, networks, access to resources). MGA is now a standard technique with a well-established protocol (see e.g. Kline 2016 ch16). We will follow these protocols. Developmental cascade models will also be analysed within the SEM framework, in the form of Latent Growth Modelling. In these models (latent) variables "affect" contemporaneous and future measures of latent variables. Such models will reveal if, for example, initial lack of support and mental ill health compound over time, influencing subsequent issues such as reduced social network resources or control of life.

To ensure transparency and reproducibility in all quantitative analyses, analysis code (Stata, R, and Mplus syntax) used for the Multiple Group Analysis and Latent Growth Models will be documented on GitHub and Open Science Framework. This will also serve as an external validity check as these encourage dialogue. For the qualitative data we will ensure credibility/internal and external validity through approaches such as exemplar data extracts, data collection triangulation, and team and participant workshop data discussions, transparency through a clear audit trial, and reproducibility through thick descriptions of context and analysis. Anonymised data will be archived for secondary analyses.

Reference: Kline, R. B. (2016). *Methodology in the social sciences. Principles and practice of structural equation modeling (4th ed.).* Guilford Press.

3.3 Discourse and narrative analysis

The description has been expanded/clarified on p10 of the Final research plan with the following:

The discourse analysis will examine the linguistic and discursive resources participants use to link social practices (e.g. ableism, racism) to their situation and with the narrative analysis of participant 'stories' will further contextualise findings on wellbeing and coping, social identity and networks, attitude to and relationship with services - important for services to build trust. Black peoples' mistrust of the COVID vaccine is relevant here for example. These analyses will reveal potential mechanisms for behavioural responses to help us develop our evidence to decision interrogation (WP4).

3.4 *The approach to the qualitative data collection and analysis requires clarification.*

Apologies, we are not clear what is required here but will try to respond, and will be happy to further clarify again if needed. The methods described are standard approaches, thus semi-structured interviews (including tools to elicit in-depth information – network/local access map/photo elicitation methods), followed by interactive online workshops. We also have 5 stakeholder 2-hour co-create workshops with different participants and 15 x 1-hour key informant interviews.

The main analysis would be thematic, involving a keyword approach to help us develop a coding framework for Framework Analysis. This approach has been chosen to enable rapid analysis of a large amount of data, to so that basic findings can be disseminated as soon as possible. Discourse and narrative analysis will enable more in-depth work but take longer; thus we will achieve both a birds eye view and more granular analysis. We have explained the discourse and narrative analysis in our response 3.3. We would need a little more guidance to respond more fully here on any other aspects.

3.5. Assignment of people to interview cells

a) *Accurate reflection of their health conditions (as self-reported):* We will sample according to formal diagnoses where possible (we will ask this at recruitment stage) but also include self-reported diagnoses to ensure we capture the full range of impairments. We are sampling by impairment so this should be an accurate reflection of these, rather than health conditions per se. This is in alignment with existing national and international surveys, which also take this approach of sampling on impairment not condition (see e.g. https://gss.civilservice.gov.uk/policy-store/impairment/). This policy-oriented approach adheres to the social model of disability (see for example Rivas, Tomomatsu and Gough, Editorial, *Evidence and Policy,* May 2021). In practice, some conditions such as diabetes will likely be clinically diagnosed, while others such as endometriosis or chronic fatigue tend to be self-diagnosed years before formal diagnosis so the main impact area that this relates to is probably fatigue. But it is important not to exclude these conditions. We will ask potential participants to describe their impairments at the time of recruitment to ensure rich data.

b) Number per cell allows for intersectionalities to be confidently identified and analysed: Intersectionality studies assume populations are homogeneous enough along some dimensions in order to be able to explore heterogeneity in others. The alternative, taking account of all relevant intersectional combinations, would result in an impractically large qualitative sample. Moreover, focusing on differences in categories or identities might conceal commonalities and lead to the 'othering' of some participants. Typically, therefore, researchers using an intersectional approach may frame a health issue under study as a shared commonality across categories of race, gender, sexual orientation, and so on. At the analysis stage the focus may switch to other commonalities such as shared barriers or facilitators to health resources. Taking this approach means that effectively we are considering 30 participants per ethnic grouping, or if you cut the sample differently, 30 per condition impact type. Using the sampling frame cell approach is therefore designed to ensure variation within these groups of 30. Hence the sampling frame is simply a tool to organize current understanding, that can help us to tackle the deeper issues, and the number per cell is the product of a more formal and systematic way of specifying the sampling frame than is normally used.

We have added to the sampling frame text on p13: Our sampling frame follows an intersectional studies approach that allows us to consider and compare assumed homogeneity across condition effects irrespective of ethnicity, and across ethnicity irrespective of condition, as a tool to tease out intersectional factors and heterogeneity. At analysis the focus may switch to other commonalities such as shared barriers or facilitators to health and social care resources.

References:
Abrams, J. A., Tabaac, A., Jung, S., & Else-Quest, N. M. (2020). Considerations for employing intersectionality in qualitative health research. *Social Science and Medicine, 258,* 113138.
Shim, J. K., Darling, K. W., Lappe, M. D., Thomson, L. K., Lee, S. S., Hiatt, R. A., & Ackerman, S. L. (2014). Homogeneity and heterogeneity as situational properties: producing--and moving beyond?--race in post-genomic science. *Social studies of science*, *44*(4), 579–599. https://doi.org/10.1177/0306312714531522

3.6. Feasibility work on the social network analysis

The data collection on Social Networks will take place in two forms: the first is in the large-scale survey, the second as part of the qualitative interviews. The nature of social network measurement will differ in these two versions. In the survey, we will use the standard ego-centred network items that use name generators, name interpreters for a limited number of "alters" in "ego"s (the respondent's) network, and the standard social network questions. Such social network components have been incorporated in standard large-scale surveys (e.g. General Social Survey or Understanding Society). We thus do not see any threats to feasibility of the social networks component of the large-scale survey, given that this is a standard approach which has already effectively been tested in CLS surveys, with good response. The social network component in the qualitative interviews (target n = 210) is more involved. It will include more detailed questions about the nature of support networks around "ego", such as on the features of the relationship between the "alters" and "ego" as well as between the "alters". To facilitate network data collection process, we will use tailored software (i.e. Network Canvas https://www.networkcanvas.com/). We will pilot the Network Canvas module before fielding it to check for feasibility. We believe social support network analyses are essential in understanding the effects of the pandemic on our participants, and that collecting good quality data on social networks during the pandemic is very important. Should the Network Canvas module prove to be unfeasible we will still be able to ascertain information from simple questions, but this provides a framework to probe for all important aspects.

4.How is English language fluency determined and when would translators be involved?

Following the recent experiences of co-applicant Dickerson with the Born in Bradford study she leads, we have simplified this process for transparency for all. Our starting design is to only include people who can converse sufficiently well in English to be able to reply to interview questions with 'thick description', that is, answers that provide relevant data. Our plan to complete interviews in English will be made clear to participants. English language ability will be self-assessed by participants who, having been informed that interviews will be completed in English, will be asked whether they feel able to take part, which is their choice. This is a well formulated process in Born in Bradford and works well (see e.g. references to point 11). However, there are times where a participant is happy to interview in English, but feels more comfortable completing an interview in their home language. Where this is the case, and a researcher fluent in that language is available, this will be arranged. Bromley by Bow has a pool of translators, and we have added costs for these, in case needed, to the application – an additional £2,250 is therefore requested if this is acceptable.

Our consent documents and patient information sheets have been developed with PPI input and are written in simple English, and consent and the study details will also be explained verbally at the time consent is confirmed prior to data collection, with the opportunity for questions, to ensure fully informed consent.

The text on p14-15 has been amended to improve clarity as follows:

Potential participants will be informed that interviews will be in English, and it is their choice as to whether they feel able to take part. This is a well formulated and effective process in Born in Bradford. Where a participant is happy to interview in English, but feels more comfortable doing so in their home language, if a researcher fluent in that language is available this will be arranged.

5. It was not clear how the theoretical underpinning informed the data collection and analysis.

To further expand on the description of the two theories on p6, the socioecological theory provides a structure for considering the multidirectional interplay of the different levels. The CFIR has been designed to consider the effect on implementation of multiple interconnecting systems and contexts and is therefore highly suited to complex 'real world' interventions.

We have added to p6 Theoretical framework: These theories will inform interview topic guides, and survey questions for topics 3, 4, 7 and 8 in the next section [Access issues, Social networks,

Local/regional differences, Future policy implementation]. In our analysis they will inform health and social care mapping and implementation considerations across multiple levels for integrated care.

6. ***Clarification should be provided on who is the qualitative lead in the project, and who is accountable for the qualitative analysis.***

This is the PI in both cases, now fully clarified in the PI description of the main form.

7. ***More information is requested on the amount of training for members of the community to perform the data collection, and the feasibility of this approach.***

We are drawing on the Bromley-by-Bow model and considerable expertise for this; they have frequently run training covering community participation and communication and listening skills, for those with lived experience of the topic (see https://www.bbbc.org.uk/insights/). Training over 2 days will cover:

- explanation of each stage of research from design to report;
- project aims, values and the community research team;
- reflexivity and the role of the researcher;
- processes of data collection that co-researchers will undertake for the study;
- "the human touch" (holding good conversations, active listening skills, exploring empathy);
- ethical practices based on "principles of good research" (remember the purpose, keep everyone safe, act in service, respect people's information and be transparent);
- ways for the co-researcher to be supported.

At the beginning of the ActEarly project, the Bromley-by-Bow team created measurable "ethical commitments" relevant to the principles of good research. These were then reviewed periodically, particularly during data collection (e.g. anticipating ethical dilemmas and then discussing experience after the interviews). We will draw on this learning.

We will have learning 'sets' or communities of practice, with reflective practice in regular team meetings (including weekly - challenges and opportunities - and quarterly and after major milestones, which involves a fuller reflective cycle). We will include role playing (interview practice) before the first data collection. We will also make it a habit to encourage co-researchers tp complete reflection sheets and live debriefs as needed, to process the experience without overburdening them.

8. ***A description of the sampling should be included in the plain English summary.***

Thank you, this is included in a separate document with tracked changes and is also modified on the main form. Some of the changes had to be made to keep the abstract within the character count for the main form. In full this lay abstract now therefore reads:

Many people from Black, Asian and Minority Ethnic groups (BAME), especially those with underlying (chronic) conditions/disabilities, face barriers to accessing networks of appropriate support, health and social care or vital 'resources', such as medicine and food. Around 50% lived in poverty in 2019; the pandemic has worsened their plight, highlighting the need for these barriers to be removed. To determine where and how best to intervene to ensure this, we need to understand the problems - and successes - these groups have experienced, especially as health and social care tries to return to normal. We also need to understand what affects their vaccine uptake and if pandemic-related service changes that are retained further exclude them. Our approach is 'intersectional'. This means we recognise everyone is affected differently by the pandemic, according to the intersection (interplay) of factors such as ethnicity, citizenship, age, gender, their work, and health or disability.

To understand this complex situation, we will survey 4,000 UK BAME people and for contrast 1000 white British, 3 times over 15 months. We will compare their health, social networks (who they have contact with) and how these help or hinder them, ways they cope with pandemic changes and associated access to support, care and resources. We will consider how intersectional factors affect this.

After Survey 1 we will interview 210 more people in 5 diverse sites in England about the same topics, probing for coping strategies and ideas to inform health and social care policy and practice. Interviewees will also describe their networks using special brief questionnaires, photos and maps. We use remote working. We will find people for the study via social media, NHS clinics, charities, special patient and migrant groups, our own networks, and large databases of adults interested in health research across the UK.

We will focus on migrants from the Middle East, India, Pakistan, Poland or Africa, or whose parents were born there, as the most likely to have problems (e.g. to have limited citizenship rights or to die from COVID-19). We will look at the impact of also having a chronic condition/disability including 'long covid'. Our PPI leads will help train local community members to do some of the research.

After surveys 2 and 3, interviewees will be invited to workshops to discuss findings and more recent changes. We will also review published and informal (e.g. blog) articles about pandemic BAME and disability experiences, and data from other complementary COVID-19 surveys. All our findings will be combined. Over the 18-month study we will hold 5 participatory sessions with members of different BAME communities and key informants working together to help analyse our data and co-design solutions to issues, pragmatically including ‘life hacks’ and service adaptations for rapid impact. At 16 months we will interview 15-25 key informants such as support staff and community leaders to help us put our work into immediate practice.

Findings and solutions will be shared as they emerge at each of the 3 data waves, for early benefit. We will report changes over time in experiences, outcomes and solutions and consider how to apply our work across the UK. We aim for immediate, readily implemented, relevant useful change in UK pandemic health and social care service delivery. We include training outputs, strong networks and Co-As with direct influence on policy and practice.

9. ***Greater social care involvement in the project is requested, which is introduced well, but not carried through the project sufficiently with current outcomes focused more around the health aspects of chronic conditions*.**

We have made sure that the social care outcomes are now carried through the study, in particular WP1 and WP2 research questions and outputs on pp7-9 of the Final research plan.

Social care teams are embedded in both Born in Bradford and Bromley-by-Bow and Bromley-by-Bow has costed in an activity that will take findings and recommendations to a series of London stakeholder specific sessions including social care teams, to build an aspirational action plan (now specified in the dissemination plan on p18). Stakeholders will include patients, researchers, academics, and voluntary and statutory sector staff. This model can be extended to other sites and nationally – we have added an additional £4250 in costs for the Bromley-by-Bow team to do so, if this is acceptable.

As an example of partners of Born in Bradford and Bromley-by-Bow including social care and extending beyond this, the ActEarly website lists: Arup, Centre for Cities, Department of Education, East London Genes Health, Health Education England, JRF, Local Government Association, National Housing Federation, NIHR CRN, NSPCC, NUPHA, Public Health England, RSA, TfL, West Yorkshire Police, Yorkshire Sport Foundation, Nesta, Tower Hamlets Borough, Bradford City Council.

10. Dissemination could be clearer and more ambitious.

We have edited pp 15-19 throughout (see tracked changes) and hope that we have clearly demonstrated more ambition. We believe some of the issues were simply around terms used in headings and subheadings, which reduced clarity so the changes are mostly moving sentences around and modifying subheadings (for example changing the subheading ‘Communication’ to ‘Dissemination’).

Ambitious award-winning work by co-A Thomson, a trained designer, is used internationally and we have now been explicit about this. Examples include Digesting Science (a game to educate children about MS) (Thomson et al, 2020) and tools to help patients measure the impact of multiple sclerosis on their lives (Thomson and Rivas, in prep). This recent article by co-applicant Thomson includes photographs of potential outputs from the Digesting Science project:

Reference: Thomson A, Dobson R, Baker D, Giovannoni G. Digesting science: Developing educational activities about multiple sclerosis, prevention and treatment to increase the confidence of affected families. Mult Scler Relat Disord. 2021 Jan;47:102624. doi: 10.1016/j.msard.2020.102624. Epub 2020 Nov 13. PMID: 33220567.

See also our response to point 9.

We have added on p15: The main platforms will be text and web-based. Recommendations and guidance will use an evidence to decision format (please see p11). More creative dissemination

and outputs may use art, 3-D work, performance art or other relevant approaches if our co-design participants recommend it; similar award-winning work by co-A Thomson, a trained designer, is used internationally. Some may be developed as seed projects from the original funding.

We have added on p18: All findings will be publicly available via our ***website in accessible forms*** for lay consumption using recommendations in the Patient Engagement Open forum (https://bit.ly/388SFr0) and by ***involving trusted community channels***, such as places of worship, trusted religious leaders, community champions - possibly tapping into the infrastructure developed from COVID vaccine rollout - and community groups, including collaborators Bromley-by-Bow. This aligns with Black community comments in a meeting about vaccine uptake and UK government 2021 vaccine hesitancy guidance prepared by the ethnicity sub-group of the Scientific Advisory Group for Emergencies (SAGE) (https://bit.ly/38GLt6D).

11. Vaccine uptake.

We agree that this is important, thank you, and have explicitly added this at various points throughout the Final research plan, pp 1,2,5,6,8,9,17,18. Initial work from Born in Bradford co-applicants complements this and was undertaken last year:

Bridget Lockyer, Shahid Islam, Aamnah Rahman, Josie Dickerson, Kate Pickett, TrevorSheldon, John Wright, Rosemary McEachan, Laura Sheard. Understanding Covid-19 misinformation and vaccine hesitancy in context: Findings from a qualitative study involving citizens in Bradford, UK. medRxiv 2020.12.22.20248259; doi:https://doi.org/10.1101/2020.12.22.20248259

Dickerson J, Lockyer B, Moss RH *et al.* COVID-19 vaccine hesitancy in an ethnically diverse community: descriptive findings from the Born in Bradford study [version 1; peer review: awaiting peer review]. *Wellcome Open Res* 2021, **6**:23 (https://doi.org/10.12688/wellcomeopenres.16576.1)

Secretariat feedback

We would like to clarify against the two studies stated, the following details.

1) Professor Ala's project will design and test an approach to delivering digital COVID-relevant messages to Black and South Asian groups through specific trusted communication channels, rather than determining how support itself might be improved, as we plan to do. While there is no overlap between projects, we should feed relevant findings to Professor Ala's team; these might help to inform their work and may also be important to dissemination of some of our findings. I will therefore contact Professor Ala to discuss how we might collaborate.
2) Dr Malpass' study is interesting, and can help to inform our own. The focus is slightly different so there is no direct overlap as our study considers pandemic-related access to services whilst this is a meta-ethnography specifically, that considers access more broadly. Unlike Dr Malpass' study, our main approach is to collect new data from surveys and qualitative methods. Given that Dr Malpass' study speaks to areas of interest of our study such as cultural intersectionalities and services access, and would be included in our WP1 review, I plan to contact Dr Malpass to consider what role she might have in our study, for example to be on our advisory team.

Finance

We have updated points 1 – 6 on the main form, please see the costs and JOR sections of the main form. We make further comments on specific points.

2. *the role/purpose of the 1TB portable storage*

This is encrypted and was for situations where we would wish to transfer data securely, for example where the lead researcher visits the co-researchers to transfer data from the equipment they are using.

4. recruitment costs

Removed

5.&6: costs moved.

IP feedback

- ***We are not using any Background IP that any third party owns or controls the rights to. We will suggest modifications to two existing training platforms but these will be modified and delivered by the IP holders.***

- ***Our contact for IP considerations is florin.buda@ucl.ac.uk with whom I have had initial discussions.***
- ***Reassurance of relevant permissions to access COVID-19 related UCL data (objective 4).***

In the Final research plan I have edited the name of the group from CLOSER, which is broader, to CLS which falls under the CLOSER umbrella but specifically curates the surveys mentioned in the Research plan, so that the below access statement can be linked directly to the Research plan. The Covid-19 surveys have been run embedded within sweeps of the national datasets that the CLS curates. But if needed we can obtain a letter of support from CLS to confirm access.

Official CLS access statement
Almost all the CLS data is available for free download at the UK Data Service, by means of an End User License, or occasionally a Special License.
https://cls.ucl.ac.uk/data-access-training/data-access/
https://ukdataservice.ac.uk/get-data/key-data/cohort-and-longitudinal-studies

NIHR132914. Carol Rivas UCL

CICADA-ME: Coronavirus Intersectionalities: Chronic Conditions and Disabilities and Migrants and other Ethnic minorities

SCIENTIFIC SUMMARY

Two groups that experience similar societal inequities (expanded by the COVID-19 pandemic), including in social and health care, are people with chronic conditions/disabilities (PwCD) and ethnic minorities. The worst affected are both ethnic minority AND with chronic conditions/disabilities, a common group, as COVID-19 mortality statistics show. There is a largely unmet expressed need to explore this combined group's pandemic experiences with new or worsening conditions/disabilities including post-Covid syndrome in relation to reduced services, inequalities, lifestyle changes or health neglect, and vaccine uptake. We aim to contribute and inform evidence-based formal and informal strategies, guidelines, recommendations and easily adopted interventions for pandemic-related and future health and social care policy and practice, to mitigate inequities and improve the experiences, health and wellbeing outcomes of minority ethnic groups at the intersection with chronic conditions/disabilities. To do so, we will develop a rich intersectional understanding of their mental and physical health, coping, access to resources, and informal and formal social and health care support experiences, and relevant assets and strengths, longitudinally over 18 months using mixed methods. Our 4 work packages involve a new UK survey in 3 waves, parallel qualitative insights, secondary analyses of other surveys, rapid review, and outputs for immediate use developed with participatory methods, with co-create workshops involving our PPI team and other stakeholders throughout. Our survey (n=5000) samples for 1st and 2nd generation community-dwelling minority ethnic groups and white British comparators, all with/without chronic conditions/disabilities, across the UK's 4 nations, to determine relationships between measured variables and their trajectories. After Survey Wave 1 we will interview 1st and 2nd generation ethnic minorities from Poland, India and Pakistan, sub-Saharan Africa, and the Middle East, and white British comparisons, all with and without chronic conditions/disabilities, about their pandemic experiences at 5 diverse sites in England, supporting transferability. Interviews (n=210) informed by survey analyses will include social network analysis, photovoice. We will train local lay people to help undertake these remotely; a transformative community migrant-majority research-active group will be our main London co-researcher. This group and a main co-applicant are members of an existing UKRI consortium and have undertaken complementary work at two of our sites, demonstrating the feasibility of our plans. At Waves 2 and 3 research workshops with interviewees will use video vignettes built from earlier study findings. Key informant interviews and co-create workshops will consider implementation. We will synthesise Keyword frequency analysis, Framework, discourse and narrative analyses, Latent Growth Modelling, Structural Equation Modelling, and social network analyses using tabulated evidence to decision methods, with interim findings reported at each wave for early delivery of benefits. Respondent and national UK demographic data will be compared for representativeness, and transferability explored at each stage. Data will be presented separately and combined for ethnic minorities and PwCD. We expect rapid impact from our strong networks and Co-A and collaborator existing strong pathways to influence in health and social policy and clinical practice.

Deleted: of health

Deleted: (shown protective for pandemic and non-pandemic health)…

Deleted: examining intersectional variations.

Deleted: will be

Deleted: and

Deleted: and compare findings across approaches using matrix mapping,…

BACKGROUND AND RATIONALE

The problem: The greater risks and challenges faced by two vulnerable groups during the COVID-19 pandemic, ethnic minorities and those with underlying health conditions/disabilities (1-8) are now well recognised. Although disabled people constitute 16% of the population, they represent 59% of all COVID deaths (9). Similarly, though 13% of the UK population, 33% of critically ill COVID-19 patients are from non-white ethnic groups (6,7). One reason is the intersection of minority ethnic status or chronic poor health or disability with other inequities (please see **Box 1**), which persisted before the pandemic and have widened because of it. Our particular interest is in improving pandemic and longer-term networks of support and access to care, services and resources for these vulnerable populations (1-5) to enhance vaccination, social, health and wellbeing outcomes.

The need to foreground intersections of chronic condition/disability AND ethnicity: Notably, the pandemic has highlighted how ethnic minority and poor health/disability statuses themselves intersect (1-5,8), with calls for research on this (e.g. BMJ) (10). **First**, having both chronic poor health/disability and ethnic minority status is associated with worse health than belonging to just one of these groups, even outside the pandemic, as noted for resettled refugees particularly (8,11). The emergence of post Covid syndrome, or long Covid (12,13), with a 5-week prevalence of 20% (14) has highlighted some of the issues. Anecdotal evidence suggests long Covid accounts may be more often ignored when made by people from ethnic minorities or who have a similar pre-existing disability such as complex

multisystemic conditions. **Second**, chronic conditions such as diabetes and cardiovascular disease are disproportionately common in some ethnic minority groups (15) - one reason for their increased risk of serious illness or death from COVID-19 (16). Considering mental health, the estimated 2% of the population who are recent refugees or undocumented migrants (11) had a considerably higher pre-pandemic prevalence of PTSD and depression than any other group (17) and minority ethnic groups report markedly poor pandemic mental health (18). To improve support and care for these vulnerable people, it is critical that we specifically consider the intersection of chronic conditions/disabilities (including long Covid) WITH ethnic minority status. This is the basis of our study. We will consider health and social care and support experiences across a range of combinations of chronic condition/disability and ethnicity. As **Box 1** shows, we cannot consider these intersections in isolation, though at the core of our study, so we also explore other categories of societal difference (e.g. age, gender) that interact with health status and ethnicity under institutional and structural conditions to create specific health outcomes and experiences (19). In particular we foreground citizenship status as influencing the support available to ethnic minorities, since many recent refugees and undocumented migrants will have 'no recourse' to welfare and housing support. Underpinning our study with intersectionality theory allows for complex nuanced insights into differences, while minimising the risks of a) essentialising some combinations as inherently problematic or b) considering the ethnicity/migrant experience as homogenous. In this application we refer to People with Chronic conditions or Disabilities (which we shorten to PwCD) AND ethnic minority status (encompassing a range of citizen states) as our focal group. But our study is designed to also be independently applicable to those with long Covid, chronic condition/disability, or ethnic minority status and in our outputs we will disaggregate these data.

The need for our particular study design: Critically many of the pandemic health and wellbeing challenges faced by our focal group can be mitigated by small adjustments to health and social care service policy and delivery, formal networks such as community health services and informal networks such as family and friends (11). Yet this is not done; the voices of PwCD or ethnic minorities rarely feature in pandemic planning (4), are not reflected in vaccine roll-out, and there is a remarkable lack of primary data. Public Health England has called for this to be addressed in the next stages of the pandemic through **participatory research** (20), which we will use alongside rapid review, surveys and interviews in a design involving strong PPI contribution throughout that makes our work relevant for ethnic minorities and PwCD separately and combined. Our **social network analysis** is key. An understanding of appropriate networks is vital to improving access to health/social care and support, resilience to stress and post-disaster recovery (19,21), and informing interventions based on health-related behaviours and health beliefs e.g. misinformation in the pandemic and vaccine uptake/hesitancy. We will explore how knowledge about network use may be harnessed to improve pandemic-related experience. Our study has a strong **practical focus**, important given our aim for immediate impact; it will use a **strength and assets-based mixed methods approach** to probe for resourcefulness and successful strategies/interventions used since the start of the pandemic. It includes consideration through the study of **new service delivery models** with continued use and advantage beyond the pandemic (e.g. telemedicine [22,23]). Importantly our study is **longitudinal**. Thus we will be able to explore significant relationships in the survey data we collect on mental and physical health, coping, access to resources, social and health care support, vaccine uptake and intersectional variables and also change in these over time and with varying pandemic contexts. The qualitative data will provide rich detail in what is currently uncharted terrain. We will be able to track trajectories of long Covid; international opinion is that its relapsing-remitting nature requires this (24).

Box 1 Inequities for ethnic minorities (including migrants) and those with chronic conditions/disabilities increasing their risk of poor pandemic health outcomes (2,3,11)

1. **Increased risk of isolation, abuse or neglect**, **poor access to informal emotional and wellbeing support**, due e.g. to national pandemic responses, stigma, changed activities, priorities, attitudes of others, a state of 'normalized absence, pathologized presence' (25).
2. **Inequitable formal treatment, support and care** from attitudinal, structural, policy, cultural, linguistic, communication and economic barriers, leading e.g. to difficulties implementing recommended COVID-19 avoidance strategies, vaccine mistrust, and risk of severe illness.
3. **Psychosocial factors raising COVID-19 risks, reducing capacity to cope** with social, economic and psychological pandemic impacts, including worries about people 'back home'.
4. **Unemployment/reduced income** (e.g. zero hour contracts; 'no recourse' to welfare).

Deleted: 1
Deleted: and the 1%
Deleted: for
Deleted: particularly
Deleted: during the pandemic
Deleted: thus
Deleted: they are
Deleted: This heterogeneity
Deleted: is necessary to
Deleted: but
Deleted: e
Deleted: falling into the trap of
Deleted: other
Deleted: report
Deleted: separately
Deleted: A
Deleted: are
Deleted: for
Deleted: that we
Deleted: , using **mixed methods**
Deleted: This means
Deleted: will
Deleted: enable us to gain
Deleted: on this
Deleted: trajectories
Deleted: it is
Deleted: ly
Deleted: acknowledged
Deleted: of long Covid
Deleted: including

Terms used: For simplicity, despite its problems, after PPI discussion, we use the term **migrant** for someone born outside the UK (as per UK policy) who intends to stay in the UK for 1+ years, including asylum seekers and refugees. We use '**ethnic minority'** to encompass migrants and 2nd generation ethnic minorities (i.e. UK born). We include any **condition/disability**, including self-diagnosis, that chronically affects daily activities (e.g. diabetes, dyslexia, chronic pain, loss of limb, depression, autism). We will record conditions, but group by Dietary and 5 UK Family Resources Survey themes: Mental, Mobility, Stamina/breathing/fatigue, Hearing/Vision loss, Developmental/intellectual. We include long Covid and other multisystemic conditions (which may belong to more than one group); our categorisation by impact not diagnosis enables a practical focus and a flexibility to changing understandings of long Covid. **Long Covid** is defined by NICE (26) as: "*Signs and symptoms that develop during or following an infection consistent with COVID-19, continue for more than 12 weeks and are not explained by an alternative diagnosis. It usually presents with clusters of symptoms* [this may be more than 4 hence the Covid Symptom Study (https://covid.joinzoe.com/blog) under-reports long Covid]*, often overlapping, which can fluctuate and change over time and can affect any system in the body."* According to the UCL/Oxford symptom survey (28) the most common ongoing symptoms in 201 long Covid patients (only 18% were hospitalised), were fatigue (98%), muscle ache (88%), shortness of breath (87%), and headache (83%), according with other long Covid studies.

EXISTING LITERATURE AND STUDIES

The impacts of COVID-19 on the lived experiences and services access of ethnic minorities or PwCD, at the time we submitted our stage 1 application, were only considered within published papers in commentaries and calls for action. The evidence base remains much smaller than the overall COVID evidence base despite the greater impact of COVID-19 on these groups.

Ethnic minorities: A simple scoping search of the more than 30,000 **articles** in the EPPI COVID living map of evidence (https://bit.ly/3npqGtz) for 'ethnic*' or 'minority' or 'minority ethnicities" or 'black' or 'BAME' retrieved 67 hits, with only two directly relevant to the proposed study (in our first work package we will extend our literature search to increase its sensitivity). Both were US surveys. One, surveying ethnic minority asthma patients and doctors, reported socioeconomic factors and institutional racism impacted on asthma care in the pandemic; 25% of doctors found it more challenging to care for black patients with asthma during COVID-19 (28). The other showed pandemic telehealth was most used by black patients, attributed to their need to compensate for prior health and health care disparities caused by systemic racism (29). Both studies therefore support the need for our study. Given the rapid development of COVID-19 research it is important to also consider where our study fits with **ongoing studies**. As of December 2020, none of 248 studies on the NIHR site 'Be part of research' specifically considers the health/social support experiences of ethnic minority groups. Among jointly funded UKRI-NIHR studies, none has our focus on producing practical strategies and modifications to existing support and care that can be immediately implemented with minimal/low cost and effort, though several consider alternative aspects of the ethnic minority experience such as COVID-19 infections or migrant working, or engagement with pandemic information. **Outside of the pandemic**, the few studies (e.g.30-35) of the post-resettlement lived experience of recent migrants to the UK have a different focus/intent and no formal social network analyses. There are more studies of 'cultural competence' in healthcare (e.g. by team members, 36-37) but though their findings support the need for this study, they tend to a narrow focus on settled single ethnic groups with one specific condition (and one of the condition/disability impacts we consider) and do not transfer to the current situation or cover conditions similar to long Covid. This shows how our work is important and will provide much needed information that could be extended to and tested in non-pandemic situations in the future.

***Chronic conditions or disabilities*:** Our EPPI-map scoping search found several 100 **articles** using the search terms 'disability' or 'condition' or 'illness' (to be extended in work package 1) but most were survey or audit-based considerations of reduced non-COVID patient footfall. In a global COVID-19 survey, 17% of 548 respondent rheumatologists estimated 25% of their patients had no access to telehealth (38) showing the need for alternative strategies such as we aim to explore. Interviews with 7 disability NGO representatives in Italy highlighted bureaucratic challenges, and a lack of advice, coordinated care plans and inter-agency coordination to compensate for reduced services (39). Small COVID surveys inside and outside the UK have shown the negative impact of reduced access to treatment on patients' symptomatic control, for Parkinson's Disease (40), migraine (41), rheumatology (42) and chronic refractory neuropathic pain and their increased reliance on support networks (43). Our study goes beyond these studies of impact, to focus on strengths, assets and solutions to issues. For example, small cross-sectional analyses suggest some chronic conditions and disabilities may confer

resilience to mental health or wellbeing effects of the pandemic (44,45) while a UK pandemic analysis of chronic fatigue Reddit posts reported more severe symptoms in some people but also more accessible opportunities to interact (i.e. online videocalls) (46). In the UCL UK COVID-19 Social Survey (18), which explores psychosocial health in the whole population and so has a different focus to us, 38.2% of 51,417 analysed respondents have pre-existing physical conditions (with a much narrower definition than ours), 19.9% pre-existing mental health conditions and 12% are from 'BAME' groups. This study found good support and resource access protective for pandemic mental health which has informed our study design. Thus the limited evidence supports our study and there remains the need for the insights, consideration of multiple intersecting factors and outcomes and particularly generation of solutions, such as we aim to provide. We found no relevant **studies** on the NIHR site 'Be part of research'. Considering other studies, including jointly funded UKRI-NIHR studies, three are most relevant. One considers the response of organisations who provide services for refugees and asylum-seekers (through 20 interviews) against the lived experiences of the people they support (40 interviews), in Scotland and Newcastle-Gateshead, combined with a UK wide two-wave survey specifically targeting asylum-seekers and asylum services (Hopkins, Newcastle). This focus is slightly different to ours. Shakespeare (LSHTM) is conducting 60 in-depth telephone interviews with a range of disabled people, including parents of disabled children, with different conditions, across England and Scotland, as well as with 15 key informants, repeated at six months. This study, smaller than ours and single-method, has a different sampling frame and limited ability to consider intersectionalities. There are studies of people with intellectual disabilities (Hastings and Hatton at Warwick and Lancaster) or with dementia and their carers (Banerjee in Bristol plans over 250 telephone interviews and Clare in Exeter up to 700); these groups require specific considerations in study design and the consent process so we do not target them in recruitment, though we do not exclude them. A qualitative study (McHale, Birmingham) considers impacts of the legal suspension by local authorities of the application of certain provisions under the Care Act 2014 as part of COVID-19 emergency powers. Several small-scale studies by support groups or local clinics are in progress to consider long Covid lived experiences. In none of these studies is there a focus on assets and solutions. None considers the combination of chronic condition/disability AND minority ethnicity. All are complementary to our own study.

EVIDENCE EXPLAINING WHY THIS RESEARCH IS NEEDED NOW

As we show above, our work addresses a still largely unmet expressed need in pandemic research to focus on PwCD and ethnic minorities pandemic experiences. Over our study period there will be a need for more focus on 3 specific areas of chronic health and disability, all more critical in our focal group:

Deleted: of which will be

1. New conditions/disabilities that develop or old ones that worsen because of reduced services and other structural consequences of the pandemic (which have widened pre-existing inequities for PwCD and minority ethnicities) (10)
2. New conditions/disabilities that develop or old ones that worsen because of lifestyle changes or neglect of health during the pandemic or pandemic responses such as shielding
3. Long Covid as an emergent chronic condition.

The greater impact of the pandemic on PwCD and ethnic minorities is a human tragedy and care issue (1), costly at personal, community, healthcare, economic, societal and human rights levels. The impact is likely to increase through our study across these 3 areas, given the negative impacts on population health. Our community-based mixed methods longitudinal approach is designed to consider this and inform and shape the immediate and future health and social care response particularly for ethnic minorities with chronic conditions/disability, and to take account of future pandemic impact and uncertainty. We believe there is considerable synergy and learning potential to considering both existing chronic conditions/disabilities and **long Covid,** since symptoms of long Covid and some existing conditions correspond and an underlying mechanism-in-common may be MCAS (47) (though there are alternative explanations (1)). In this regard our study is both unique and particularly important now for future planning considerations; the lessons we can learn from existing conditions are likely to be transferable to people with long Covid and their health and social care and vice versa. Policymakers such as those within Public Health England (PHE), and practitioners such as clinicians and social support workers, lack but need the evidence we will provide on PwCD and ethnic minorities for equitable care, as the statistics at start of this application make clear. PHE specifies an urgent need for participatory work with minority ethnic groups (13) such as will undertake. With 3 waves of data collection, we can report interim findings at 3, 9 and 16 months for rapid impact. Given the rapidly changing nature of the situation and the considerable impact that care issues are having on the nation's health and wellbeing, it is important for policymakers and practitioners to have timely contemporary

Deleted: are similar to

Deleted: there has been suggestion that

evidence in this way that they can act on quickly. Our work on both health and social care across multiple levels (see Theoretical framework p6) fits well with the new NHS plans for integrated care with system-working across NHS providers, primary care, local government, social care and the voluntary sector (48). Notably we also consider vaccine uptake. Respondent and national UK demographic data will be compared for representativeness and transferability explored at each stage. Our co-create and advisory groups – currently planned as remote work - will explore how to translate findings at each wave into practice in the 'real world' to ensure implementable, relevant, rapid, useful policy and practice underpinned by implementation science (49). We also expect rapid impact from our strong networks and Co-A and collaborator built-in considerable pathways to influence (see Outputs p17).

AIMS AND OBJECTIVES

Our solution-focused aim: We aim to contribute and inform evidence-based formal and informal strategies, guidelines, recommendations and interventions for health and social care policy and practice during and after the COVID-19 pandemic and system recovery (including any future waves), to mitigate inequities and improve the experiences and health and wellbeing outcomes of minority ethnic groups at the intersection with chronic conditions/disabilities. To do so, we will develop a rich understanding of their mental and physical health, coping, access to resources, and informal and formal social and health care support experiences, and relevant assets and strengths, longitudinally over 18 months using mixed methods, examining variations through an intersectionality lens. Analyses, outputs, dissemination and implementation plans for these will be co-developed with key stakeholders (see 'Key Audiences').

Objectives: Using an intersectionality lens our objectives are to:

O1: Explore and compare, by location and time, survey and qualitative data on changing patterns of need. Including intersections of chronic condition/disability and ethnicity/citizenship state with UK pandemic contexts.

O2: Relate pandemic coping strategies/solutions to O1 findings, including what worked well or less well, and touchpoints (where experiences might best be improved), to inform health and social care policy and practice

O3: Use Social Network Analysis to explore formal and informal network issues/affordances in health and social care solutions

O4: Gain insights from comparisons and relationships across our mixed methods data, rapid framework-based synthesis of the published and grey literature, and secondary analyses of UCL's Centre for Longitudinal Studies (CLS) and ActEarly COVID-19 specific surveys.

O5: Contextualise and explore transferability of qualitative findings using the survey, and survey findings using CLS/ActEarly UK census data.

O6: Co-create with stakeholders (including PwCD/minority ethnicities) interim/final outputs that include identified strategies, interventions and touchpoints, and plans for rapid pathways to impact.

Our intersectional lens enables:

- potential stratification by risk levels to inform preventative/care action
- suggested strategies and interventions appropriate for different intersecting structural, cultural and religious needs, levels of deprivation, ages, gender and other factors shown through our research to be relevant.

Access to resources, formal and informal care, social networks and links to health/social care outcomes are foregrounded as these are protective for pandemic mental health (18,50) and the wider non-pandemic literature suggests psychological and social support factors enhance general wellbeing (21).

RESEARCH PLAN / METHODS

Our 4 work packages (WP) involve a new UK survey in 3 waves, parallel qualitative insights, secondary analyses of other surveys, rapid review, and outputs for immediate use developed with participatory methods. Our approach, remote, takes account of pandemic constraints. We can easily revert to face-to-face work if appropriate. Our study is ambitious and we have therefore put in place a team that is particularly well placed to achieve this work (See Expertise, p20).

Theoretical framework: Embodied experiences of chronic conditions and disabilities, being shaped in and through social interactions (including with health and social care and support) (51), are necessarily intersectional with areas of potential discrimination and oppression (Box 1) across the levels of **Bronfenbrenner's socioecological model (SEM**) (52), hence the need for a range of comparisons and involvement of multiple stakeholders in our study. This model will frame our translation of findings into clear recommendations for varied audiences and fits with the new NHS tiered Integrated Care plan (48). The model levels range from smaller, proximal settings of local influence to larger, distal settings

Deleted: national longitudinal cohort collection (CLOSER)…

Deleted: CLOSER

with indirect influence. The bidirectional and dynamic nature of intersectional interactions across the levels means mutual constitutions are in constant flux, emphasising the need for a longitudinal study. Our work is also underpinned by the **Consolidated Framework for Implementation Research** (CFIR) (49) because, as an amalgamation of a range of existing implementation theories, it cuts across the levels of the SEM, with a comprehensive range of constructs. The CFIR is easy to operationalise, flexible (the user selects only themes from a pool of 39 that are relevant), and provides actionable findings across multilevel implementation contexts. These theories will inform interview topic guides, and survey questions for topics 3, 4, 7 and 8 in the next section. In our analysis they will inform health and social care mapping and implementation considerations across multiple levels for integrated care.

Topics across all WP: These are as follows (in the survey based on questionnaires, mostly validated in ethnic minorities including recent migrants) and include a range of variables that the research evidence suggests are key influencers of pandemic health and wellbeing. We believe by focusing on these, we should have a big impact through small changes. We note that other variables such as access to education will also be important but are not of direct relevance in terms of our aims:

1. **Intersectionalities,** the importance of which we explained on page 1 (we use a recently developed framework [53])
2. **Behavioural responses** to COVID risk-reduction measures including vaccination by individuals and their formal/informal support and care networks (e.g. friends, family, community, health/social care) – to understand the context of peoples' lives, what responses are feasible or acceptable to them, and effects on their networks. This will help us build up a picture of potential assets and strengths and affordances (as well as issues).
3. **Access** to resources, formal/informal support and care, including **digital transformation**, service innovations – as explained on p 3, good support and resource access is protective for pandemic mental health (18), and it also mitigates other health issues.
4. **Social network** (formal/informal support and care networks) descriptions - the importance of which we describe on p2 (contextualising topic 2 above for network behaviours)
5. **Coping and attitudes, physical and mental health** consequences of the pandemic, why they arose and how issues can be mitigated.
6. Mental and physical wellbeing/**quality of life** as core outcomes
7. **Local/regional differences** in responses linked to policies/interventions and associated impacts
8. **Future** policy implementation that is accessible to PwCD and minority ethnic groups.

In all cases we will consider what has worked well and less well for health and wellbeing outcomes, ensuring policy-relevant comparison and synthesis across WP.

WP1: SECONDARY ANALYSES (1-3 AND 14-17 MONTHS) (for O4) (cost £150,437 FEC)

This stage informs WP2-4. We will map and synthesise existing quantitative and qualitative evidence on the pandemic and PwCD or ethnic minorities that supports our aims.

Textual data on our 8 topics: Given the novel and unstable nature of the pandemic, its sequelae and system recovery, this includes pre-print resources such as medRxiv and less formal sources such as blogs, Google, Reddit and Twitter searches. Also more usual database searches using UCL's EPPI-Reviewer 4 software, the UCL EPPI centre's COVID-19 Living Map of Evidence, the WHO Global Research Database on COVID-19, the COVID-19 Rapid Evidence Reviews Group (CORRE), and LitCOVID. These databases bring together evidence on COVID-19 from a worldwide dataset; we will add other sources that we locate. **Social media searches** will be run on identified hashtags and frequent users who discuss our 8 topics, tailored by site in consultation with our PPI group. Relevant threads will be analysed using open-source bespoke software e.g. GATE (54), including twitter network maps, and thematic content analysis. Extracts used in dissemination will be paraphrased to avoid identification. For **articles, reports, and longer texts**, we will search using controlled subject headings and keywords such as 1) COVID-19 OR COVID OR SARS-CoV-2 or pandemic; 2) long-term OR post-acute OR long-tail OR persistent OR chronic COVID OR long Covid OR post$discharge OR prolonged symptoms OR long haul; 3) ethnic* OR minority ethnic* or variants thereof including specific groups; 4) chronic OR long$term OR underlying OR [names for chronic conditions or chronic health states] OR disab*; 5) specific terms related to our 8 topics running through the study (e.g. for topic 6 synonyms of 'wellbeing' OR 'quality of life'). Two reviewers will screen titles, abstracts and full texts against inclusion criteria, with disagreements resolved by 3rd third researcher. We will use quality assessments designed for each study type, with additional reference to the provenance and publication status of sources. Data will be extracted by one reviewer and checked by a 2nd. We will use descriptive statistics and meta-

Deleted: lack of peer-reviewed published material, the

analysis to summarise data where appropriate, and otherwise narrative synthesis. We will perform subgroup analyses (see WP3 for potential research questions for these). These data will give us a grounding in current research and other evidence, in a fast-moving pandemic-responsive field, to ensure we tackle our aims in the most useful way, and will help to provide themes to incorporate in the WP2 and WP3 data.

CLS and ActEarly cohort studies: We will undertake secondary analysis of a subset (those with chronic conditions/disabilities) of the UKRI-funded **ActEarly city collaboratory** consortium (55) COVID surveys. These complement our surveys and are similarly supported by qualitative data. ActEarly collects data of interest in two ethnically diverse areas, Bradford and East London, on physical health (including general health, health anxieties, health behaviours and mental health), relevant demographic factors, services access, and family relationships and social support. Co-applicant Dickerson, Director of the Better Start Bradford Innovation Hub and Bradford Inequalities Research Unit is Programme Manager of the Born in Bradford (BiB) partners of the ActEarly consortium. The London partners include members of the PI's department and our Bromley by Bow collaborator. We have chosen Bradford and London as two of our own sites for the same reason as this consortium and hence our work will be complementary. Both areas have strong reputations in applied health research with a focus on health inequalities in deprived and ethnic minority populations and deep engagement with the community and local policymakers. Inequalities are extreme in both areas, making it likely that strategies successful in mitigating the adverse impacts of COVID-19 there are likely to be transferable to other places with less extreme conditions. In both areas, research groups represented by Dickerson and Bromley by Bow Centre, working with policymakers have built strong networks across health care providers, connecting multiple systems. The initial focus of the ActEarly Covid-19 surveys has been on children, parents and pregnant women (a sample pool of just under 14,500 in Bradford (56) and a focused sample of 2000 in East London), which avoids participant research burden from our study. **CLS**, part of the PI's UCL department, has run COVID-19 surveys within the nationally representative cohort studies it curates, with respondents aged 19-74: Millennium Cohort Study (born 2000-02), Next Steps (born 1989-90),1970 British Cohort Study, 1958 National Child Development Study, and also the MRC's National Survey of Health and Development (1946 British birth cohort). Wave 1, with over 18,000 respondents, took place in May 2020, and Wave 2, with almost 26,000 respondents, in September 2020. These included items on physical health (including COVID-19), health behaviours, demographics, mental health, social connectedness and health care, hence relevant to our aims though the fit is not as close as for ActEarly. We will also draw on UCL's COVID-19 Longitudinal Research and Evidence Tracker which trawls for COVID-19 longitudinal research and evidence, e.g. briefing notes, reports, articles. Evidence from the tracker may provide further useful information and context.

Deleted: *CLOSER*
Deleted: 2020-21
Deleted: data
Deleted: **CLOSER (Cohort and Longitudinal Studies Enhancement Resources)**
Deleted: the CLOSER
Deleted: the

Data synthesis: This follows a previous design for COVID mental health care data (17) to produce a narrative synthesis of text data, as a mapping review, and thematically linked summary quantitative analyses from the cohort studies. A matrix-based approach will be used for initial mapping, with sources as rows, themes as columns. Our initial 1-3-month work will be updated in months 14-17.

WP1 Evidence outputs:

1. Identification and quantitative/narrative summary of themes considering the pandemic's impact on PwCD and/or minority ethnicities, that focus on our 8 topics (p6), from each of:
   a. the peer reviewed and grey literature
   b. social media
   c. existing cohort studies
2. Identification and quantitative/narrative summary of themes considering access to resources, formal and informal care, social network and mental and physical health/wellbeing consequences of the pandemic on PwCD and/or ethnic minorities from the same 3 sources
3. An understanding of the pandemic lived experience for PwCD and/or minority ethnicities
4. Research findings to inform and be synthesised with WP2-4.

WP2: NEW SURVEY (2,8,15 MONTHS) (O1-5) (cost £195,303 FEC)

Our new survey considers our 8 core topics (p6), informed by WP1 secondary analyses, in 3 waves, for 1st and 2nd generation community-dwelling minority ethnic groups and white British comparators, all with/without chronic conditions/disabilities, across the UK's 4 nations. It enables us to determine relationships between variables and trajectories relevant to our aims. We scoped other COVID-19 surveys and as explained in our Review of the literature on p3 and for WP1 cohort studies, all existing surveys have considerable differences to ours. An existing similar survey undertaken by colleagues of the PI in Tower Hamlets and the BiB survey, both for ActEarly, have provided a testing ground, and we

will use the data in WP1. These surveys consider pregnant women and families with young children, with a focus on childhood; our survey is thus unique and extends and enlarges their scope.

Analysis will exploit all 3 waves of the data, with these **research questions (RQ):**

1. *How do outcomes (resource access, formal/informal care, quality of life, control of life, physical and mental health, social networks) and outcome trajectories differ by sample subgroups?*
2. *What are the outcomes (resource access, formal/informal care, quality of life, control of life, physical and mental health, social networks) and their trajectories in terms of intersectionalities?*
3. *To what extent can pandemic prevalence and adherence to social distancing at the area level explain differences in outcomes and outcome trajectories across subgroups of the sample and in terms of intersectionalities?*
4. *How do the outcomes inter-relate within and across survey waves, and how does this differ across subgroups of the sample and in terms of intersectionalities?*

A full-time post-doctoral researcher will undertake the main survey work. We will develop the final survey format with our PPI group and pilot it with N=30 before fielding it at scale. The survey will be online but with telephone interviews (CATI) where needed. To minimise respondent burden, each wave will be completable within 15 minutes, which ActEarly and CLS work shows is acceptable for COVID-19 surveys. Surveys will begin with informed consent/screening questions. Then the study topics will be explored, based on validated questionnaires, mostly validated in migrant and ethnic minority groups as well as the dominant population **(Box 2).** Surveys in different waves will differ. First, theoretically stable concepts (e.g. tolerance to uncertainty, demographic characteristics) will be measured only in one wave. Second, key topics identified in prior wave(s), qualitative work (WP3) and our co-production and engagement work running through the study may be added. But key outcome and exposure variables that we expect to change during the pandemic as specified in our WP2 RQ will be measured in all three waves to study trajectories.

Box 2. Our 8 topics and corresponding survey instruments/items

**Topic**	**Questions**
Intersectionalities	Demographics including year of birth, gender, ethnicity, relationship status, area code of postcode, urban/rural dwelling, accommodation type, household income, education, employment status, religiosity (57)
Behavioural responses ‘	Control of life’ (including COVID-19-related)
Access to resources, support, care, vaccines	QOCS–ID (58), Vulnerability Assessment Framework (59) for care needs, UK government SAGE group recommended questions (https://bit.ly/2OZN9Bf)
Social networks	Developed from the close persons questionnaire (60)
Mental and physical wellbeing/quality of life	WHOQOL-BREF-ID (58)
Coping	Including tolerance to uncertainty, positive appraisal style, attitudes to being ill/disabled (WHO ADS (58)), health and mental health consequences (Global Mental Health Assessment Tool (61)) of the pandemic, why they arose and how issues can be mitigated
Local and regional differences	Apart from within-survey analysis, we will match respondents’ area code of postcode with area-level (i) registered COVID-19 cases, hospitalisations and deaths (ONS https://bit.ly/2NOydC8) and (ii) social distancing adherence (Google Community Mobility https://bit.ly/2AqRwyk).
Vaccines, Future policies	Freetext comment boxes

Quantitative analysis: A **descriptive statistical summary** will be updated with each wave using RedCap analytical tools for rapid dissemination. For **RQ1**, we will exploit the longitudinal nature of the data using **Latent Growth Modelling** (LGM) to estimate (i) levels of network capital, quality of life, mental and physical health and other key variables, and (ii) change in these over time, and differences in levels and changes between those with chronic conditions versus without, white British versus ethnic minority, and citizenship versus without. For **RQ2**, we will carry out “multiple group” analysis with separate levels and trajectories of the key variables estimated per minority, condition/ disability and citizenship intersectionality. In other words, we will vary different combinations to consider the effect of intersectionalities on outcomes. For **RQ3**, we will include area-level matched data on pandemic

Deleted: and

Deleted: across

Deleted: s

Deleted: of the sample

Deleted: and

Deleted: outcome

Deleted: CLOSER

Deleted: (e.g. quality of life, access to care, mental health, networks) …

prevalence and adherence to social distancing in the latent growth models as covariates, to explore the extent they can explain differences in outcomes between each subgroup, and differences compared with inter-sectional combinations. Depending on geographical coverage and the numbers recruited, the LGM estimation could be carried out within-area, to examine the causal impact of the change in pandemic prevalence and social distancing across the waves on each key outcome, under the assumption that change in pandemic prevalence and social distancing is exogenous. We will examine the plausibility of this assumption and detail possible sources of endogeneity. **This will be important for policy, with its unique focus on ethnic minorities and PwCD.** For example, our data could help clarify why specific groups may not find it feasible to adhere to recommended behavioural responses. Finally, for **RQ4** we will estimate a developmental cascade model, including all 3 data waves and key variables, to explore how the key variables are associated with one another, both within survey wave and over time. We will fit the LGM models using Structural Equation Modelling (SEM); this offers useful tools for dealing with missing data due to non-response and attrition; the Full Information Maximum Likelihood estimation will be used for possible systematic missingness. The **Social Network Module** will provide an ego(participant)-centred network, including ego's ties, their frequency, resources of the alters (others), relationship types. Further characteristics e.g. the ego network's size, density, composition, and average strength of ties will be calculated and a latent "network capital" variable created through measurement analysis within the SEM as a novel contribution. A Confirmatory Factor Analysis will refine other survey measures.

WP2 Evidence outputs:

1. Identification and description of how pandemic-relevant outcomes (resource access, formal/informal care, quality of life, control of life, physical and mental health, social networks) and outcome trajectories differ across subgroups of people of different ethnicities including white British and with or without chronic conditions or disabilities.
2. Identification and description of outcomes and outcome trajectories in terms of intersectionalities
3. Identification and description of any emerging associations over time and within cross-sectional subgroups, including in terms of a) COVID-19 case prevalence and adherence to social distancing at the area level, b) long covid and vaccine uptake/hesitancy, and c) intersectionalities
4. Risk stratification of groups to inform preventative/care action
5. Research findings to inform the early WP3 work and for synthesis with WP1, WP3, WP4 findings.

Deleted: and

WP3: QUALITATIVE WORK (3, 9, 15 MONTHS) (O1-5) (cost £306,849.55 FEC)

In-depth insights will be added from 3 qualitative waves exploring the experiences of 1st and 2nd generation community-dwelling Arabs, Poles, Indians/Pakistanis and sub-Saharan Africans, and, for comparison, white British, all with/without chronic conditions/disabilities. **WP1** and **WP2** findings will feed into this, contributing possible questions for our topic guide, which will be designed around the same topics as shown in Box 2 and p6. All qualitative data sessions will be recorded with consent and transcribed. Interviews will last up to 1 hour, workshops 2 hours. Participants will receive thank you vouchers (online, posted or by hand depending on extant pandemic guidance/personal request).

WAVE 1: SEMI-STRUCTURED INTERVIEWS

Remote by default, will use the method of respondents' choosing (most likely phone (62)). At each site, our PPI leads will train a lay community member to undertake interviews locally –still planned remotely - supported by 2 central qualitative researchers who will also undertake remote work.

Many participants are likely to depend on informal **support networks** for: education; health, social care, legal matters; wellbeing; social/cultural activities (21). These, formal support and third sector networks have been disrupted during the pandemic. We explore the issues through:

- A brief questionnaire about social networks preceding the first interview – orally if needed
- Results translated into Network Canvas software to develop ego(participant)-centric network maps that can be explored in depth in interviews
- Participant sketch-maps of their local area and the places significant to them.

For inclusivity, important when considering people with disabilities and ethnic minority/migrant groups, participants will be asked to take smartphone photographs of significant places prior to interview. This ethnographic approach facilitates a safe social space to communicate difficult issues and has been used to explore migrant resettlement (63). We will give all participants clear instructions, with a focus on ethical issues (e.g. to avoid identifiable photos of other people). Participants with no/unsuitable phones, no internet, limited data plans will be given **disposable cameras** with SD cards (local researchers will arrange pandemic-safe digital data collection). Participants will be asked to take photos

significant to their healthcare interactions and to their social interactions. Photos will be discussed in interview, to probe for insights, and will also be thematically analysed as data. They might for example show physical barriers to accessing a building, or a photo of a restricted gathering for a wedding or a funeral, which can be used to stimulate discussion about our key topics. Interview data will undergo Keyword in Context (word frequency-based) analysis and constructs used to develop a coding frame for Framework analysis of the workshop, interview, photo and key informant data, for general dissemination and policy-relevant themes that can be mapped to the survey for added insight. Added to this deductive approach we will allow for inductive themes. Data collection and analysis will be concurrent for quick outputs and to test emerging and discordant themes and will continue until sufficient 'inductive thematic saturation' is reached. We will undertake discourse and narrative analyses on a data subset produced from participant pairs matched on features identified as important from analysis. This subset approach, used by Rivas successfully before (64), makes optimal use of the large dataset. The discourse analysis will examine the linguistic and discursive resources participants use to link social practices (e.g. ableism, racism) to their situation and with the narrative analysis of participant 'stories' will further contextualise findings on wellbeing and coping, social identity and networks, attitude to and relationship with services - important for services to build trust. Black peoples' mistrust of the COVID vaccine is relevant here for example. These analyses will reveal potential mechanisms for behavioural responses to help us develop our evidence to decision interrogation (WP4). We will ensure credibility/internal and external validity (e.g. through exemplar data extracts, data collection triangulation, and team and participant workshop data discussions), transparency (with a clear audit trial) and reproducibility (with thick descriptions of context and analysis) and anonymised data will be archived for secondary analyses.

Deleted: within typologies identified from Framework analysis.

Deleted: will explore consistencies and variation across the data, makes optimal use of the large dataset, was

Deleted: and will help illuminate participants' wellbeing and coping, attitude to and relationship with services - important for services to build trust

Deleted: Relevant theories will be considered, e.g. biographical disruption (65), which is widely used to explain how people experience and manage acquired long-term illness and disability.

Deleted: transparency

WAVES 2/3: RESEARCH WORKSHOPS

Likely remote, due to the pandemic, e.g. using 'Teams'. To avoid excluding people, we will also offer repeat interviews. The precise make-up/number of workshop groups per wave will be determined from Wave 1 data. We will work with the UCL Centre for Collaboration and our PPI group to ensure workshops are accessible and inclusive, e.g. incorporating Padlet, Miro and other visual tools or infographics where appropriate. Wave 2 workshops will discuss scenarios, or **structured vignettes**, shown as short videos recorded by community members reading scripts; content will be developed from Wave 1 data into a pandemic-relevant story, illustrated e.g. with Wave 1 photos with permission, to consider assets and strengths, issues and potential solutions. Non-identifiable verbatim phrases will enhance authenticity, with accessibility transcripts provided in advance. This approach is effective in inclusive research and suited to both remote and face to face work, so we can be flexible. Discussion will serve to check validity of previous findings and consider changes from these. Wave 3 workshops will be similar, with updated vignettes. Team members have used a similar approach in previous (36) or current (Rivas: CoGS, Crescendo, see cv) projects. We will also use **participatory scenario planning** (66), a policy tool whereby participants are encouraged to imagine and explore alternative futures, their impacts and relevant action plans (topic 8, p6).

CO-CREATE WORKSHOPS through the study (O6, cost incorporated in other costs)

are distinct from WP2 research workshops though based on similar principles. Key differences are that they will: a) include a range of other stakeholders as well as ethnic minorities with chronic conditions/disability; b) contribute to analyses in addition to translating findings into practice. All 3 workshop sets (wave 2, wave 3, co-create) will aim for outputs relevant and implementable for the 'real world' that maintain participant voices, with tangible benefits for all. Materials will be shared in advance, to suit accessibility needs, and discussions led by our PPI lead and PI. Sessions will last 4 hours (2 if remote), with practical activities that empower all those attending to contribute as equals to 'negotiated' analyses/outputs. Group discussions will be summarised for reference. The research team will work on outputs/analyses to present at following workshops so outputs/analyses are developed iteratively through the co-create workshops.

WP3 Evidence outputs:

1. Characterisation of social network issues/affordances that can inform health and social care changes and improve pandemic health and wellbeing for ethnic minorities with PwCD
2. Framework and word frequency analyses for practice- and policy-relevant themes that can be mapped to the survey findings for the same topics
3. In-depth, theoretically-informed understanding of lived experiences of ethnic minorities with PwCD - important for services to build trust

4. Description of imagined alternative future scenarios to help with future policy and practice planning for ethnic minorities with PwCD
5. Research findings to be synthesised with WP1, WP2, WP4.

WP4: PARTICIPATORY IMPACT/OUTPUT DEVELOPMENT (O6) (cost £178,351 FEC)

1. 15-25 **interviews** (16 months) (up to 5 per site) are planned **with key informants** as determined from other WP: e.g. welfare, social and health care staff, settlement and ethno-specific services, the third sector and community leaders. To support implementation into policy and practice, topics include perceptions of local service needs, area-level characteristics, barriers and facilitators to community member service access, current community-led responses, impacts and effects of the pandemic, and future planning. Topic guides will also consider CFIR themes (49).
2. **Data synthesis** will provide an executive overview for easy digestion by policymakers and practitioners and help show where health/social care policy and practice changes are likely to be most effective. Synthesis will be results-based, with thematic tabulation derived from WP1-3 data analyses, with table columns for themes, rows giving quantitative and qualitative data. Some data will need to be transformed (quantified or qualitised) for tabulation e.g. network graphs. We will interrogate the tabulated data using anchor questions based on the PerSPectif framework (67) (informed e.g. by data convergence/divergence patterns). This will form the basis of guidance and recommendations outputs and will inform interventions. An example question might be: *"From the **per**spective of a first generation single woman aged 18-30 of African descent, with complex dietary needs, in a rural Yorkshire **s**etting, how does the **p**henomenon of community-based food banks, within an **e**nvironment of poor transport infrastructure and geographically remote facilities (a food desert) **c**ompare with online shopping during a **t**ime of national lockdown in relation to **f**indings on their perspectives and key study outcomes*". Specific questions will be based on data findings, e.g. intersectional variables and outcomes. Disaggregation will be built in where possible and useful, though in a complex systems approach this may sometimes be misleading due to system interdependencies. Systems diagrams, logic models and other approaches will be used where appropriate. This evidence to decision approach supports our aim to report explanatory findings with practical application.
3. We will try out adaptations of two existing training programmes, **Tough Cookie** for community members and **Pain Relief Management** for practitioners, based on our findings, at our 5 sites for proof-of-concept. We do not plan to test any other interventions (please see the 'Team' section for details of these programmes)
4. Our final mixed stakeholder a) advisory group and b) **participatory (co-create) workshop**, CIFR-themed, with our PPI group and key informants will develop with us the final implementation, dissemination and impact plan.

Deleted: Through **synthesis** methods we will map comparable data together on a matrix and compare them for illuminating convergences and divergences across our WP, by method, time, other key variables which goes beyond triangulation. Importantly, this

SAMPLING

WP2 Survey sampling: We will **recruit** via social media and national networks (e.g. academic, NHS, third sector) including our existing networks and mailing lists, and large databases of adults interested in health research across the UK. These include UCL BioResource and HealthWise Wales, the NIHR Be Part of Research, the NIHR Research Design Service Public Involvement groups and networks registries, the UKRI Mental Health Research Networks, The Covid-19 Research Involvement Group, The Covid-19 Support Group. We will also recruit via the various social media patient and migrant groups with which we are connected and the specialist third sector organisations in our Team section list. This enables good reach and access across the spread of chronic conditions/disabilities, ethnic minorities and citizenship states and connection with people already wishing to take part in research. We also have access to the ActEarly city collaboratories in Bradford and East London if needed, and the Gateshead long Covid clinic being set up by Saravanan, though these will be a focus rather of WP3 recruitment. We recognise our recruitment, being non-randomised, will be biased, for example to those already interested in research participation or who are active users of third sector sites and have online access. We will compare respondent demographics to whole population estimates where possible (though formal data are limited) to explore **representativeness**.

Survey numbers and power: The primary aim of the quantitative data is to describe the trajectories of key variables and outcomes (e.g. quality of life, services access, networks), and the links between them, in ethnic minority and PwCD communities, in comparison to the White British. The longitudinal survey will not be used to test a particular treatment or focus on a single effect. Considering power in Structural Equation Modelling (SEM), the required sample size depends on several factors (68). First, the required

sample size increases with the number of latent variables, but at a decreasing rate (i.e. the required sample size difference between a model with one versus two latent variables is larger than that between a model with three versus two latent variables). Second is the size of the loadings on latent variables. Required sample size decreases strongly as the loadings increase. Finally, power increases as the number of items used to measure each latent variable increases. In our basic SEM, we have six core latent variables per wave: quality of life, control of life, access to care, coping mechanisms, mental health, and social networks. Each will be measured by several items (the average number being more than 8). In a worst-case scenario with average loadings of around 0.5 and an item missingness of 20% (as suggested from ActEarly work by co-applicant Dickerson, a sample size of 800 per subgroup per wave will yield useful analyses. We have four main subgroups (i.e. minority ethnic, minority ethnic+ PwCC, White British, White British + PwCC). Hence, a sufficient target sample size is 800*4=3,200 though we aim for 5,000 for stronger data (68).

Survey target number risks: We are confident of achieving our target numbers because of our comprehensive recruitment strategy (as described at the start of this section on Sampling), extensive networks, prior experience with the marginalised, and the experiences of other pandemic surveys including those of co-applicants. We also believe the emergence of long Covid critical mass and the particular desire of people with marginalised chronic conditions such as long Covid and complex (multisystemic) comorbidity (such as Ehlers Danlos Syndromes, Chronic Fatigue, Fibromyalgia) to contribute to research to get their voices heard, means there will be a strong appetite for our survey. Should we over- or under-sample, we will use all the data; under-sampling may preclude within-area analyses. There are two particular risks that require mitigation:

1. Should we fail to achieve even 3,200 at wave 1, we will reduce the study design to a two-wave survey, leaving recruitment open for longer at each wave. In the worst case, we will only undertake one wave. However, we consider these mitigation scenarios highly unlikely from initial scoping and from past experience of team members for other surveys.
2. Failure to recruit enough minority ethnic participants - the above shows the minimum number needed is 1,600 BAME respondents. Several team members have considerable experience in recruiting ethnic minorities with chronic health problems specifically (Goff; Bromley-by-Bow Centre; Dickerson, Programme Manager of Born in Bradford/ActEarly) and we believe minimum numbers can be reached with a four nations survey. In Dickerson's successful localised 2020 BiB survey, only 18% of 2,144 respondents were White British. We have ensured strong connections also with ethnic minority organisations that deal specifically with chronic conditions, such as collaborators MedAct and via collaborator Abou-Saleh. So we believe we will be successful in our plans. However should this not be so, we will be able to undertake useful analyses of white British data and can then explore ethnic intersectionalities within WP3 and by collapsing ethnic minority +/- PwCC samples in the survey. See also our methods of dealing with **attrition** in the next paragraphs.

Survey attrition and missing data: We will require completion of almost every question on every page for participants to proceed, so we can undertake the association analyses required. This means there should generally be no *missing* items in any measures, though this requirement may lead to *completion attrition*, with respondents giving up and logging off. We will try to mitigate that with the questionnaire design which will be developed and piloted with our PPI group and N=30 others. There is the risk of *attrition between waves*. Participants will be asked to provide an email address on enrolling, if online. The RedCap online secure system will then automatically re-contact them for wave 2/3 follow-up questionnaires (with reminders) to explore trajectories over time. This automatic process makes for efficient and secure second and third wave recruitment to reduce the risk of missing respondents. Data will be anonymised prior to analysis and researchers will not directly handle email addresses; however they can control reminders. Careful design of the covering letter/page on between-wave reminders can improve return rates from those with lower levels of education or who speak languages other than English at home (69), so we have ensured these are designed with full PPI input. Lotteries appear effective in some online surveys (69) and we are including a £50 Amazon voucher as an incentive given at random. To handle missing data and address panel attrition and item non-response, we will use modern methods, including Full Information Maximum Likelihood, Multiple Imputation with Chained Equations that produce unbiased estimates under assumptions of missing at random (i.e. missingness depend on observable data only) and multivariate normality; and pattern mixture models that address missing not at random (i.e. missingness may also depend on unobserved data) assuming correct model specification (70). Those techniques, under certain assumptions, ameliorate loss of statistical power due to missing data and possible biases due to systematic missingness.

WP3 interview sampling: We aim for 210 interviews with purposive quota sampling (**Table 1**) for maximal diversity and sufficient numbers for rich data for each group, and recruitment through the same channels as in WP2. Posters, adverts and snowballing will target those who lack resources or technology to respond to online recruitment (62), for example via our clinical co-applicants and our collaborators. (Though many migrants or their local groups use digital technologies e.g. to contact 'home' (71), for pandemic faith meetings.) The 40-plus newly set-up long Covid specialist clinics will provide a further possibility of recruitment, for example via posters. Our sampling frame follows an intersectional studies approach that allows us to consider and compare assumed homogeneity across condition effects irrespective of ethnicity, and across ethnicity irrespective of condition, as a tool to tease out intersectional factors and heterogeneity. At analysis the focus may switch to other commonalities such as shared barriers or facilitators to health and social care resources.

Table 1: Interview sampling frame (cells contain numbers to be sampled)

Ethnic/national origin / ***Condition effect***	*Migrant: Middle East*	*Migrant: sub-Saharan Africa*	*Migrant: Poland*	*Migrant: India and Pakistan*	*2nd Gener-ation*	*White British*	*TOTAL*
Mental	5	5	5	5	5	5	**30**
Mobility	5	5	5	5	5	5	**30**
Stamina/breathing/ fatigue (incl. heart)	5	5	5	5	5	5	**30**
Hearing/Vision loss	5	5	5	5	5	5	**30**
Developmental/ intellectual	5	5	5	5	5	5	**30**
Dietary	5	5	5	5	5	5	**30**
No condition/ disability	5	5	5	5	5	5	**30**
TOTAL	**30**	**30**	**30**	**30**	**30**	**30**	**210**

Main interview inclusion criteria:

- White British comparators or Arab, Polish, Indian, Pakistani or sub-Saharan African 1st or 2nd generation refugees/migrant adults aged 18+ (undocumented, on temporary visas, indefinite leave to remain, British citizenship) (Table 1). While not homogenous, these groups were chosen to be diverse but focused enough to ensure rich data and on the basis that a) 74% of refugees resettled in the UK since 2010 were Arabs and Turks, 19% sub-Saharan Africans (72) (who are also the most likely to die from COVID-19 in the UK (16)), b) recent migrants by choice were mostly born in Poland or India (72) and c) the 2nd highest UK COVID-19 mortality rates by ethnicity are for people of Pakistani origin (16).
- Any condition/disability, including self-diagnosis, that chronically affects daily activities.

We include skilled migration, humanitarian or family streams, the 'irregular' or undocumented, on temporary visas or first-generation migrants with indefinite leave to remain, or first or second generations with British citizenship.

Exclusions: a) student migrants as likely to have structured educational institution support, and b) residents of detention centres/closed facilities linked to national migration policies (e.g. new asylum-seekers/refugees, displaced or trafficked persons), as complex cases with specific considerations.

Risks of non-recruitment and attrition: We aim for sufficient participants for rich data for all our main ethnicity/disability combinations shown in Table 1. Possible attrition (up to 20% based on BiB experience) between waves may require further recruitment if theme/pattern saturation is not reached. But if many combinations provide similar data, leading to saturation, we may stop recruitment early or modify our recruitment strategy for theoretical sampling. To reduce risks of non-recruitment and attrition, and to enable us to achieve our aims, we have considerable in-built capacity to do this work. Rivas previously managed a similar-sized interview study (73) over 12 months with three researchers interviewing 217 men with cancer remotely whilst working on other projects and parts of the study. At stage 1 she calculated resource requirements for the current study based on this experience but since then we have added further capacity in response to panel feedback.

We have two **full time qualitative researchers** to cover the interview and workshop periods of the study. Our PPI leads and qualitative postdoctoral researcher will train local **lay co-researchers at each site** as part of the core study design. Our named PPI representatives as co-applicants and collaborators have also been costed for possible interviewing co-researcher work. Additionally, **Bromley by Bow** will undertake direct recruitment and interviewing as **co-researchers**. Their involvement is significant as

they have direct experience in undertaking similar work with local migrant groups in the past, and access to suitable participants. This community group is confident of recruiting and interviewing the required sample and running the London workshops. It is run by lay people who belong to the communities of interest and is connected to an adjacent health centre. Likewise, Dickerson is Programme Manager for **Born in Bradford** and has direct access to suitable participants and co-researchers in this research-ready city collaboratory. PI Rivas and co-applicant Redclift will also do interviews if necessary; both have considerable expertise in this with migrant populations. All people undertaking interviews will be fully trained to ensure a consistent approach.

Risks for participant mental health/safeguarding: Abou-Saleh who undertakes migrant crisis assessments for the Helen Bamber Foundation will advise and we will provide clear signposting to sources of help. Immigration concerns need assured anonymity and sensitivity.

WP4 Key informant interviews: Recruited through our team, collaborator and Advisory Group networks and emails to local service providers and organisations.

SUMMARY OF PATIENTS/SERVICE USERS/PUBLIC AS RESEARCH PARTICIPANTS

Inclusion/exclusion criteria: We have taken care to involve a range of ethnicities and a full range of disabilities including long Covid and self-diagnoses (see p1 for full rationale). Our exclusions are based on relevance and safety considerations as advised when our study underwent ethics review. Dickerson, BiB Programme Manager, has taken care to ensure we will not sample the same groups as the BiB current COVID work to avoid research burden. Our PPI advisers suggested we only interview participants able to communicate in English so the focus would not be on language fluency, which is a specific issue the PI has previously studied (37). We will be inclusive of disabilities through responsive accessibility formats.

Recruitment/consent process: We do not use randomised sampling but recruit participants from adverts/links distributed through a range of platforms and networks (please see p11), as well as local lay co-researchers for our qualitative work (see p14). Potential participants will be informed that interviews will be in English, and it is their choice as to whether they feel able to take part. This is a well formulated and effective process in Born in Bradford. Where a participant is happy to interview in English, but feels more comfortable doing so in their home language, if a researcher fluent in that language is available this will be arranged.

Our consent process has ethics approval and will be in English as default. Translated study documents will be made available if required specifically to ensure fully informed consent. Our focus on specific groups makes this manageable. Braille and other formats e.g. for neurodiversity will be used if needed. **Participant information materials** have been ethics-approved and for interviews and workshops are in print/digital form. For surveys they precede survey responses and the consent form, online. They have been piloted within relevant communities. We include approaches to increase survey respondent retention (p13). Bromley by Bow collaborators are a community group involved in similar research already; they were initially contacted to support recruitment but offered to be co-researchers. They are fully costed in, bring considerable experience and are lay members of relevant communities.

Research methods and frequency of data capture: We have three waves of a 15-minute survey for each WP1 participant spread over the study. For each WP3 participant we have a 1-hour interview including network/map/photo elicitation methods, and two 2-hour remote research workshops (designed to minimise research burden) spread over the study. We have 5 stakeholder 2-hour co-create workshops with different participants and 15 x 1-hour key informant interviews.

Study participant support: We include lay co-researchers locally, partly because they will be sensitive to local situations and contexts, particularly relevant when the country is subdivided according to COVID risk, as well as cultural needs. We will ensure participants know they can drop out at any time without adverse consequences. Any participant mental ill health and distress will need advice from collaborator Abou-Saleh who undertakes migrant crisis assessments for the Helen Bamber Foundation and we provide signposting to sources of help. The ActEarly networks include service providers to whom participants could be referred if needed. As this study is planned to be fully remote, which supports access by people with chronic conditions/disabilities, our main concerns will be to match participant fatigue/wellness levels and to ensure frequent breaks. The PI has undertaken other pandemic projects and these have shown such measures ensure participants are not excluded. Consent and information documents may need to be prepared in specific formats such as special-coloured backgrounds and we will use online approaches such as Padlet or Miro only after ensuring they are accessible to participants; indeed these are likely to increase accessibility.

Deleted: But we will remain flexible to revisiting this during the study and have included translation plans through the UCL floating pool of temporary researchers if needed.

Deleted: received

Deleted: Local lay co-researchers will be chosen who can translate into relevant other languages as needed, with back-translation quality control checks.

Deleted: e people and with dyslexia

Deleted: also

Deleted: the

Deleted: also

Sharing study progress and findings with study participants: See p17.

Payments, rewards and recognition for study participants: Remuneration follows INVOLVE recommendations. Participants may be acknowledged by name on our websites but we will discuss the issues with them to make sure they are fully aware of these first. Our PPI team members will be invited to be co-authors on outputs and supported in their own outputs, e.g. for the supportive journal Research for All which is free to contribute to and read and is run from the PI's department.

WP3 SETTING

We use 5 sites in England for maximal sampling diversity in migrant population density, proportion of EU to non-EU migrants, and reasons for migration (**Table 2**) to ensure project findings are transferable across the UK. We will use WP2 4 nations survey findings to contextualise and evaluate transferability of WP3 findings. This is important as we sample in England only for qualitative work due to differences in the devolved nations in responses to the pandemic and in health and social care systems. While this means some of our findings may be more relevant to NHS England, we expect principles to be similar across the four nations and will consider this in our reporting and outputs. We will ensure that within our sites we recruit from a mix of local communities well served by immigrant-specific services, and less service-rich communities.

Table 2: Relevant features of chosen sites (72)

Features<br>***Site***	% of residents born abroad	non-EU % of all residents born abroad	Majority reason for coming
London	38%	68%	Work, asylum seekers, refugees
SE England, Canterbury	13.5%	58%	Work or to join family
Gatehead-Newcastle	13%	84%	Work or to join family
W Midlands, Birmingham	18%	75%	Work, also many to join family
Yorkshire, Leeds	10%	56%	Work and family

OUTPUTS, DISSEMINATION, AND ANTICIPATED IMPACT

Sharing study progress/findings with participants: will involve e.g. a) a website, b) regular newsletters, c) lay summaries in requested formats, d) infographics, e) webinars, f) a science café. All will be co-developed with our representative PPI group.

Wave outputs: Each wave of each WP will produce interim summaries of findings for different audiences (see next page) but also main outputs as below, for dissemination. The main platforms will be text and web-based. Recommendations and guidance will use an evidence to decision format (please see p11). More creative dissemination and outputs may use art, 3-D work, performance art or other relevant approaches if our co-design participants recommend it; similar award-winning work by co-A Thomson, a trained designer, is used internationally. Some may be developed as seed projects from the original funding.

WP1 main outputs are 2 review articles to inform health/social care policy and practice.

WP2 outputs (for each of the 3 waves including within and between wave and group comparisons):

1. ***A large database resource*** that focuses on PwCD and ethnic minorities and which we will share with other researchers or analysts e.g. via CLS
2. A unique development of a well characterised ***social networks module*** for our PwCD/ethnic minorities analyses
3. ***A summary of key mediators and moderators*** to target with service change outputs, focusing on assets, strengths, affordances
4. Group ***risk stratification*** using an evidence to decision format to inform preventative/care action.

WP3 outputs within each wave including within and between wave and group comparisons will be:

1. ***Co-created guidelines, recommendations, policy briefs, strategies and interventions*** that take account of what worked well/less well, assets, strengths and affordances, and touchpoints (where experiences might best be improved), to inform health and social care policy and practice, with ***evidence-based foci based on patterns in WP2 analyses***. For example, if medicines access is seen in WP2 to more strongly moderate physical health outcomes than other factors, and WP3 data suggest adaptations to practice that improve this, that might become a priority focus. A recommendation might

Deleted: , regardless of where the populations are based

Moved (insertion) [2]

Deleted: ***Planned outputs:*** WP4 matrix syntheses and narrative summaries will enable at-a-glance information, important for busy staff, and will also show gaps in evidence or areas of uncertainty (where different data diverge) as well as areas where change may be impactful. All outputs will be publicly accessible. Outputs will consider data on long Covid and other chronic condition/disability AND ethnic minority status, but also separate data for each subgroup, for optimal data usefulness.

Deleted: **Additional main**

Deleted: (months 0-3, updated months 14-17) will be

Deleted: oriented

Deleted: ing

Deleted: staff

Deleted: will be

Deleted: CLOSER

Deleted: of groups

be to improve medicines access through automated delivery systems, guidance might be to describe a model of good practice in medicines automated delivery systems, and an intervention might be the resources needed to set up such a system. These broader recommendations will include sub-recommendation consideration of mechanisms and groups as per our ***evidence to decision analysis described in WP4***. Our intersectional lens enables suggested strategies and interventions appropriate for intersecting structural, cultural and religious needs, levels of deprivation, ages, gender and other factors shown through our research to be relevant.

2. **Imagined alternative futures for policy and practice planning** for ethnic minorities with PwCD.

WP4 outputs:

1. ***A report on co-produced understanding of current and future implementation*** needs into policy and practice for relevant outputs developed in WP1-3 drawing on WP4 syntheses and narrative summaries.
2. ***Adapted training programmes, Tough Cookie and Pain Relief Management*** *and* a report on ***proof-of-concept work*** with these.
3. ***Adaptations to WP3 outputs suggested by WP4 work.***
4. A co-developed final *implementation, dissemination and rapid impact plan*.
5. A report on *data representativeness and transferability of findings*.
6. Web-based and printable at-a-glance information, important for busy audiences, as summaries and infographics. These will provide acti***onable findings in the evidence to decision format***, helping users ***prioritise where change may be impactful*** and showing gaps in evidence or areas of uncertainty (where data diverge). All outputs will be publicly accessible. The focus will be on the intersection of various conditions and ethnicity. Outputs will be *disaggregated* by sampled groups and key mediators and moderators where possible and appropriate.
7. ***Creative co-produced outputs*** e.g. as above (final nature to be determined in the study).

Key audiences: Team members are involved directly with relevant communities for assured impact and reach to key audiences, a list of which will be finalised in the co-design workshops and from our emerging data. But this is the anticipated range:

- health/social care practitioners, NHS/social care managers/policy decision-makers, through our clinical leads, WHO/UN (via Abou-Saleh), EPPI-Centre, Born-in-Bradford/ActEarly sites (via Bromley by Bow and Dickerson)
- the third sector, including many of our collaborators
- academics in disabilities, migrant, medical/health disciplines, social networks, participatory work
- PwCD/ethnic minorities communities, as represented by PPI co-applicants and collaborators
- community organisations such as collaborator Bromley by Bow, religious institutions
- COVID-19 research, policy and practice groups and networks, including the All-Party Parliamentary Group (APPG) on Coronavirus, long Covid networks, NICE long Covid committee.

DISSEMINATION OF OUTPUTS: Cascaded dissemination at each data wave, tailored to our key audiences, will emphasise practical solutions and implementation, and will be co-developed with key stakeholders representing our audiences.

All audiences:

- A dedicated accessible ***project website*** hosting twitter feed, other social media links, news items, and regularly updated findings/outputs/summaries suitable for different audiences, with accessibility formats. The website will be linked to university webpages and collaborator sites to increase visibility (e.g. in search rankings)
- ***Webinar*** presentation of study-end findings and implications, to reach broad audiences, accessibility-aware and supplemented by transcripts.
- ***blogs***, new and existing (e.g. Barts MS Research Blog with 4,000 views/day)
- ***YouTube videos, mass media interviews and press releases***
- ***Creative disseminations*** to be determined during the study, e.g. performance art.

Policymakers (see examples of policymakers in Connecting to policy, health, social care below): ***policy briefs, evidence summaries, guidance, recommendations***. The PI's Department has strong policy links and special interest in evidence for policy and practice (for example the PI teaches a course called Evidence for Policy and Practice, we have staff seconded to What Works Centres, and run the EPPI centre [Evidence for Policy and Practice Information Centre]), so active engagement will be a strong

Deleted: different

Deleted: ***Communication***

Deleted: communications

Deleted: and will be tailored to our key audiences, with

Deleted: ment

Deleted: of communications

Deleted: ***webinar***

focus, supported by UCL's Public Policy Unit e.g. ***through academic-policy roundtables, Arm's Length Body dissemination/meetings e.g. via ActEarly, EPPI pathways, Research Insights briefings.***
Academics: 2+ Sociology/Migrant Health journal ***articles*** (e.g. Sociology; Journal of Migrant Health), conference ***talks, knowledge exchange event, guidance, recommendations***:

- **Connecting to policy, health, social care practitioners** e.g. social workers, community health teams, clinicians, medical organisations, WHO: 2+ ***articles, talks, knowledge exchange event, training, guidance, recommendations, educational case studies*** on the implications of findings disseminated by e.g. Royal Colleges, ***practitioner journals***. To ensure engagement and action from shared outputs and disseminations, we will determine the best approaches through stakeholder analyses in our co-create workshops, and draw on existing networks and possibilities, e.g.:
  - We will contribute directly to the ***NHSE Optimum Clinical Pathways consultation*** of the Association of British Neurologists through Dobson.
  - ***Clinician co-applicants*** and especially Dickerson, Bromley by Bow, Saravanan and Abou-Saleh provide local and large-scale direct routes to practice (see 'Team').
  - We will produce information useful for the **national vaccine strategy.**
  - We will develop **aspirational action plans with social care staff** embedded at sites e.g. within Bromley-by-Bow and Born in Bradford, and more broadly.
- The EPPI-Centre will adopt the review in embedded ***Dept of Health and What Works Centres*** work, which provides evidence summaries for policymakers and practitioners.
- Adding findings to UCL's/QMUL's COVID-19 research databases and the EPPI COVID-19 Map of Evidence and other ***living maps*** will help ensure reach, impact and discoverability.
- UCL's Public Policy Centre will support ***policy impact***. The study will provide regular research reports and updates to the Cabinet Office, other government departments, Public Health England, the APPG on Coronavirus, SAGE/shadow-SAGE (we are networked with members), Arms-Length-Body meetings e.g. through ActEarly, EPPI networks and the World Health Organisation (e.g. through Abou-Saleh).
- We will aim to feed findings to the ***NICE committee on long Covid***. Rivas is a member of long Covid practice and policy networks including a facilitator of the CHAIN long Covid collaboration.

Advocacy groups/charities such as our collaborators**:** ***resources*** they can use to support guidance and recommendations provided to other audiences.
Connecting to patients/service users, carers, focal communities, the wider public: Our PPI team with lived experience, and other stakeholders such as third sector, clinicians, social care staff, policy staff (selection to be determined in consultation with our advisory group and PPI team at start of the study), will co-create outputs to ensure their credibility and real-world relevance and to strengthen public engagement. Being online, at least initially, widens participation opportunities. Please see the description of our co-create workshops for details, p11. All data used in these workshops will have all potentially identifying details excluded. An overview of early findings will be presented to participating communities more widely via collaborator platforms, to give them the ***opportunity to reflect upon and interrogate researchers' interpretations and analysis*** of the data and ideas for outputs. This will enable broader community input into the final project outputs such as ***empowering guidance, recommendations***. All findings will be publicly available via our ***website in accessible forms*** for lay consumption using recommendations in the Patient Engagement Open forum (https://bit.ly/388SFr0) and by ***involving trusted community channels***, such as places of worship, trusted religious leaders, community champions - possibly tapping into the infrastructure developed from COVID vaccine rollout - and community groups, including collaborators Bromley-by-Bow. This aligns with Black community comments in a meeting about vaccine uptake and UK government 2021 vaccine hesitancy guidance by the Scientific Advisory Group for Emergencies ethnicity sub-group (SAGE) (https://bit.ly/38GLt6D).
OUR IMPACT AIM: We will develop policy and practice impact outputs that lead to easily adopted changes to mitigate inequities in and improve the health and social support and wellbeing of our focal group in the pandemic, pandemic recovery, its aftermath and in case of future waves and similar events. For examples of outputs please see the explanation on p15. A relevant change might be to new digital models for care that require more than one specialist to be in attendance e.g. so a local team can get complex case studies advice from a tertiary centre or a multidisciplinary team (a possibility suggested by PPI lead Camaradou). Thus we seek as **impact subaims** to:

Deleted: 2+ ***articles, talks, knowledge exchange event, training, guidance, recommendations, educational case studies*** on the implications of findings disseminated by e.g. Royal Colleges.

Moved (insertion) [1]

Moved up [2]: ***Sharing study progress/findings with participants:*** will involve e.g. a) a website, b) regular newsletters, c) lay summaries in requested formats, d) infographics, e) webinars, f) a science café. All will be co-developed with our representative PPI group.¶

Moved up [1]: ***Connecting to policy, health, social care:*** To ensure engagement and action from shared outputs and disseminations, we will determine the best approaches through stakeholder analyses in our co-create workshops, and draw on existing networks and possibilities:¶
¶
We will contribute directly to the NHSE Optimum Clinical Pathways consultation of the Association of British Neurologists through Dobson. ¶
Clinician co-applicants and especially Dickerson, Bromley by Bow, Saravanan and Abou-Saleh provide local and large-scale direct routes to practice (see 'Team') ¶
The EPPI-Centre will adopt the review in embedded Dept of Health and What Works Centres work, which provide evidence summaries for policymakers and practitioners.¶
Adding findings to UCL's/QMUL's COVID-19 research databases and the EPPI COVID-19 Map of Evidence and other living maps will help ensure reach, impact and discoverability. ¶
UCL's Public Policy Centre will support policy impact. The study will provide regular research reports and updates to the Cabinet Office, other government departments, Public Health England, the APPG on Coronavirus, SAGE/shadow-SAGE (we are networked with members), Arms-Length-Body meetings e.g. through ActEarly, EPPI networks and the World Health Organisation (e.g. through Abou-Saleh).¶
We will aim to feed findings to the NICE committee on long Covid. Rivas is a member of long Covid practice and policy networks including a facilitator of the CHAIN long Covid collaboration.¶

1. provide information, guidelines and recommendations, strategies and interventions to help health and social care policymakers and practitioners to understand and address health and social care and support needs, access to resources, relevant assets and strengths, and vaccine uptake.
2. provide primary and secondary healthcare staff with personal stories of lived experience and the challenges that need to be addressed.
3. inform and empower service users through information in the public domain
4. inform further research through our findings
5. undertake relevant community and practitioner training, guidance and aspirational action plan development across health system levels (e.g. primary care, community clinics, secondary care, community-based rehabilitation and support services), initially with local impact at our 5 sites, but in the longer term with upscaling.

Deleted: and

The charity Patient Safety's list of what is needed to achieve change in health and social care for long Covid sufferers is applicable across our participants and resembles our impact sub-aims. This demonstrates how our vision coincides with patients'/service users'. This charity also suggests the need to inform wider society to influence people's attitudes and responses, including employers, the benefits system, advice services (e.g. Citizen's Advice, Healthwatch, Patients Association). We will target some recommendations to these. Please see **Figure 1** for shorter and longer term impacts.

Deleted: for reforms and guidance

FURTHER FUNDING OR SUPPORT NEEDS:

We have designed this study as stand-alone; some outputs can be used at once, but some intervention suggestions would need proof-of-concept/feasibility testing and trialling. We will adapt two existing training programmes, using our new evidence, and test them during the study at the 5 WP3 sites for proof-of-concept. These would likewise need to be properly evaluated for effectiveness/efficacy.

FURTHER RESEARCH, DEVELOPMENT, ADOPTION AND IMPLEMENTATION AND IMPACT

- This is an assets/strengths-based study, which draws on existing good practice and affordances. This means relatively small changes may be made that lead to big improvements in outcome, something our WP2 work in particular should elucidate. Thus there are relatively few barriers to their development, adoption and implementation and impact. We realised when developing the study that clinicians will be unable to make major changes to their work, more so in the pandemic than ever, when resources are particularly constrained, so this was an important consideration.
- Likewise our co-production work should ensure acceptability across stakeholders, accessibility and feasibility and this is an important focus of our work.
- Our clinical co-applicants and collaborators (Dobson, Ball, Eccles, Saravanan, Abou-Saleh) will be able to adopt our recommendations and simple intervention suggestions locally, which may lead to further interest, case analyses, PDSA evaluations and other considerations and thence large-scale evaluation/upscaling for longer term impacts.
- We will be able to impact Clinical Pathways guidelines through these clinicians, supporting adoption and implementation of findings.
- ActEarly sites will be able to adopt outputs, namely East London (represented by Bromley by Bow) and Born in Bradford (represented by Dickerson).
- We will adapt 2 existing community/lay-led training programmes, and provide one course of each at the 5 WP3 sites (and consider new programmes). Further use will require commissioning.
- Policymakers have little time to assimilate information at the best of times and the rapidly changing nature of the pandemic is especially challenging. Hence we will take care to produce the type of evidence they need, including infographics, quantitative summaries and targeted qualitative approaches that link to themes relevant to policy.

PROJECT MANAGEMENT:

Moved down [3]: **Project / research timetable**

Monthly project management co-applicant/collaborator group meetings will be held remotely, and 4-6-monthly remote PPI and advisory group meetings involving key stakeholders, an ethnic minority clinician from the General Medical Council's Black and Minority Ethnic Doctors Forum, a co-production expert, a patient who is not in the main PPI team (tbc), a covid clinical expert, a multisystemic conditions clinical expert, and a central policymaker. We will have a 12-month independent steering committee meeting. Co-applicants/collaborators will also touch base after co-create workshops to manage outputs and impact. Microsoft Teams will be used between meetings; in the context of pandemic-related online working this is the most efficient way of managing communications as members get alerted to new content, content can be organised in channels, and communications do not get lost in a forest of emails. It also means communication is 'always on', useful in critical moments and enabling easy phone

connectivity. Weekly or as needed meetings will be held between the PI, current WP lead(s) and researchers. Lay co-researchers will be able to contact the team as and when they need, and PPI leads will determine their support and contact for study management once the study begins. The PI will be responsible for conduct of the study, day-to-day management, decision-making. But all staff will share the same duty of care to prevent unauthorised disclosure of personal information and follow good governance. A data management plan will be developed before start of the study.

PROJECT / RESEARCH TIMETABLE

Moved (insertion) [3]

	Study month									
**Task**	Pre-study	1-2	3-4	5-6	7-8	9-10	11-12	13-14	15-16	17-18
Ethics and approvals										
Set up staff										
Develop and set up survey on Redcap										
Secondary text data analysis		1-	3					14	-	17
Secondary cohort data analysis		1-	3					14	-	17
Send out survey waves and complete descriptive analyses		2-	3		8-	9			15 -16	
Three waves of qualitative research and basic analysis of these			3-4			9-10			15 -16	
Co-design workshops										
In depth qualitative analysis (discourse, narrative)			3			-				17
Statistical modelling and social network analysis		2	-	5		9	-11		15 -	17
Key informant interviews and proof-of-concept training programmes									16	-17
Stakeholder dissemination and interim outputs for public, policy, practice										
Launch and final event										
Writing reports and papers										
Advisory group meetings										

ETHICS / REGULATORY APPROVALS

All participants will be informed about the study using a plain English statement, read to them if needed. PPI work suggested restricting interviews to English would not reduce the impact or usefulness of the study. Most research work undertaken by collaborators MedAct is in English. Moreover they suggest our research will usefully determine barriers for those who might be assumed 'OK' because they are not housed in accommodation for the vulnerable and can communicate in English. If we find this excludes intended participants, we will involve interpreters. Translated study documents/accessibility formats will be made available if needed to ensure fully informed consent.

For team, collaborator and participant safety, all activities will conform to any pandemic restriction measures in force at the time. Participants determined by the research team to be in a current crisis or situation that could be aggravated by involvement will be excluded for safety; with expertise from collaborator Prof Abou-Saleh, who undertakes migrant crisis assessments for the Helen Bamber Foundation. Our advisory group will help ensure interview and survey questions minimise participant distress, to which we will be sensitive. The core team will receive training in trauma-informed work through the KCL Violence, Abuse and Mental Health network to which the PI belongs, as many migrants have experienced considerable trauma in their lives. The survey, information sheets and interviews will all incorporate signposting to sources of help, and other resources. We avoid complex cases using exclusion criteria. Remote working has advantages with England-wide interviews, but reduces the sensitivities of face-to-face contact, which will be borne in mind. We will ensure lay researchers are well supported, with training in good governance, and that local gatekeepers do not cherry pick or exclude participants. We will pilot the interview topic guide with up to 10 people who fit the target group, using cognitive walkthrough (remotely). All sessions with participants will include a rest at 30 min and pauses as needed. Participants will be offered email as an alternative to oral sessions or to add to contributions. We use opt-in contact. Migrant status and ethnicity are protected characteristics so the study will be run via RedCap in UCL's Data Safe Haven. Anonymity and confidentiality will be stressed and carefully upheld including in photovoice work; undocumented migrants will fear arrest and deportation. We have provisional UCL ethics approval (REC 1372). All data will be held and handled in strict accordance with UCL Ethics, the UK Data Protection Act 2018, UCL data protection policies and GDPR. The lawful basis for processing personal data for GDPR will be 'public task'. The PI conducted a Data Privacy Impact Assessment (DCIA) and registered the study for this. Data will be fully anonymised, removing direct identifiers (e.g. names). No information will be released/disseminated that could lead to identification of participants including in qualitative data extracts. In line with UCL policy, electronic research data will be stored on the data server for 5 years and subsequently on external hard drives for 10 years,

converted to suitable open formats for long term preservation. Anonymised interview data will be available for secondary analyses according to the prevailing requirements of the UCL Data Safe Haven at the time requests are made.

PROJECT / RESEARCH EXPERTISE

Our team reflects the need for expertise, sensitivities and networks across ethnic/migrant groups who may otherwise be reluctant to engage, and for good pathways to policy and practice change. Some team members have personal experience of disabilities; others will enable critical distance. We have divided key contributions into specific areas below.

Methodology: PI Rivas has strong expertise across the proposed research and strong relevant networks. She was NIHR RDS London-wide qualitative methods lead 2012-2014, and has led remote qualitative pandemic research (please see her cv). She has strong expertise in impactful systematic review work (please see her cv). She works with the EPPI-Centre, with its strong links to Arms-Length Bodies. Aksoy is expert in social network analysis, and Nasim in longitudinal surveys. Thomson has a strong record of influence on policy and practice through prize-winning knowledge-exchange and co-design expertise and strong links to patient groups and charities.

Migrants: Redclift brings migrant research experience and links to Canterbury migrant groups and the Runnymede Trust. Collaborator Abou-Saleh will contribute his expertise and influence as past/current executive of WHO Biological Psychiatry, the Syrian Association for Mental Health (Syrian Refugees), Helen Bamber Foundation for assessment of refugees and asylum seekers and the Refugee Council, London. Other expertise comes from the third sector (see below).

Practitioner education: PPI member Gaulbert is a long Covid sufferer of Indian heritage and a **pain relief** therapist. She trains multidisciplinary clinicians in how to utilise pain research findings and incorporate them into their practice and will extend this within the study using our findings. We have costed in 5 training sessions to be undertaken at each of our 5 sites, or online with a capacity of 50 professionals per session. **Tough Cookie** is a Mental Resilience training programme and we will adapt this using findings, to use with community members, i.e. PwcD from ethnic minority groups.

Clinical pathways: Clinical co-applicants are well-placed not only to introduce findings into their own practice, which will be considered during our implementation pathway work e.g. in co-create workshops, but to also influence guidelines and policy. Dobson is Clinical Lead for Neurology at Barts Health, so a direct influencer of local policy and practice. Ball has a front-line leadership role with the Royal College of Obstetricians and Gynaecologists. Eccles has vast clinical expertise in chronic pain and fatigue, and strong influence on liaison psychiatry services as well as with opinion leaders in rheumatology. Saravanan is a Consultant rheumatologist with interest in hypermobility and involved in setting up one of the 40+ new long Covid clinics, in Gateshead, which provides an important route to impact. He holds North East CRN funded time for research which means he has not been costed for this study, adding to its value. Lay co-applicant Camaradou advises on a separate rehabilitation clinics project that could enable future collaboration. Bromley by Bow community group collaborators are directly connected with a transformative health centre, within the ActEarly London collaboratory. Collaborators MedAct Migrants are a group of activist doctors specialising in migrant clinical support.

Commissioning, policy, other influence: Goff has worked with the CI before, on a participatory co-developed culturally tailored diabetes education programme for those of African descent, with 2 prizes (please see the PI's cv). This is currently being rolled out in the NHS and Goff has strong links with commissioners. Diabetes is a condition of particular interest as a recognised risk factor for COVID-19 severity, a condition that may develop de novo within the long Covid complex and a condition the self-management of which could be compromised by long Covid-disrupted smell/taste (74). Lay co-applicant Parsons previously worked within NHS Southwark and Tower Hamlets Clinical Commissioning Groups, in the former as Programme Manager, and brings relevant expertise. Dickerson is Programme Manager of the UKRI ActEarly 'city collaboratory', a population-based, system-wide infrastructure for implementation in Bradford and East London; Bromley by Bow community group collaborators are also partners in ActEarly. This provides us with considerable direct access and influence e.g. Dickerson works closely with Bradford, regional and national policy and decision makers in health, education, environment and social care (75). as well as with key stakeholders nationally (including Public Health England, Department for Education and Schools, Association of Directors of Public Health) and regionally (West Yorkshire Health and Care Partnership, and Yorkshire and Humber Applied Research Collaboration). The London half of the ActEarly consortium brings similar links in the capital that we can access. Camaradou is part of the global COVID-19 END evidence synthesis and has 15 years' commercial experience in policy, stakeholder engagement partnerships, project management and

innovation R&D commercialisation roles across public and private sectors. Redclift is an academic advisor with the Runnymede Trust, the UK's leading independent race equality think tank.
Lived experience, associated networks: We have strong patient representation. Camaradou (migrant, chronic disabilities, long Covid), Kumar (immobility, vision disabilities) and collaborator Ahmed (endometriosis) are ethnic minority women with disabilities and strong third sector London networks and lead on PPI. We also have Parsons (long Covid), Gaulbert (Indian heritage, long Covid, chronic pain), Sandhu (Indian heritage, long Covid), Gabriel (young black male with long Covid). PI Rivas is a 2nd-generation Polish/Ukrainian migrant with relevant connections, and two disabled children. Aksoy, Nasim and Saravanan are from minority ethnic groups.
Third sector for nationwide recruitment and dissemination support: Collaborators include national and local migrant and disability groups, including representation from the most significant or prevalent UK chronic health conditions/disabilities, with whom team members have established strong networks. While most are third sector and impacted by the pandemic, their support will mostly involve advertisements and calls for participants on their websites and in the materials they usually distribute, so they have assured us of capacity. Existing networks e.g. Autistica, Multiple Sclerosis Society, Ehlers-Danlos Support UK, Fibromyalgia Action UK, Diabetes UK, British Psycho-oncology Soc, Endometriosis UK, British Lung Foundation-Asthma UK **have all provided letters of support**. We will also contact MIND, RNIB, Deaf Society UK, Versus Arthritis, MND Association, Beat, Parkinson's Society, Scope, Alzheimer's UK, Epilepsy Society, British Heart Foundation. Local organisations at sites (e.g. Kent Refugee Help). MedAct Migrant Subgroup is costed in for recruitment support.
Researchers: Nasim and Oksay will line manage one 18-month 100%FTE quantitative researcher and Rivas will line manage two 100%FTE qualitative researchers, one for 18 months, one for 10 months, all based at UCL. See Justification of costs in the main form for training/support details.

CICADA-ME: Coronavirus Intersectionalities: Chronic Conditions and Disabilities and Migrants and other Ethnic minorities

SCIENTIFIC SUMMARY

Two groups that experience similar societal inequities (expanded by the COVID-19 pandemic), including in social and health care, are people with chronic conditions/disabilities (PwCD) and ethnic minorities. The worst affected are both ethnic minority AND with chronic conditions/disabilities, a common group, as COVID-19 mortality statistics show. There is a largely unmet expressed need to explore this combined group's pandemic experiences with new or worsening conditions/disabilities including post-Covid syndrome in relation to reduced services, inequalities, lifestyle changes or health neglect and vaccine uptake. We aim to contribute and inform evidence-based formal and informal strategies, guidelines, recommendations and easily adopted interventions for pandemic-related and future health and social care policy and practice, to mitigate inequities and improve the experiences, health and wellbeing outcomes of minority ethnic groups at the intersection with chronic conditions/disabilities. To do so, we will develop a rich intersectional understanding of their mental and physical health, coping, access to resources, and informal and formal social and health care support experiences, and relevant assets and strengths, longitudinally over 18 months using mixed methods. Our 4 work packages involve a new UK survey in 3 waves, parallel qualitative insights, secondary analyses of other surveys, rapid review, and outputs for immediate use developed with participatory methods, with co-create workshops involving our PPI team and other stakeholders throughout. Our survey (n=5000) samples for 1st and 2nd generation community-dwelling minority ethnic groups and white British comparators, all with/without chronic conditions/disabilities, across the UK's 4 nations, to determine relationships between measured variables and their trajectories. After Survey Wave 1 we will interview 1st and 2nd generation ethnic minorities from Poland, India and Pakistan, sub-Saharan Africa, and the Middle East, and white British comparisons, all with and without chronic conditions/disabilities, about their pandemic experiences at 5 diverse sites in England, supporting transferability. Interviews (n=210) informed by survey analyses will include social network analysis, photovoice. We will train local lay people to help undertake these remotely; a transformative community migrant-majority research-active group will be our main London co-researcher. This group and a main co-applicant are members of an existing UKRI consortium and have undertaken complementary work at two of our sites, demonstrating the feasibility of our plans. At Waves 2 and 3 research workshops with interviewees will use video vignettes built from earlier study findings. Key informant interviews and co-create workshops will consider implementation. We will synthesise Keyword frequency analysis, Framework, discourse and narrative analyses, Latent Growth Modelling, Structural Equation Modelling, and social network analyses using tabulated evidence to decision methods, with interim findings reported at each wave for early delivery of benefits. Respondent and national UK demographic data will be compared for representativeness, and transferability explored at each stage. Data will be presented separately and combined for ethnic minorities and PwCD. We expect rapid impact from our strong networks and Co-A and collaborator existing strong pathways to influence in health and social policy and clinical practice.

BACKGROUND AND RATIONALE

The problem: The greater risks and challenges faced by two vulnerable groups during the COVID-19 pandemic, ethnic minorities and those with underlying health conditions/disabilities (1-8) are now well recognised. Although disabled people constitute 16% of the population, they represent 59% of all COVID deaths (9). Similarly, though 13% of the UK population, 33% of critically ill COVID-19 patients are from non-white ethnic groups (6,7). One reason is the intersection of minority ethnic status or chronic poor health or disability with other inequities (please see **Box 1**), which persisted before the pandemic and have widened because of it. Our particular interest is in improving pandemic and longer-term networks of support and access to care, services and resources for these vulnerable populations (1-5) to enhance vaccination, social, health and wellbeing outcomes.

The need to foreground intersections of chronic condition/disability AND ethnicity: Notably, the pandemic has highlighted how ethnic minority and poor health/disability statuses themselves intersect (1-5,8), with calls for research on this (e.g. BMJ) (10). **First**, having both chronic poor health/disability and ethnic minority status is associated with worse health than belonging to just one of these groups, even outside the pandemic, as noted for resettled refugees particularly (8,11). The emergence of post Covid syndrome, or long Covid (12,13), with a 5-week prevalence of 20% (14) has highlighted some of the issues. Anecdotal evidence suggests long Covid accounts may be more often ignored when made by people from ethnic minorities or who have a similar pre-existing disability such as complex

multisystemic conditions. **Second**, chronic conditions such as diabetes and cardiovascular disease are disproportionately common in some ethnic minority groups (15) - one reason for their increased risk of serious illness or death from COVID-19 (16). Considering mental health, the estimated 2% of the population who are recent refugees or undocumented migrants (11) had a considerably higher pre-pandemic prevalence of PTSD and depression than any other group (17) and minority ethnic groups report markedly poor pandemic mental health (18). To improve support and care for these vulnerable people, it is critical that we specifically consider the intersection of chronic conditions/disabilities (including long Covid) WITH ethnic minority status. This is the basis of our study. We will consider health and social care and support experiences across a range of combinations of chronic condition/disability and ethnicity. As **Box 1** shows, we cannot consider these intersections in isolation, though at the core of our study, so we also explore other categories of societal difference (e.g. age, gender) that interact with health status and ethnicity under institutional and structural conditions to create specific health outcomes and experiences (19). In particular we foreground citizenship status as influencing the support available to ethnic minorities, since many recent refugees and undocumented migrants will have 'no recourse' to welfare and housing support. Underpinning our study with intersectionality theory allows for complex nuanced insights into differences, while minimising the risks of a) essentialising some combinations as inherently problematic or b) considering the ethnicity/migrant experience as homogenous. In this application we refer to People with Chronic conditions or Disabilities (which we shorten to PwCD) AND ethnic minority status (encompassing a range of citizen states) as our focal group. But our study is designed to also be independently applicable to those with long Covid, chronic condition/disability, or ethnic minority status and in our outputs we will disaggregate these data.

The need for our particular study design: Critically many of the pandemic health and wellbeing challenges faced by our focal group can be mitigated by small adjustments to health and social care service policy and delivery, formal networks such as community health services and informal networks such as family and friends (11). Yet this is not done; the voices of PwCD or ethnic minorities rarely feature in pandemic planning (4), are not reflected in vaccine roll-out, and there is a remarkable lack of primary data. Public Health England has called for this to be addressed in the next stages of the pandemic through **participatory research** (20), which we will use alongside rapid review, surveys and interviews in a design involving strong PPI contribution throughout that makes our work relevant for ethnic minorities and PwCD separately and combined. Our **social network analysis** is key. An understanding of appropriate networks is vital to improving access to health/social care and support, resilience to stress and post-disaster recovery (19,21), and informing interventions based on health-related behaviours and health beliefs e.g. misinformation in the pandemic and vaccine uptake/hesitancy. We will explore how knowledge about network use may be harnessed to improve pandemic-related experience. Our study has a strong **practical focus**, important given our aim for immediate impact; it will use a **strength and assets-based mixed methods approach** to probe for resourcefulness and successful strategies/interventions used since the start of the pandemic. It includes consideration through the study of **new service delivery models** with continued use and advantage beyond the pandemic (e.g. telemedicine [22,23]). Importantly our study is **longitudinal**. Thus we will be able to explore significant relationships in the survey data we collect on mental and physical health, coping, access to resources, social and health care support, vaccine uptake and intersectional variables and also change in these over time and with varying pandemic contexts. The qualitative data will provide rich detail in what is currently uncharted terrain. We will be able to track trajectories of long Covid; international opinion is that its relapsing-remitting nature requires this (24).

Box 1 Inequities for ethnic minorities (including migrants) and those with chronic conditions/disabilities increasing their risk of poor pandemic health outcomes (2,3,11)

1. **Increased risk of isolation, abuse or neglect**, **poor access to informal emotional and wellbeing support**, due e.g. to national pandemic responses, stigma, changed activities, priorities, attitudes of others, a state of 'normalized absence, pathologized presence' (25).
2. **Inequitable formal treatment, support and care** from attitudinal, structural, policy, cultural, linguistic, communication and economic barriers, leading e.g. to difficulties implementing recommended COVID-19 avoidance strategies, vaccine mistrust, and risk of severe illness.
3. **Psychosocial factors raising COVID-19 risks, reducing capacity to cope** with social, economic and psychological pandemic impacts, including worries about people 'back home'.
4. **Unemployment/reduced income** (e.g. zero hour contracts; 'no recourse' to welfare).

Terms used: For simplicity, despite its problems, after PPI discussion, we use the term **migrant** for someone born outside the UK (as per UK policy) who intends to stay in the UK for 1+ years, including asylum seekers and refugees. We use '**ethnic minority'** to encompass migrants and 2nd generation ethnic minorities (i.e. UK born). We include any **condition/disability**, including self-diagnosis, that chronically affects daily activities (e.g. diabetes, dyslexia, chronic pain, loss of limb, depression, autism). We will record conditions, but group by Dietary and 5 UK Family Resources Survey themes: Mental, Mobility, Stamina/breathing/fatigue, Hearing/Vision loss, Developmental/intellectual. We include long Covid and other multisystemic conditions (which may belong to more than one group); our categorisation by impact not diagnosis enables a practical focus and a flexibility to changing understandings of long Covid. **Long Covid** is defined by NICE (26) as: "*Signs and symptoms that develop during or following an infection consistent with COVID-19, continue for more than 12 weeks and are not explained by an alternative diagnosis. It usually presents with clusters of symptoms* [this may be more than 4 hence the Covid Symptom Study (https://covid.joinzoe.com/blog) under-reports long Covid]*, often overlapping, which can fluctuate and change over time and can affect any system in the body."* According to the UCL/Oxford symptom survey (28) the most common ongoing symptoms in 201 long Covid patients (only 18% were hospitalised), were fatigue (98%), muscle ache (88%), shortness of breath (87%), and headache (83%), according with other long Covid studies.

EXISTING LITERATURE AND STUDIES

The impacts of COVID-19 on the lived experiences and services access of ethnic minorities or PwCD, at the time we submitted our stage 1 application, were only considered within published papers in commentaries and calls for action. The evidence base remains much smaller than the overall COVID evidence base despite the greater impact of COVID-19 on these groups.

Ethnic minorities: A simple scoping search of the more than 30,000 **articles** in the EPPI COVID living map of evidence (https://bit.ly/3npqGtz) for 'ethnic*' or 'minority' or 'minority ethnicities" or 'black' or 'BAME' retrieved 67 hits, with only two directly relevant to the proposed study (in our first work package we will extend our literature search to increase its sensitivity). Both were US surveys. One, surveying ethnic minority asthma patients and doctors, reported socioeconomic factors and institutional racism impacted on asthma care in the pandemic; 25% of doctors found it more challenging to care for black patients with asthma during COVID-19 (28). The other showed pandemic telehealth was most used by black patients, attributed to their need to compensate for prior health and health care disparities caused by systemic racism (29). Both studies therefore support the need for our study. Given the rapid development of COVID-19 research it is important to also consider where our study fits with **ongoing studies**. As of December 2020, none of 248 studies on the NIHR site 'Be part of research' specifically considers the health/social support experiences of ethnic minority groups. Among jointly funded UKRI-NIHR studies, none has our focus on producing practical strategies and modifications to existing support and care that can be immediately implemented with minimal/low cost and effort, though several consider alternative aspects of the ethnic minority experience such as COVID-19 infections or migrant working, or engagement with pandemic information. **Outside of the pandemic**, the few studies (e.g.30-35) of the post-resettlement lived experience of recent migrants to the UK have a different focus/intent and no formal social network analyses. There are more studies of 'cultural competence' in healthcare (e.g. by team members, 36-37) but though their findings support the need for this study, they tend to a narrow focus on settled single ethnic groups with one specific condition (and one of the condition/disability impacts we consider) and do not transfer to the current situation or cover conditions similar to long Covid. This shows how our work is important and will provide much needed information that could be extended to and tested in non-pandemic situations in the future.

Chronic conditions or disabilities**:** Our EPPI-map scoping search found several 100 **articles** using the search terms 'disability' or 'condition' or 'illness' (to be extended in work package 1) but most were survey or audit-based considerations of reduced non-COVID patient footfall. In a global COVID-19 survey, 17% of 548 respondent rheumatologists estimated 25% of their patients had no access to telehealth (38) showing the need for alternative strategies such as we aim to explore. Interviews with 7 disability NGO representatives in Italy highlighted bureaucratic challenges, and a lack of advice, coordinated care plans and inter-agency coordination to compensate for reduced services (39). Small COVID surveys inside and outside the UK have shown the negative impact of reduced access to treatment on patients' symptomatic control, for Parkinson's Disease (40), migraine (41), rheumatology (42) and chronic refractory neuropathic pain and their increased reliance on support networks (43). Our study goes beyond these studies of impact, to focus on strengths, assets and solutions to issues. For example, small cross-sectional analyses suggest some chronic conditions and disabilities may confer

resilience to mental health or wellbeing effects of the pandemic (44,45) while a UK pandemic analysis of chronic fatigue Reddit posts reported more severe symptoms in some people but also more accessible opportunities to interact (i.e. online videocalls) (46). In the UCL UK COVID-19 Social Survey (18), which explores psychosocial health in the whole population and so has a different focus to us, 38.2% of 51,417 analysed respondents have pre-existing physical conditions (with a much narrower definition than ours), 19.9% pre-existing mental health conditions and 12% are from 'BAME' groups. This study found good support and resource access protective for pandemic mental health which has informed our study design. Thus the limited evidence supports our study and there remains the need for the insights, consideration of multiple intersecting factors and outcomes and particularly generation of solutions, such as we aim to provide. We found no relevant **studies** on the NIHR site 'Be part of research'. Considering other studies, including jointly funded UKRI-NIHR studies, three are most relevant. One considers the response of organisations who provide services for refugees and asylum-seekers (through 20 interviews) against the lived experiences of the people they support (40 interviews), in Scotland and Newcastle-Gateshead, combined with a UK wide two-wave survey specifically targeting asylum-seekers and asylum services (Hopkins, Newcastle). This focus is slightly different to ours. Shakespeare (LSHTM) is conducting 60 in-depth telephone interviews with a range of disabled people, including parents of disabled children, with different conditions, across England and Scotland, as well as with 15 key informants, repeated at six months. This study, smaller than ours and single-method, has a different sampling frame and limited ability to consider intersectionalities. There are studies of people with intellectual disabilities (Hastings and Hatton at Warwick and Lancaster) or with dementia and their carers (Banerjee in Bristol plans over 250 telephone interviews and Clare in Exeter up to 700); these groups require specific considerations in study design and the consent process so we do not target them in recruitment, though we do not exclude them. A qualitative study (McHale, Birmingham) considers impacts of the legal suspension by local authorities of the application of certain provisions under the Care Act 2014 as part of COVID-19 emergency powers. Several small-scale studies by support groups or local clinics are in progress to consider long Covid lived experiences. In none of these studies is there a focus on assets and solutions. None considers the combination of chronic condition/disability AND minority ethnicity. All are complementary to our own study.

EVIDENCE EXPLAINING WHY THIS RESEARCH IS NEEDED NOW

As we show above, our work addresses a still largely unmet expressed need in pandemic research to focus on PwCD and ethnic minorities pandemic experiences. Over our study period there will be a need for more focus on 3 specific areas of chronic health and disability, all more critical in our focal group:

1. New conditions/disabilities that develop or old ones that worsen because of reduced services and other structural consequences of the pandemic (which have widened pre-existing inequities for PwCD and minority ethnicities) (10)
2. New conditions/disabilities that develop or old ones that worsen because of lifestyle changes or neglect of health during the pandemic or pandemic responses such as shielding
3. Long Covid as an emergent chronic condition.

The greater impact of the pandemic on PwCD and ethnic minorities is a human tragedy and care issue (1), costly at personal, community, healthcare, economic, societal and human rights levels. The impact is likely to increase through our study across these 3 areas, given the negative impacts on population health. Our community-based mixed methods longitudinal approach is designed to consider this and inform and shape the immediate and future health and social care response particularly for ethnic minorities with chronic conditions/disability, and to take account of future pandemic impact and uncertainty. We believe there is considerable synergy and learning potential to considering both existing chronic conditions/disabilities and **long Covid,** since symptoms of long Covid and some existing conditions correspond and an underlying mechanism-in-common may be MCAS (47) (though there are alternative explanations (1)). In this regard our study is both unique and particularly important now for future planning considerations; the lessons we can learn from existing conditions are likely to be transferable to people with long Covid and their health and social care and vice versa. Policymakers such as those within Public Health England (PHE), and practitioners such as clinicians and social support workers, lack but need the evidence we will provide on PwCD and ethnic minorities for equitable care, as the statistics at start of this application make clear. PHE specifies an urgent need for participatory work with minority ethnic groups (13) such as will undertake. With 3 waves of data collection, we can report interim findings at 3, 9 and 16 months for rapid impact. Given the rapidly changing nature of the situation and the considerable impact that care issues are having on the nation's health and wellbeing, it is important for policymakers and practitioners to have timely contemporary

evidence in this way that they can act on quickly. Our work on both health and social care across multiple levels (see Theoretical framework p6) fits well with the new NHS plans for integrated care with system-working across NHS providers, primary care, local government, social care and the voluntary sector (48). Notably we also consider vaccine uptake. Respondent and national UK demographic data will be compared for representativeness and transferability explored at each stage. Our co-create and advisory groups – currently planned as remote work - will explore how to translate findings at each wave into practice in the 'real world' to ensure implementable, relevant, rapid, useful policy and practice underpinned by implementation science (49). We also expect rapid impact from our strong networks and Co-A and collaborator built-in considerable pathways to influence (see Outputs p17).

AIMS AND OBJECTIVES

Our solution-focused aim: We aim to contribute and inform evidence-based formal and informal strategies, guidelines, recommendations and interventions for health and social care policy and practice during and after the COVID-19 pandemic and system recovery (including any future waves), to mitigate inequities and improve the experiences and health and wellbeing outcomes of minority ethnic groups at the intersection with chronic conditions/disabilities. To do so, we will develop a rich understanding of their mental and physical health, coping, access to resources, and informal and formal social and health care support experiences, and relevant assets and strengths, longitudinally over 18 months using mixed methods, examining variations through an intersectionality lens. Analyses, outputs, dissemination and implementation plans for these will be co-developed with key stakeholders (see 'Key Audiences').

Objectives: Using an intersectionality lens our objectives are to:

O1: Explore and compare, by location and time, survey and qualitative data on changing patterns of need. Including intersections of chronic condition/disability and ethnicity/citizenship state with UK pandemic contexts.

O2: Relate pandemic coping strategies/solutions to O1 findings, including what worked well or less well, and touchpoints (where experiences might best be improved), to inform health and social care policy and practice

O3: Use Social Network Analysis to explore formal and informal network issues/affordances in health and social care solutions

O4: Gain insights from comparisons and relationships across our mixed methods data, rapid framework-based synthesis of the published and grey literature, and secondary analyses of UCL's Centre for Longitudinal Studies (CLS) and ActEarly COVID-19 specific surveys.

O5: Contextualise and explore transferability of qualitative findings using the survey, and survey findings using CLS/ActEarly UK census data.

O6: Co-create with stakeholders (including PwCD/minority ethnicities) interim/final outputs that include identified strategies, interventions and touchpoints, and plans for rapid pathways to impact.

Our intersectional lens enables:

- potential stratification by risk levels to inform preventative/care action
- suggested strategies and interventions appropriate for different intersecting structural, cultural and religious needs, levels of deprivation, ages, gender and other factors shown through our research to be relevant.

Access to resources, formal and informal care, social networks and links to health/social care outcomes are foregrounded as these are protective for pandemic mental health (18,50) and the wider non-pandemic literature suggests psychological and social support factors enhance general wellbeing (21).

RESEARCH PLAN / METHODS

Our 4 work packages (WP) involve a new UK survey in 3 waves, parallel qualitative insights, secondary analyses of other surveys, rapid review, and outputs for immediate use developed with participatory methods. Our approach, remote, takes account of pandemic constraints. We can easily revert to face-to-face work if appropriate. Our study is ambitious and we have therefore put in place a team that is particularly well placed to achieve this work (See Expertise, p20).

Theoretical framework: Embodied experiences of chronic conditions and disabilities, being shaped in and through social interactions (including with health and social care and support) (51), are necessarily intersectional with areas of potential discrimination and oppression (Box 1) across the levels of **Bronfenbrenner's socioecological model (SEM**) (52), hence the need for a range of comparisons and involvement of multiple stakeholders in our study. This model will frame our translation of findings into clear recommendations for varied audiences and fits with the new NHS tiered Integrated Care plan (48). The model levels range from smaller, proximal settings of local influence to larger, distal settings

with indirect influence. The bidirectional and dynamic nature of intersectional interactions across the levels means mutual constitutions are in constant flux, emphasising the need for a longitudinal study. Our work is also underpinned by the **Consolidated Framework for Implementation Research** (CFIR) (49) because, as an amalgamation of a range of existing implementation theories, it cuts across the levels of the SEM, with a comprehensive range of constructs. The CFIR is easy to operationalise, flexible (the user selects only themes from a pool of 39 that are relevant), and provides actionable findings across multilevel implementation contexts. These theories will inform interview topic guides, and survey questions for topics 3, 4, 7 and 8 in the next section. In our analysis they will inform health and social care mapping and implementation considerations across multiple levels for integrated care.

Topics across all WP: These are as follows (in the survey based on questionnaires, mostly validated in ethnic minorities including recent migrants) and include a range of variables that the research evidence suggests are key influencers of pandemic health and wellbeing. We believe by focusing on these, we should have a big impact through small changes. We note that other variables such as access to education will also be important but are not of direct relevance in terms of our aims:

1. **Intersectionalities,** the importance of which we explained on page 1 (we use a recently developed framework [53])
2. **Behavioural responses** to COVID risk-reduction measures including vaccination by individuals and their formal/informal support and care networks (e.g. friends, family, community, health/social care) – to understand the context of peoples' lives, what responses are feasible or acceptable to them, and effects on their networks. This will help us build up a picture of potential assets and strengths and affordances (as well as issues).
3. **Access** to resources, formal/informal support and care, including **digital transformation**, service innovations – as explained on p 3, good support and resource access is protective for pandemic mental health (18), and it also mitigates other health issues.
4. **Social network** (formal/informal support and care networks) descriptions - the importance of which we describe on p2 (contextualising topic 2 above for network behaviours)
5. **Coping and attitudes, physical and mental health** consequences of the pandemic, why they arose and how issues can be mitigated.
6. Mental and physical wellbeing/**quality of life** as core outcomes
7. **Local/regional differences** in responses linked to policies/interventions and associated impacts
8. **Future** policy implementation that is accessible to PwCD and minority ethnic groups.

In all cases we will consider what has worked well and less well for health and wellbeing outcomes, ensuring policy-relevant comparison and synthesis across WP.

WP1: SECONDARY ANALYSES (1-3 AND 14-17 MONTHS) (for O4) (cost £150,437 FEC)

This stage informs WP2-4. We will map and synthesise existing quantitative and qualitative evidence on the pandemic and PwCD or ethnic minorities that supports our aims.

Textual data on our 8 topics: Given the novel and unstable nature of the pandemic, its sequelae and system recovery, this includes pre-print resources such as medRxiv and less formal sources such as blogs, Google, Reddit and Twitter searches. Also more usual database searches using UCL's EPPI-Reviewer 4 software, the UCL EPPI centre's COVID-19 Living Map of Evidence, the WHO Global Research Database on COVID-19, the COVID-19 Rapid Evidence Reviews Group (CORRE), and LitCOVID. These databases bring together evidence on COVID-19 from a worldwide dataset; we will add other sources that we locate. **Social media searches** will be run on identified hashtags and frequent users who discuss our 8 topics, tailored by site in consultation with our PPI group. Relevant threads will be analysed using open-source bespoke software e.g. GATE (54), including twitter network maps, and thematic content analysis. Extracts used in dissemination will be paraphrased to avoid identification. For **articles, reports, and longer texts**, we will search using controlled subject headings and keywords such as 1) COVID-19 OR COVID OR SARS-CoV-2 or pandemic; 2) long-term OR post-acute OR long-tail OR persistent OR chronic COVID OR long Covid OR post$discharge OR prolonged symptoms OR long haul; 3) ethnic* OR minority ethnic* or variants thereof including specific groups; 4) chronic OR long$term OR underlying OR [names for chronic conditions or chronic health states] OR disab*; 5) specific terms related to our 8 topics running through the study (e.g. for topic 6 synonyms of 'wellbeing' OR 'quality of life'). Two reviewers will screen titles, abstracts and full texts against inclusion criteria, with disagreements resolved by 3rd third researcher. We will use quality assessments designed for each study type, with additional reference to the provenance and publication status of sources. Data will be extracted by one reviewer and checked by a 2nd. We will use descriptive statistics and meta-

analysis to summarise data where appropriate, and otherwise narrative synthesis. We will perform subgroup analyses (see WP3 for potential research questions for these). These data will give us a grounding in current research and other evidence, in a fast-moving pandemic-responsive field, to ensure we tackle our aims in the most useful way, and will help to provide themes to incorporate in the WP2 and WP3 data.

CLS and ActEarly cohort studies: We will undertake secondary analysis of a subset (those with chronic conditions/disabilities) of the UKRI-funded **ActEarly city collaboratory** consortium (55) COVID surveys. These complement our surveys and are similarly supported by qualitative data. ActEarly collects data of interest in two ethnically diverse areas, Bradford and East London, on physical health (including general health, health anxieties, health behaviours and mental health), relevant demographic factors, services access, and family relationships and social support. Co-applicant Dickerson, Director of the Better Start Bradford Innovation Hub and Bradford Inequalities Research Unit is Programme Manager of the Born in Bradford (BiB) partners of the ActEarly consortium. The London partners include members of the PI's department and our Bromley by Bow collaborator. We have chosen Bradford and London as two of our own sites for the same reason as this consortium and hence our work will be complementary. Both areas have strong reputations in applied health research with a focus on health inequalities in deprived and ethnic minority populations and deep engagement with the community and local policymakers. Inequalities are extreme in both areas, making it likely that strategies successful in mitigating the adverse impacts of COVID-19 there are likely to be transferable to other places with less extreme conditions. In both areas, research groups represented by Dickerson and Bromley by Bow Centre, working with policymakers have built strong networks across health care providers, connecting multiple systems. The initial focus of the ActEarly Covid-19 surveys has been on children, parents and pregnant women (a sample pool of just under 14,500 in Bradford (56) and a focused sample of 2000 in East London), which avoids participant research burden from our study. **CLS**, part of the PI's UCL department, has run COVID-19 surveys within the nationally representative cohort studies it curates, with respondents aged 19-74: Millennium Cohort Study (born 2000-02), Next Steps (born 1989-90),1970 British Cohort Study, 1958 National Child Development Study, and also the MRC's National Survey of Health and Development (1946 British birth cohort). Wave 1, with over 18,000 respondents, took place in May 2020, and Wave 2, with almost 26,000 respondents, in September 2020. These included items on physical health (including COVID-19), health behaviours, demographics, mental health, social connectedness and health care, hence relevant to our aims though the fit is not as close as for ActEarly. We will also draw on UCL's COVID-19 Longitudinal Research and Evidence Tracker which trawls for COVID-19 longitudinal research and evidence, e.g. briefing notes, reports, articles. Evidence from the tracker may provide further useful information and context.

Data synthesis: This follows a previous design for COVID mental health care data (17) to produce a narrative synthesis of text data, as a mapping review, and thematically linked summary quantitative analyses from the cohort studies. A matrix-based approach will be used for initial mapping, with sources as rows, themes as columns. Our initial 1-3-month work will be updated in months 14-17.

WP1 Evidence outputs:

1. Identification and quantitative/narrative summary of themes considering the pandemic's impact on PwCD and/or minority ethnicities, that focus on our 8 topics (p6), from each of:
    a. the peer reviewed and grey literature
    b. social media
    c. existing cohort studies
2. Identification and quantitative/narrative summary of themes considering access to resources, formal and informal care, social network and mental and physical health/wellbeing consequences of the pandemic on PwCD and/or ethnic minorities from the same 3 sources
3. An understanding of the pandemic lived experience for PwCD and/or minority ethnicities
4. Research findings to inform and be synthesised with WP2-4.

WP2: NEW SURVEY (2,8,15 MONTHS) (O1-5) (cost £195,303 FEC)

Our new survey considers our 8 core topics (p6), informed by WP1 secondary analyses, in 3 waves, for 1st and 2nd generation community-dwelling minority ethnic groups and white British comparators, all with/without chronic conditions/disabilities, across the UK's 4 nations. It enables us to determine relationships between variables and trajectories relevant to our aims. We scoped other COVID-19 surveys and as explained in our Review of the literature on p3 and for WP1 cohort studies, all existing surveys have considerable differences to ours. An existing similar survey undertaken by colleagues of the PI in Tower Hamlets and the BiB survey, both for ActEarly, have provided a testing ground, and we

will use the data in WP1. These surveys consider pregnant women and families with young children, with a focus on childhood; our survey is thus unique and extends and enlarges their scope.
Analysis will exploit all 3 waves of the data, with these **research questions (RQ):**

1. *How do outcomes (resource access, formal/informal care, quality of life, control of life, physical and mental health, social networks) and outcome trajectories differ by sample subgroups?*
2. *What are the outcomes (resource access, formal/informal care, quality of life, control of life, physical and mental health, social networks) and their trajectories in terms of intersectionalities?*
3. *To what extent can pandemic prevalence and adherence to social distancing at the area level explain differences in outcomes and outcome trajectories across subgroups of the sample and in terms of intersectionalities?*
4. *How do the outcomes inter-relate within and across survey waves, and how does this differ across subgroups of the sample and in terms of intersectionalities?*

A full-time post-doctoral researcher will undertake the main survey work. We will develop the final survey format with our PPI group and pilot it with N=30 before fielding it at scale. The survey will be online but with telephone interviews (CATI) where needed. To minimise respondent burden, each wave will be completable within 15 minutes, which ActEarly and CLS work shows is acceptable for COVID-19 surveys. Surveys will begin with informed consent/screening questions. Then the study topics will be explored, based on validated questionnaires, mostly validated in migrant and ethnic minority groups as well as the dominant population **(Box 2).** Surveys in different waves will differ. First, theoretically stable concepts (e.g. tolerance to uncertainty, demographic characteristics) will be measured only in one wave. Second, key topics identified in prior wave(s), qualitative work (WP3) and our co-production and engagement work running through the study may be added. But key outcome and exposure variables that we expect to change during the pandemic as specified in our WP2 RQ will be measured in all three waves to study trajectories.

Box 2. Our 8 topics and corresponding survey instruments/items

Topic	Questions
Intersectionalities	Demographics including year of birth, gender, ethnicity, relationship status, area code of postcode, urban/rural dwelling, accommodation type, household income, education, employment status, religiosity (57)
Behavioural responses ‘	Control of life’ (including COVID-19-related)
Access to resources, support, care, vaccines	QOCS–ID (58), Vulnerability Assessment Framework (59) for care needs, UK government SAGE group recommended questions (https://bit.ly/2OZN9Bf)
Social networks	Developed from the close persons questionnaire (60)
Mental and physical wellbeing/quality of life	WHOQOL-BREF-ID (58)
Coping	Including tolerance to uncertainty, positive appraisal style, attitudes to being ill/disabled (WHO ADS (58)), health and mental health consequences (Global Mental Health Assessment Tool (61)) of the pandemic, why they arose and how issues can be mitigated
Local and regional differences	Apart from within-survey analysis, we will match respondents’ area code of postcode with area-level (i) registered COVID-19 cases, hospitalisations and deaths (ONS https://bit.ly/2NOydC8) and (ii) social distancing adherence (Google Community Mobility https://bit.ly/2AqRwyk).
Vaccines, Future policies	Freetext comment boxes

Quantitative analysis: A **descriptive statistical summary** will be updated with each wave using RedCap analytical tools for rapid dissemination. For **RQ1**, we will exploit the longitudinal nature of the data using **Latent Growth Modelling** (LGM) to estimate (i) levels of network capital, quality of life, mental and physical health and other key variables, and (ii) change in these over time, and differences in levels and changes between those with chronic conditions versus without, white British versus ethnic minority, and citizenship versus without. For **RQ2**, we will carry out “multiple group” analysis with separate levels and trajectories of the key variables estimated per minority, condition/ disability and citizenship intersectionality. In other words, we will vary different combinations to consider the effect of intersectionalities on outcomes. For **RQ3**, we will include area-level matched data on pandemic

prevalence and adherence to social distancing in the latent growth models as covariates, to explore the extent they can explain differences in outcomes between each subgroup, and differences compared with inter-sectional combinations. Depending on geographical coverage and the numbers recruited, the LGM estimation could be carried out within-area, to examine the causal impact of the change in pandemic prevalence and social distancing across the waves on each key outcome, under the assumption that change in pandemic prevalence and social distancing is exogenous. We will examine the plausibility of this assumption and detail possible sources of endogeneity. **This will be important for policy, with its unique focus on ethnic minorities and PwCD.** For example, our data could help clarify why specific groups may not find it feasible to adhere to recommended behavioural responses. Finally, for **RQ4** we will estimate a developmental cascade model, including all 3 data waves and key variables, to explore how the key variables are associated with one another, both within survey wave and over time. We will fit the LGM models using Structural Equation Modelling (SEM); this offers useful tools for dealing with missing data due to non-response and attrition; the Full Information Maximum Likelihood estimation will be used for possible systematic missingness. The **Social Network Module** will provide an ego(participant)-centred network, including ego's ties, their frequency, resources of the alters (others), relationship types. Further characteristics e.g. the ego network's size, density, composition, and average strength of ties will be calculated and a latent "network capital" variable created through measurement analysis within the SEM as a novel contribution. A Confirmatory Factor Analysis will refine other survey measures.

WP2 Evidence outputs:

1. Identification and description of how pandemic-relevant outcomes (resource access, formal/informal care, quality of life, control of life, physical and mental health, social networks) and outcome trajectories differ across subgroups of people of different ethnicities including white British and with or without chronic conditions or disabilities.
2. Identification and description of outcomes and outcome trajectories in terms of intersectionalities
3. Identification and description of any emerging associations over time and within cross-sectional subgroups, including in terms of a) COVID-19 case prevalence and adherence to social distancing at the area level, b) long covid and vaccine uptake/hesitancy, c) intersectionalities
4. Risk stratification of groups to inform preventative/care action
5. Research findings to inform the early WP3 work and for synthesis with WP1, WP3, WP4 findings.

WP3: QUALITATIVE WORK (3, 9, 15 MONTHS) (O1-5) (cost £306,849.55 FEC)

In-depth insights will be added from 3 qualitative waves exploring the experiences of 1st and 2nd generation community-dwelling Arabs, Poles, Indians/Pakistanis and sub-Saharan Africans, and, for comparison, white British, all with/without chronic conditions/disabilities. **WP1** and **WP2** findings will feed into this, contributing possible questions for our topic guide, which will be designed around the same topics as shown in Box 2 and p6. All qualitative data sessions will be recorded with consent and transcribed. Interviews will last up to 1 hour, workshops 2 hours. Participants will receive thank you vouchers (online, posted or by hand depending on extant pandemic guidance/personal request).

WAVE 1: SEMI-STRUCTURED INTERVIEWS

Remote by default, will use the method of respondents' choosing (most likely phone (62)). At each site, our PPI leads will train a lay community member to undertake interviews locally –still planned remotely - supported by 2 central qualitative researchers who will also undertake remote work.

Many participants are likely to depend on informal **support networks** for: education; health, social care, legal matters; wellbeing; social/cultural activities (21). These, formal support and third sector networks have been disrupted during the pandemic. We explore the issues through:

- A brief questionnaire about social networks preceding the first interview – orally if needed
- Results translated into Network Canvas software to develop ego(participant)-centric network maps that can be explored in depth in interviews
- Participant sketch-maps of their local area and the places significant to them.

For inclusivity, important when considering people with disabilities and ethnic minority/migrant groups, participants will be asked to take smartphone photographs of significant places prior to interview. This ethnographic approach facilitates a safe social space to communicate difficult issues and has been used to explore migrant resettlement (63). We will give all participants clear instructions, with a focus on ethical issues (e.g. to avoid identifiable photos of other people). Participants with no/unsuitable phones, no internet, limited data plans will be given **disposable cameras** with SD cards (local researchers will arrange pandemic-safe digital data collection). Participants will be asked to take photos

significant to their healthcare interactions and to their social interactions. Photos will be discussed in interview, to probe for insights, and will also be thematically analysed as data. They might for example show physical barriers to accessing a building, or a photo of a restricted gathering for a wedding or a funeral, which can be used to stimulate discussion about our key topics. Interview data will undergo Keyword in Context (word frequency-based) analysis and constructs used to develop a coding frame for Framework analysis of the workshop, interview, photo and key informant data, for general dissemination and policy-relevant themes that can be mapped to the survey for added insight. Added to this deductive approach we will allow for inductive themes. Data collection and analysis will be concurrent for quick outputs and to test emerging and discordant themes and will continue until sufficient 'inductive thematic saturation' is reached. We will undertake discourse and narrative analyses on a data subset produced from participant pairs matched on features identified as important from analysis.This subset approach, used by Rivas successfully before (64), makes optimal use of the large dataset. The discourse analysis will examine the linguistic and discursive resources participants use to link social practices (e.g. ableism, racism) to their situation and with the narrative analysis of participant 'stories' will further contextualise findings on wellbeing and coping, social identity and networks, attitude to and relationship with services - important for services to build trust. Black peoples' mistrust of the COVID vaccine is relevant here for example. These analyses will reveal potential mechanisms for behavioural responses to help us develop our evidence to decision interrogation (WP4). We will ensure credibility/internal and external validity (e.g. through exemplar data extracts, data collection triangulation, and team and participant workshop data discussions), transparency (with a clear audit trial) and reproducibility (with thick descriptions of context and analysis) and anonymised data will be archived for secondary analyses.

WAVES 2/3: RESEARCH WORKSHOPS

Likely remote, due to the pandemic, e.g. using 'Teams'. To avoid excluding people, we will also offer repeat interviews. The precise make-up/number of workshop groups per wave will be determined from Wave 1 data. We will work with the UCL Centre for Collaboration and our PPI group to ensure workshops are accessible and inclusive, e.g. incorporating Padlet, Miro and other visual tools or infographics where appropriate. Wave 2 workshops will discuss scenarios, or **structured vignettes**, shown as short videos recorded by community members reading scripts; content will be developed from Wave 1 data into a pandemic-relevant story, illustrated e.g. with Wave 1 photos with permission, to consider assets and strengths, issues and potential solutions. Non-identifiable verbatim phrases will enhance authenticity, with accessibility transcripts provided in advance. This approach is effective in inclusive research and suited to both remote and face to face work, so we can be flexible. Discussion will serve to check validity of previous findings and consider changes from these. Wave 3 workshops will be similar, with updated vignettes. Team members have used a similar approach in previous (36) or current (Rivas: CoGS, Crescendo, see cv) projects. We will also use **participatory scenario planning** (66), a policy tool whereby participants are encouraged to imagine and explore alternative futures, their impacts and relevant action plans (topic 8, p6).

CO-CREATE WORKSHOPS through the study (O6, cost incorporated in other costs)

are distinct from WP2 research workshops though based on similar principles. Key differences are that they will: a) include a range of other stakeholders as well as ethnic minorities with chronic conditions/disability; b) contribute to analyses <u>in addition to</u> translating findings into practice. All 3 workshop sets (wave 2, wave 3, co-create) will aim for outputs relevant and implementable for the 'real world' that maintain participant voices, with tangible benefits for all. Materials will be shared in advance, to suit accessibility needs, and discussions led by our PPI lead and PI. Sessions will last 4 hours (2 if remote), with practical activities that empower all those attending to contribute as equals to 'negotiated' analyses/outputs. Group discussions will be summarised for reference. The research team will work on outputs/analyses to present at following workshops so outputs/analyses are developed iteratively through the co-create workshops.

WP3 Evidence outputs:

1. Characterisation of social network issues/affordances that can inform health and social care changes and improve pandemic health and wellbeing for ethnic minorities with PwCD
2. Framework and word frequency analyses for practice- and policy-relevant themes that can be mapped to the survey findings for the same topics
3. In-depth, theoretically-informed understanding of lived experiences of ethnic minorities with PwCD - important for services to build trust

4. Description of imagined alternative future scenarios to help with future policy and practice planning for ethnic minorities with PwCD
5. Research findings to be synthesised with WP1, WP2, WP4.

WP4: PARTICIPATORY IMPACT/OUTPUT DEVELOPMENT (O6) (cost £178,351 FEC)

1. 15-25 **interviews** (16 months) (up to 5 per site) are planned **with key informants** as determined from other WP: e.g. welfare, social and health care staff, settlement and ethno-specific services, the third sector and community leaders. To support implementation into policy and practice, topics include perceptions of local service needs, area-level characteristics, barriers and facilitators to community member service access, current community-led responses, impacts and effects of the pandemic, and future planning. Topic guides will also consider CFIR themes (49).
2. **Data synthesis** will provide an executive overview for easy digestion by policymakers and practitioners and help show where health/social care policy and practice changes are likely to be most effective. Synthesis will be results-based, with thematic tabulation derived from WP1-3 data analyses, with table columns for themes, rows giving quantitative and qualitative data. Some data will need to be transformed (quantified or qualitised) for tabulation e.g. network graphs. We will interrogate the tabulated data using anchor questions based on the PerSPectif framework (67) (informed e.g. by data convergence/divergence patterns). This will form the basis of guidance and recommendations outputs and will inform interventions. An example question might be: *"From the **per**spective of a first generation single woman aged 18-30 of African descent, with complex dietary needs, in a rural Yorkshire **s**etting, how does the **p**henomenon of community-based food banks, within an **e**nvironment of poor transport infrastructure and geographically remote facilities (a food desert) **c**ompare with online shopping during a **t**ime of national lockdown in relation to **f**indings on their perspectives and key study outcomes*". Specific questions will be based on data findings, e.g. intersectional variables and outcomes. Disaggregation will be built in where possible and useful, though in a complex systems approach this may sometimes be misleading due to system interdependencies. Systems diagrams, logic models and other approaches will be used where appropriate. This evidence to decision approach supports our aim to report explanatory findings with practical application.
3. We will try out adaptations of two existing training programmes, **Tough Cookie** for community members and **Pain Relief Management** for practitioners, based on our findings, at our 5 sites for proof-of-concept. We do not plan to test any other interventions (please see the 'Team' section for details of these programmes)
4. Our final mixed stakeholder a) advisory group and b) **participatory (co-create) workshop**, CIFR-themed, with our PPI group and key informants will develop with us the final implementation, dissemination and impact plan.

SAMPLING

WP2 Survey sampling: We will **recruit** via social media and national networks (e.g. academic, NHS, third sector) including our existing networks and mailing lists, and large databases of adults interested in health research across the UK. These include UCL BioResource and HealthWise Wales, the NIHR Be Part of Research, the NIHR Research Design Service Public Involvement groups and networks registries, the UKRI Mental Health Research Networks, The Covid-19 Research Involvement Group, The Covid-19 Support Group. We will also recruit via the various social media patient and migrant groups with which we are connected and the specialist third sector organisations in our Team section list. This enables good reach and access across the spread of chronic conditions/disabilities, ethnic minorities and citizenship states and connection with people already wishing to take part in research. We also have access to the ActEarly city collaboratories in Bradford and East London if needed, and the Gateshead long Covid clinic being set up by Saravanan, though these will be a focus rather of WP3 recruitment. We recognise our recruitment, being non-randomised, will be biased, for example to those already interested in research participation or who are active users of third sector sites and have online access. We will compare respondent demographics to whole population estimates where possible (though formal data are limited) to explore **representativeness**.

Survey numbers and power: The primary aim of the quantitative data is to describe the trajectories of key variables and outcomes (e.g. quality of life, services access, networks), and the links between them, in ethnic minority and PwCD communities, in comparison to the White British. The longitudinal survey will not be used to test a particular treatment or focus on a single effect. Considering power in Structural Equation Modelling (SEM), the required sample size depends on several factors (68). First, the required

sample size increases with the number of latent variables, but at a decreasing rate (i.e. the required sample size difference between a model with one versus two latent variables is larger than that between a model with three versus two latent variables). Second is the size of the loadings on latent variables. Required sample size decreases strongly as the loadings increase. Finally, power increases as the number of items used to measure each latent variable increases. In our basic SEM, we have six core latent variables per wave: quality of life, control of life, access to care, coping mechanisms, mental health, and social networks. Each will be measured by several items (the average number being more than 8). In a worst-case scenario with average loadings of around 0.5 and an item missingness of 20% (as suggested from ActEarly work by co-applicant Dickerson, a sample size of 800 per subgroup per wave will yield useful analyses. We have four main subgroups (i.e. minority ethnic, minority ethnic+ PwCC, White British, White British + PwCC). Hence, a sufficient target sample size is 800*4=3,200 though we aim for 5,000 for stronger data (68).

Survey target number risks: We are confident of achieving our target numbers because of our comprehensive recruitment strategy (as described at the start of this section on Sampling), extensive networks, prior experience with the marginalised, and the experiences of other pandemic surveys including those of co-applicants. We also believe the emergence of long Covid critical mass and the particular desire of people with marginalised chronic conditions such as long Covid and complex (multisystemic) comorbidity (such as Ehlers Danlos Syndromes, Chronic Fatigue, Fibromyalgia) to contribute to research to get their voices heard, means there will be a strong appetite for our survey. Should we over- or under-sample, we will use all the data; under-sampling may preclude within-area analyses. There are two particular risks that require mitigation:

1. Should we fail to achieve even 3,200 at wave 1, we will reduce the study design to a two-wave survey, leaving recruitment open for longer at each wave. In the worst case, we will only undertake one wave. However, we consider these mitigation scenarios highly unlikely from initial scoping and from past experience of team members for other surveys.
2. Failure to recruit enough minority ethnic participants - the above shows the minimum number needed is 1,600 BAME respondents. Several team members have considerable experience in recruiting ethnic minorities with chronic health problems specifically (Goff; Bromley-by-Bow Centre; Dickerson, Programme Manager of Born in Bradford/ActEarly) and we believe minimum numbers can be reached with a four nations survey. In Dickerson's successful localised 2020 BiB survey, only 18% of 2,144 respondents were White British. We have ensured strong connections also with ethnic minority organisations that deal specifically with chronic conditions, such as collaborators MedAct and via collaborator Abou-Saleh. So we believe we will be successful in our plans. However should this not be so, we will be able to undertake useful analyses of white British data and can then explore ethnic intersectionalities within WP3 and by collapsing ethnic minority +/- PwCC samples in the survey. See also our methods of dealing with **attrition** in the next paragraphs.

Survey attrition and missing data: We will require completion of almost every question on every page for participants to proceed, so we can undertake the association analyses required. This means there should generally be no *missing* items in any measures, though this requirement may lead to *completion attrition*, with respondents giving up and logging off. We will try to mitigate that with the questionnaire design which will be developed and piloted with our PPI group and N=30 others. There is the risk of *attrition between waves*. Participants will be asked to provide an email address on enrolling, if online. The RedCap online secure system will then automatically re-contact them for wave 2/3 follow-up questionnaires (with reminders) to explore trajectories over time. This automatic process makes for efficient and secure second and third wave recruitment to reduce the risk of missing respondents. Data will be anonymised prior to analysis and researchers will not directly handle email addresses; however they can control reminders. Careful design of the covering letter/page on between-wave reminders can improve return rates from those with lower levels of education or who speak languages other than English at home (69), so we have ensured these are designed with full PPI input. Lotteries appear effective in some online surveys (69) and we are including a £50 Amazon voucher as an incentive given at random. To handle missing data and address panel attrition and item non-response, we will use modern methods, including Full Information Maximum Likelihood, Multiple Imputation with Chained Equations that produce unbiased estimates under assumptions of missing at random (i.e. missingness depend on observable data only) and multivariate normality; and pattern mixture models that address missing not at random (i.e. missingness may also depend on unobserved data) assuming correct model specification (70). Those techniques, under certain assumptions, ameliorate loss of statistical power due to missing data and possible biases due to systematic missingness.

WP3 interview sampling: We aim for 210 interviews with purposive quota sampling (**Table 1**) for maximal diversity and sufficient numbers for rich data for each group, and recruitment through the same channels as in WP2. Posters, adverts and snowballing will target those who lack resources or technology to respond to online recruitment (62), for example via our clinical co-applicants and our collaborators. (Though many migrants or their local groups use digital technologies e.g. to contact 'home' (71), for pandemic faith meetings.) The 40-plus newly set-up long Covid specialist clinics will provide a further possibility of recruitment, for example via posters. Our sampling frame follows an intersectional studies approach that allows us to consider and compare assumed homogeneity across condition effects irrespective of ethnicity, and across ethnicity irrespective of condition, as a tool to tease out intersectional factors and heterogeneity. At analysis the focus may switch to other commonalities such as shared barriers or facilitators to health and social care resources.

Table 1: Interview sampling frame (cells contain numbers to be sampled)

Ethnic/national origin / ***Condition effect***	*Migrant: Middle East*	*Migrant: sub-Saharan Africa*	*Migrant: Poland*	*Migrant: India and Pakistan*	*2nd Gener-ation*	*White British*	*TOTAL*
Mental	5	5	5	5	5	5	**30**
Mobility	5	5	5	5	5	5	**30**
Stamina/breathing/ fatigue (incl. heart)	5	5	5	5	5	5	**30**
Hearing/Vision loss	5	5	5	5	5	5	**30**
Developmental/ intellectual	5	5	5	5	5	5	**30**
Dietary	5	5	5	5	5	5	**30**
No condition/ disability	5	5	5	5	5	5	**30**
TOTAL	**30**	**30**	**30**	**30**	**30**	**30**	**210**

Main interview inclusion criteria:

- White British comparators or Arab, Polish, Indian, Pakistani or sub-Saharan African 1st or 2nd generation refugees/migrant adults aged 18+ (undocumented, on temporary visas, indefinite leave to remain, British citizenship) (Table 1). While not homogenous, these groups were chosen to be diverse but focused enough to ensure rich data and on the basis that a) 74% of refugees resettled in the UK since 2010 were Arabs and Turks, 19% sub-Saharan Africans (72) (who are also the most likely to die from COVID-19 in the UK (16)), b) recent migrants by choice were mostly born in Poland or India (72) and c) the 2nd highest UK COVID-19 mortality rates by ethnicity are for people of Pakistani origin (16).
- Any condition/disability, including self-diagnosis, that chronically affects daily activities.

We include skilled migration, humanitarian or family streams, the 'irregular' or undocumented, on temporary visas or first-generation migrants with indefinite leave to remain, or first or second generations with British citizenship.

Exclusions: a) student migrants as likely to have structured educational institution support, and b) residents of detention centres/closed facilities linked to national migration policies (e.g. new asylum-seekers/refugees, displaced or trafficked persons), as complex cases with specific considerations.

Risks of non-recruitment and attrition: We aim for sufficient participants for rich data for all our main ethnicity/disability combinations shown in Table 1. Possible attrition (up to 20% based on BiB experience) between waves may require further recruitment if theme/pattern saturation is not reached. But if many combinations provide similar data, leading to saturation, we may stop recruitment early or modify our recruitment strategy for theoretical sampling. To reduce risks of non-recruitment and attrition, and to enable us to achieve our aims, we have considerable in-built capacity to do this work. Rivas previously managed a similar-sized interview study (73) over 12 months with three researchers interviewing 217 men with cancer remotely whilst working on other projects and parts of the study. At stage 1 she calculated resource requirements for the current study based on this experience but since then we have added further capacity in response to panel feedback.

We have two **full time qualitative researchers** to cover the interview and workshop periods of the study. Our PPI leads and qualitative postdoctoral researcher will train local **lay co-researchers at each site** as part of the core study design. Our named PPI representatives as co-applicants and collaborators have also been costed for possible interviewing co-researcher work. Additionally, **Bromley by Bow** will undertake direct recruitment and interviewing as **co-researchers**. Their involvement is significant as

they have direct experience in undertaking similar work with local migrant groups in the past, and access to suitable participants. This community group is confident of recruiting and interviewing the required sample and running the London workshops. It is run by lay people who belong to the communities of interest and is connected to an adjacent health centre. Likewise, Dickerson is Programme Manager for **Born in Bradford** and has direct access to suitable participants and co-researchers in this research-ready city collaboratory. PI Rivas and co-applicant Redclift will also do interviews if necessary; both have considerable expertise in this with migrant populations. All people undertaking interviews will be fully trained to ensure a consistent approach.

Risks for participant mental health/safeguarding: Abou-Saleh who undertakes migrant crisis assessments for the Helen Bamber Foundation will advise and we will provide clear signposting to sources of help. Immigration concerns need assured anonymity and sensitivity.

WP4 Key informant interviews: Recruited through our team, collaborator and Advisory Group networks and emails to local service providers and organisations.

SUMMARY OF PATIENTS/SERVICE USERS/PUBLIC AS RESEARCH PARTICIPANTS

Inclusion/exclusion criteria: We have taken care to involve a range of ethnicities and a full range of disabilities including long Covid and self-diagnoses (see p1 for full rationale). Our exclusions are based on relevance and safety considerations as advised when our study underwent ethics review. Dickerson, BiB Programme Manager, has taken care to ensure we will not sample the same groups as the BiB current COVID work to avoid research burden. Our PPI advisers suggested we only interview participants able to communicate in English so the focus would not be on language fluency, which is a specific issue the PI has previously studied (37). We will be inclusive of disabilities through responsive accessibility formats.

Recruitment/consent process: We do not use randomised sampling but recruit participants from adverts/links distributed through a range of platforms and networks (please see p11), as well as local lay co-researchers for our qualitative work (see p14). Potential participants will be informed that interviews will be in English, and it is their choice as to whether they feel able to take part. This is a well formulated and effective process in Born in Bradford. Where a participant is happy to interview in English, but feels more comfortable doing so in their home language, if a researcher fluent in that language is available this will be arranged.

Our consent process has ethics approval and will be in English as default. Translated study documents will be made available if required specifically to ensure fully informed consent. Our focus on specific groups makes this manageable. Braille and other formats e.g. for neurodiversity will be used if needed. **Participant information materials** have been ethics-approved and for interviews and workshops are in print/digital form. For surveys they precede survey responses and the consent form, online. They have been piloted within relevant communities. We include approaches to increase survey respondent retention (p13). Bromley by Bow collaborators are a community group involved in similar research already; they were initially contacted to support recruitment but offered to be co-researchers. They are fully costed in, bring considerable experience and are lay members of relevant communities.

Research methods and frequency of data capture: We have three waves of a 15-minute survey for each WP1 participant spread over the study. For each WP3 participant we have a 1-hour interview including network/map/photo elicitation methods, and two 2-hour remote research workshops (designed to minimise research burden) spread over the study. We have 5 stakeholder 2-hour co-create workshops with different participants and 15 x 1-hour key informant interviews.

Study participant support: We include lay co-researchers locally, partly because they will be sensitive to local situations and contexts, particularly relevant when the country is subdivided according to COVID risk, as well as cultural needs. We will ensure participants know they can drop out at any time without adverse consequences. Any participant mental ill health and distress will need advice from collaborator Abou-Saleh who undertakes migrant crisis assessments for the Helen Bamber Foundation and we provide signposting to sources of help. The ActEarly networks include service providers to whom participants could be referred if needed. As this study is planned to be fully remote, which supports access by people with chronic conditions/disabilities, our main concerns will be to match participant fatigue/wellness levels and to ensure frequent breaks. The PI has undertaken other pandemic projects and these have shown such measures ensure participants are not excluded. Consent and information documents may need to be prepared in specific formats such as special-coloured backgrounds and we will use online approaches such as Padlet or Miro only after ensuring they are accessible to participants; indeed these are likely to increase accessibility.

Sharing study progress and findings with study participants: See p17.
Payments, rewards and recognition for study participants: Remuneration follows INVOLVE recommendations. Participants may be acknowledged by name on our websites but we will discuss the issues with them to make sure they are fully aware of these first. Our PPI team members will be invited to be co-authors on outputs and supported in their own outputs, e.g. for the supportive journal Research for All which is free to contribute to and read and is run from the PI's department.

WP3 SETTING

We use 5 sites in England for maximal sampling diversity in migrant population density, proportion of EU to non-EU migrants, and reasons for migration (**Table 2**) to ensure project findings are transferable across the UK. We will use WP2 4 nations survey findings to contextualise and evaluate transferability of WP3 findings. This is important as we sample in England only for qualitative work due to differences in the devolved nations in responses to the pandemic and in health and social care systems. While this means some of our findings may be more relevant to NHS England, we expect principles to be similar across the four nations and will consider this in our reporting and outputs. We will ensure that within our sites we recruit from a mix of local communities well served by immigrant-specific services, and less service-rich communities.

Table 2: Relevant features of chosen sites (72)

Features **Site**	% of residents born abroad	non-EU % of all residents born abroad	Majority reason for coming
London	38%	68%	Work, asylum seekers, refugees
SE England, Canterbury	13.5%	58%	Work or to join family
Gatehead-Newcastle	13%	84%	Work or to join family
W Midlands, Birmingham	18%	75%	Work, also many to join family
Yorkshire, Leeds	10%	56%	Work and family

OUTPUTS, DISSEMINATION, AND ANTICIPATED IMPACT

Sharing study progress/findings with participants: will involve e.g. a) a website, b) regular newsletters, c) lay summaries in requested formats, d) infographics, e) webinars, f) a science café. All will be co-developed with our representative PPI group.
Wave outputs: Each wave of each WP will produce interim summaries of findings for different audiences (see next page) but also main outputs as below, for dissemination. The main platforms will be text and web-based. Recommendations and guidance will use an evidence to decision format (please see p11). More creative dissemination and outputs may use art, 3-D work, performance art or other relevant approaches if our co-design participants recommend it; similar award-winning work by co-A Thomson, a trained designer, is used internationally. Some may be developed as seed projects from the original funding.
WP1 main outputs are 2 review articles to inform health/social care policy and practice.
WP2 outputs (for each of the 3 waves including within and between wave and group comparisons):

1. ***A large database resource*** that focuses on PwCD and ethnic minorities and which we will share with other researchers or analysts e.g. via CLS
2. A unique development of a well characterised ***social networks module*** for our PwCD/ethnic minorities analyses
3. ***A summary of key mediators and moderators*** to target with service change outputs, focusing on assets, strengths, affordances
4. Group ***risk stratification*** using an evidence to decision format to inform preventative/care action.

WP3 outputs within each wave including within and between wave and group comparisons will be:

1. ***Co-created guidelines, recommendations, policy briefs, strategies and interventions*** that take account of what worked well/less well, assets, strengths and affordances, and touchpoints (where experiences might best be improved), to inform health and social care policy and practice, with ***evidence-based foci based on patterns in WP2 analyses***. For example, if medicines access is seen in WP2 to more strongly moderate physical health outcomes than other factors, and WP3 data suggest adaptations to practice that improve this, that might become a priority focus. A recommendation might

be to improve medicines access through automated delivery systems, guidance might be to describe a model of good practice in medicines automated delivery systems, and an intervention might be the resources needed to set up such a system. These broader recommendations will include sub-recommendation consideration of mechanisms and groups as per our ***evidence to decision analysis described in WP4***. Our intersectional lens enables suggested strategies and interventions appropriate for intersecting structural, cultural and religious needs, levels of deprivation, ages, gender and other factors shown through our research to be relevant.

2. Imagined alternative futures for policy and practice planning for ethnic minorities with PwCD.

WP4 outputs:

1. ***A report on co-produced understanding of current and future implementation*** needs into policy and practice for relevant outputs developed in WP1-3 drawing on WP4 syntheses and narrative summaries.
2. ***Adapted training programmes, Tough Cookie and Pain Relief Management*** *and* a report on ***proof-of-concept work*** with these.
3. ***Adaptations to WP3 outputs suggested by WP4 work.***
4. A co-developed final *implementation, dissemination and rapid impact plan*.
5. A report on *data representativeness and transferability of findings*.
6. Web-based and printable at-a-glance information, important for busy audiences, as summaries and infographics. These will provide acti***onable findings in the evidence to decision format***, helping users ***prioritise where change may be impactful*** and showing gaps in evidence or areas of uncertainty (where data diverge). All outputs will be publicly accessible. The focus will be on the intersection of various conditions and ethnicity. Outputs will be *disaggregated* by sampled groups and key mediators and moderators where possible and appropriate.
7. ***Creative co-produced outputs*** e.g. as above (final nature to be determined in the study).

Key audiences: Team members are involved directly with relevant communities for assured impact and reach to key audiences, a list of which will be finalised in the co-design workshops and from our emerging data. But this is the anticipated range:

- health/social care practitioners, NHS/social care managers/policy decision-makers, through our clinical leads, WHO/UN (via Abou-Saleh), EPPI-Centre, Born-in-Bradford/ActEarly sites (via Bromley by Bow and Dickerson)
- the third sector, including many of our collaborators
- academics in disabilities, migrant, medical/health disciplines, social networks, participatory work
- PwCD/ethnic minorities communities, as represented by PPI co-applicants and collaborators
- community organisations such as collaborator Bromley by Bow, religious institutions
- COVID-19 research, policy and practice groups and networks, including the All-Party Parliamentary Group (APPG) on Coronavirus, long Covid networks, NICE long Covid committee.

DISSEMINATION OF OUTPUTS: Cascaded dissemination at each data wave, tailored to our key audiences, will emphasise practical solutions and implementation, and will be co-developed with key stakeholders representing our audiences.

All audiences:

- A dedicated accessible ***project website*** hosting twitter feed, other social media links, news items, and regularly updated findings/outputs/summaries suitable for different audiences, with accessibility formats. The website will be linked to university webpages and collaborator sites to increase visibility (e.g. in search rankings)
- ***Webinar*** presentation of study-end findings and implications, to reach broad audiences, accessibility-aware and supplemented by transcripts.
- ***blogs***, new and existing (e.g. Barts MS Research Blog with 4,000 views/day)
- ***YouTube videos, mass media interviews and press releases***
- ***Creative disseminations*** to be determined during the study, e.g. performance art.

Policymakers (see examples of policymakers in Connecting to policy, health, social care below): ***policy briefs, evidence summaries, guidance, recommendations***. The PI's Department has strong policy links and special interest in evidence for policy and practice (for example the PI teaches a course called Evidence for Policy and Practice, we have staff seconded to What Works Centres, and run the EPPI centre [Evidence for Policy and Practice Information Centre]), so active engagement will be a strong

focus, supported by UCL's Public Policy Unit e.g. ***through academic-policy roundtables, Arm's Length Body dissemination/meetings e.g. via ActEarly, EPPI pathways, Research Insights briefings.***
Academics: 2+ Sociology/Migrant Health journal ***articles*** (e.g. Sociology; Journal of Migrant Health), conference ***talks, knowledge exchange event, guidance, recommendations***:

- **Connecting to policy, health, social care practitioners** e.g. social workers, community health teams, clinicians, medical organisations, WHO**:** 2+ ***articles, talks, knowledge exchange event, training, guidance, recommendations, educational case studies*** on the implications of findings disseminated by e.g. Royal Colleges, ***practitioner journals***.To ensure engagement and action from shared outputs and disseminations, we will determine the best approaches through stakeholder analyses in our co-create workshops, and draw on existing networks and possibilities, e.g.:
  - We will contribute directly to the ***NHSE Optimum Clinical Pathways consultation*** of the Association of British Neurologists through Dobson.
  - ***Clinician co-applicants*** and especially Dickerson, Bromley by Bow, Saravanan and Abou-Saleh provide local and large-scale direct routes to practice (see 'Team').
  - We will produce information useful for the **national vaccine strategy.**
  - We will develop **aspirational action plans with social care staff** embedded at sites e.g. within Bromley-by-Bow and Born in Bradford, and more broadly.
- The EPPI-Centre will adopt the review in embedded ***Dept of Health and What Works Centres*** work, which provides evidence summaries for policymakers and practitioners.
- Adding findings to UCL's/QMUL's COVID-19 research databases and the EPPI COVID-19 Map of Evidence and other ***living maps*** will help ensure reach, impact and discoverability.
- UCL's Public Policy Centre will support ***policy impact***. The study will provide regular research reports and updates to the Cabinet Office, other government departments, Public Health England, the APPG on Coronavirus, SAGE/shadow-SAGE (we are networked with members), Arms-Length-Body meetings e.g. through ActEarly, EPPI networks and the World Health Organisation (e.g. through Abou-Saleh).
- We will aim to feed findings to the ***NICE committee on long Covid***. Rivas is a member of long Covid practice and policy networks including a facilitator of the CHAIN long Covid collaboration.

Advocacy groups/charities such as our collaborators**:** ***resources*** they can use to support guidance and recommendations provided to other audiences.
Connecting to patients/service users, carers, focal communities, the wider public: Our PPI team with lived experience, and other stakeholders such as third sector, clinicians, social care staff, policy staff (selection to be determined in consultation with our advisory group and PPI team at start of the study), will co-create outputs to ensure their credibility and real-world relevance and to strengthen public engagement. Being online, at least initially, widens participation opportunities. Please see the description of our co-create workshops for details, p11. All data used in these workshops will have all potentially identifying details excluded. An overview of early findings will be presented to participating communities more widely via collaborator platforms, to give them the ***opportunity to reflect upon and interrogate researchers' interpretations and analysis*** of the data and ideas for outputs. This will enable broader community input into the final project outputs such as ***empowering guidance, recommendations***. All findings will be publicly available via our ***website in accessible forms*** for lay consumption using recommendations in the Patient Engagement Open forum (https://bit.ly/388SFr0) and by ***involving trusted community channels***, such as places of worship, trusted religious leaders, community champions - possibly tapping into the infrastructure developed from COVID vaccine rollout - and community groups, including collaborators Bromley-by-Bow. This aligns with Black community comments in a meeting about vaccine uptake and UK government 2021 vaccine hesitancy guidance by the Scientific Advisory Group for Emergencies ethnicity sub-group (SAGE) (https://bit.ly/38GLt6D).

OUR IMPACT AIM: We will develop policy and practice impact outputs that lead to easily adopted changes to mitigate inequities in and improve the health and social support and wellbeing of our focal group in the pandemic, pandemic recovery, its aftermath and in case of future waves and similar events. For examples of outputs please see the explanation on p15. A relevant change might be to new digital models for care that require more than one specialist to be in attendance e.g. so a local team can get complex case studies advice from a tertiary centre or a multidisciplinary team (a possibility suggested by PPI lead Camaradou). Thus we seek as **impact subaims** to:

1. provide information, guidelines and recommendations, strategies and interventions to help health and social care policymakers and practitioners to understand and address health and social care and support needs, access to resources, relevant assets and strengths, and vaccine uptake.
2. provide primary and secondary healthcare staff with personal stories of lived experience and the challenges that need to be addressed.
3. inform and empower service users through information in the public domain
4. inform further research through our findings
5. undertake relevant community and practitioner training, guidance and aspirational action plan development across health system levels (e.g. primary care, community clinics, secondary care, community-based rehabilitation and support services), initially with local impact at our 5 sites, but in the longer term with upscaling.

The charity Patient Safety's list of what is needed to achieve change in health and social care for long Covid sufferers is applicable across our participants and resembles our impact sub-aims. This demonstrates how our vision coincides with patients'/service users'. This charity also suggests the need to inform wider society to influence people's attitudes and responses, including employers, the benefits system, advice services (e.g. Citizen's Advice, Healthwatch, Patients Association). We will target some recommendations to these. Please see **Figure 1** for shorter and longer term impacts.

FURTHER FUNDING OR SUPPORT NEEDS:

We have designed this study as stand-alone; some outputs can be used at once, but some intervention suggestions would need proof-of-concept/feasibility testing and trialling. We will adapt two existing training programmes, using our new evidence, and test them during the study at the 5 WP3 sites for proof-of-concept. These would likewise need to be properly evaluated for effectiveness/efficacy.

FURTHER RESEARCH, DEVELOPMENT, ADOPTION AND IMPLEMENTATION AND IMPACT

- This is an assets/strengths-based study, which draws on existing good practice and affordances. This means relatively small changes may be made that lead to big improvements in outcome, something our WP2 work in particular should elucidate. Thus there are relatively few barriers to their development, adoption and implementation and impact. We realised when developing the study that clinicians will be unable to make major changes to their work, more so in the pandemic than ever, when resources are particularly constrained, so this was an important consideration.
- Likewise our co-production work should ensure acceptability across stakeholders, accessibility and feasibility and this is an important focus of our work.
- Our clinical co-applicants and collaborators (Dobson, Ball, Eccles, Saravanan, Abou-Saleh) will be able to adopt our recommendations and simple intervention suggestions locally, which may lead to further interest, case analyses, PDSA evaluations and other considerations and thence large-scale evaluation/upscaling for longer term impacts.
- We will be able to impact Clinical Pathways guidelines through these clinicians, supporting adoption and implementation of findings.
- ActEarly sites will be able to adopt outputs, namely East London (represented by Bromley by Bow) and Born in Bradford (represented by Dickerson).
- We will adapt 2 existing community/lay-led training programmes, and provide one course of each at the 5 WP3 sites (and consider new programmes). Further use will require commissioning.
- Policymakers have little time to assimilate information at the best of times and the rapidly changing nature of the pandemic is especially challenging. Hence we will take care to produce the type of evidence they need, including infographics, quantitative summaries and targeted qualitative approaches that link to themes relevant to policy.

PROJECT MANAGEMENT:

Monthly project management co-applicant/collaborator group meetings will be held remotely, and 4-6-monthly remote PPI and advisory group meetings involving key stakeholders, an ethnic minority clinician from the General Medical Council's Black and Minority Ethnic Doctors Forum, a co-production expert, a patient who is not in the main PPI team (tbc), a covid clinical expert, a multisystemic conditions clinical expert, and a central policymaker. We will have a 12-month independent steering committee meeting. Co-applicants/collaborators will also touch base after co-create workshops to manage outputs and impact. Microsoft Teams will be used between meetings; in the context of pandemic-related online working this is the most efficient way of managing communications as members get alerted to new content, content can be organised in channels, and communications do not get lost in a forest of emails. It also means communication is 'always on', useful in critical moments and enabling easy phone

connectivity. Weekly or as needed meetings will be held between the PI, current WP lead(s) and researchers. Lay co-researchers will be able to contact the team as and when they need, and PPI leads will determine their support and contact for study management once the study begins. The PI will be responsible for conduct of the study, day-to-day management, decision-making. But all staff will share the same duty of care to prevent unauthorised disclosure of personal information and follow good governance. A data management plan will be developed before start of the study.

PROJECT / RESEARCH TIMETABLE

	Study month									
**Task**	Pre-study	1-2	3-4	5-6	7-8	9-10	11-12	13-14	15-16	17-18
Ethics and approvals										
Set up staff										
Develop and set up survey on Redcap										
Secondary text data analysis		1-	3					14	-	17
Secondary cohort data analysis		1-	3					14	-	17
Send out survey waves and complete descriptive analyses		2-	3		8-	9			15 -16	
Three waves of qualitative research and basic analysis of these			3-4			9- 10			15 -16	
Co-design workshops										
In depth qualitative analysis (discourse, narrative)			3			-				17
Statistical modelling and social network analysis		2	-	5		9	-11		15 -	17
Key informant interviews and proof-of-concept training programmes									16	-17
Stakeholder dissemination and interim outputs for public, policy, practice										
Launch and final event										
Writing reports and papers										
Advisory group meetings										

ETHICS / REGULATORY APPROVALS

All participants will be informed about the study using a plain English statement, read to them if needed. PPI work suggested restricting interviews to English would not reduce the impact or usefulness of the study. Most research work undertaken by collaborators MedAct is in English. Moreover they suggest our research will usefully determine barriers for those who might be assumed 'OK' because they are not housed in accommodation for the vulnerable and can communicate in English. If we find this excludes intended participants, we will involve interpreters. Translated study documents/accessibility formats will be made available if needed to ensure fully informed consent.

For team, collaborator and participant safety, all activities will conform to any pandemic restriction measures in force at the time. Participants determined by the research team to be in a current crisis or situation that could be aggravated by involvement will be excluded for safety; with expertise from collaborator Prof Abou-Saleh, who undertakes migrant crisis assessments for the Helen Bamber Foundation. Our advisory group will help ensure interview and survey questions minimise participant distress, to which we will be sensitive. The core team will receive training in trauma-informed work through the KCL Violence, Abuse and Mental Health network to which the PI belongs, as many migrants have experienced considerable trauma in their lives. The survey, information sheets and interviews will all incorporate signposting to sources of help, and other resources. We avoid complex cases using exclusion criteria. Remote working has advantages with England-wide interviews, but reduces the sensitivities of face-to-face contact, which will be borne in mind. We will ensure lay researchers are well supported, with training in good governance, and that local gatekeepers do not cherry pick or exclude participants. We will pilot the interview topic guide with up to 10 people who fit the target group, using cognitive walkthrough (remotely). All sessions with participants will include a rest at 30 min and pauses as needed. Participants will be offered email as an alternative to oral sessions or to add to contributions. We use opt-in contact. Migrant status and ethnicity are protected characteristics so the study will be run via RedCap in UCL's Data Safe Haven. Anonymity and confidentiality will be stressed and carefully upheld including in photovoice work; undocumented migrants will fear arrest and deportation. We have provisional UCL ethics approval (REC 1372). All data will be held and handled in strict accordance with UCL Ethics, the UK Data Protection Act 2018, UCL data protection policies and GDPR. The lawful basis for processing personal data for GDPR will be 'public task'. The PI conducted a Data Privacy Impact Assessment (DCIA) and registered the study for this. Data will be fully anonymised, removing direct identifiers (e.g. names). No information will be released/disseminated that could lead to identification of participants including in qualitative data extracts. In line with UCL policy, electronic research data will be stored on the data server for 5 years and subsequently on external hard drives for 10 years,

converted to suitable open formats for long term preservation. Anonymised interview data will be available for secondary analyses according to the prevailing requirements of the UCL Data Safe Haven at the time requests are made.

PROJECT / RESEARCH EXPERTISE

Our team reflects the need for expertise, sensitivities and networks across ethnic/migrant groups who may otherwise be reluctant to engage, and for good pathways to policy and practice change. Some team members have personal experience of disabilities; others will enable critical distance. We have divided key contributions into specific areas below.

Methodology: PI Rivas has strong expertise across the proposed research and strong relevant networks. She was NIHR RDS London-wide qualitative methods lead 2012-2014, and has led remote qualitative pandemic research (please see her cv). She has strong expertise in impactful systematic review work (please see her cv). She works with the EPPI-Centre, with its strong links to Arms-Length Bodies. Aksoy is expert in social network analysis, and Nasim in longitudinal surveys. Thomson has a strong record of influence on policy and practice through prize-winning knowledge-exchange and co-design expertise and strong links to patient groups and charities.

Migrants: Redclift brings migrant research experience and links to Canterbury migrant groups and the Runnymede Trust. Collaborator Abou-Saleh will contribute his expertise and influence as past/current executive of WHO Biological Psychiatry, the Syrian Association for Mental Health (Syrian Refugees), Helen Bamber Foundation for assessment of refugees and asylum seekers and the Refugee Council, London. Other expertise comes from the third sector (see below).

Practitioner education: PPI member Gaulbert is a long Covid sufferer of Indian heritage and a **pain relief** therapist. She trains multidisciplinary clinicians in how to utilise pain research findings and incorporate them into their practice and will extend this within the study using our findings. We have costed in 5 training sessions to be undertaken at each of our 5 sites, or online with a capacity of 50 professionals per session. **Tough Cookie** is a Mental Resilience training programme and we will adapt this using findings, to use with community members, i.e. PwcD from ethnic minority groups.

Clinical pathways: Clinical co-applicants are well-placed not only to introduce findings into their own practice, which will be considered during our implementation pathway work e.g. in co-create workshops, but to also influence guidelines and policy. Dobson is Clinical Lead for Neurology at Barts Health, so a direct influencer of local policy and practice. Ball has a front-line leadership role with the Royal College of Obstetricians and Gynaecologists. Eccles has vast clinical expertise in chronic pain and fatigue, and strong influence on liaison psychiatry services as well as with opinion leaders in rheumatology. Saravanan is a Consultant rheumatologist with interest in hypermobility and involved in setting up one of the 40+ new long Covid clinics, in Gateshead, which provides an important route to impact. He holds North East CRN funded time for research which means he has not been costed for this study, adding to its value. Lay co-applicant Camaradou advises on a separate rehabilitation clinics project that could enable future collaboration. Bromley by Bow community group collaborators are directly connected with a transformative health centre, within the ActEarly London collaboratory. Collaborators MedAct Migrants are a group of activist doctors specialising in migrant clinical support.

Commissioning, policy, other influence: Goff has worked with the CI before, on a participatory co-developed culturally tailored diabetes education programme for those of African descent, with 2 prizes (please see the PI's cv). This is currently being rolled out in the NHS and Goff has strong links with commissioners. Diabetes is a condition of particular interest as a recognised risk factor for COVID-19 severity, a condition that may develop de novo within the long Covid complex and a condition the self-management of which could be compromised by long Covid-disrupted smell/taste (74). Lay co-applicant Parsons previously worked within NHS Southwark and Tower Hamlets Clinical Commissioning Groups, in the former as Programme Manager, and brings relevant expertise. Dickerson is Programme Manager of the UKRI ActEarly 'city collaboratory', a population-based, system-wide infrastructure for implementation in Bradford and East London; Bromley by Bow community group collaborators are also partners in ActEarly. This provides us with considerable direct access and influence e.g. Dickerson works closely with Bradford, regional and national policy and decision makers in health, education, environment and social care (75). as well as with key stakeholders nationally (including Public Health England, Department for Education and Schools, Association of Directors of Public Health) and regionally (West Yorkshire Health and Care Partnership, and Yorkshire and Humber Applied Research Collaboration). The London half of the ActEarly consortium brings similar links in the capital that we can access. Camaradou is part of the global COVID-19 END evidence synthesis and has 15 years' commercial experience in policy, stakeholder engagement partnerships, project management and

innovation R&D commercialisation roles across public and private sectors. Redclift is an academic advisor with the Runnymede Trust, the UK's leading independent race equality think tank.

Lived experience, associated networks: We have strong patient representation. Camaradou (migrant, chronic disabilities, long Covid), Kumar (immobility, vision disabilities) and collaborator Ahmed (endometriosis) are ethnic minority women with disabilities and strong third sector London networks and lead on PPI. We also have Parsons (long Covid), Gaulbert (Indian heritage, long Covid, chronic pain), Sandhu (Indian heritage, long Covid), Gabriel (young black male with long Covid). PI Rivas is a 2nd-generation Polish/Ukrainian migrant with relevant connections, and two disabled children. Aksoy, Nasim and Saravanan are from minority ethnic groups.

Third sector for nationwide recruitment and dissemination support: Collaborators include national and local migrant and disability groups, including representation from the most significant or prevalent UK chronic health conditions/disabilities, with whom team members have established strong networks. While most are third sector and impacted by the pandemic, their support will mostly involve advertisements and calls for participants on their websites and in the materials they usually distribute, so they have assured us of capacity. Existing networks e.g. Autistica, Multiple Sclerosis Society, Ehlers-Danlos Support UK, Fibromyalgia Action UK, Diabetes UK, British Psycho-oncology Soc, Endometriosis UK, British Lung Foundation-Asthma UK **have all provided letters of support**. We will also contact MIND, RNIB, Deaf Society UK, Versus Arthritis, MND Association, Beat, Parkinson's Society, Scope, Alzheimer's UK, Epilepsy Society, British Heart Foundation. Local organisations at sites (e.g. Kent Refugee Help). MedAct Migrant Subgroup is costed in for recruitment support.

Researchers: Nasim and Oksay will line manage one 18-month 100%FTE quantitative researcher and Rivas will line manage two 100%FTE qualitative researchers, one for 18 months, one for 10 months, all based at UCL. See Justification of costs in the main form for training/support details.

MAIN INPUTS	ACTIVITIES	KEY OUTPUTS (see case for support text for more details)	KEY OUTCOMES/ IMPACT
**Clinical and policy pathways**<br>Ball, Dobson, Goff,, Saravanan, Eccles, collaborators Abou-Saleh, Bromley-by-Bow, ActEarly<br>**Methodology expertise**:<br>*Qualitative and participatory research:* Thomson, Rivas, Redclift, Bromley-by-Bow collaborators<br>*Networks, Surveys:* Aksoy, Nasim, Rivas<br>**Migrants/BAME:** Redclift (on Runnymede Trust Ac Forum), Goff, Rivas, collaborators MedAct, Bromley-by-Bow, Abou-Saleh<br>**PPI:** Kumar, Ahmed, Gaulbert Camaradou, Parsons, Gabriel<br><br>**Chronic conditions/disabilities:** Collaborators: Autistica, Multiple Sclerosis Society, Ehlers-Danlos Support UK, Fibromyalgia Action UK, Diabetes UK, British Psycho-oncology Society, Endometriosis UK, British Lung Foundation-Asthma UK, MedAct have agreed to advertise for participants.<br><br>We will also contact MIND, RNIB, Deaf Society UK, Versus Arthritis, Motor Neurone Disease Association, Beat (UK eating disorder charity), Parkinson's Society, Alzheimer's UK, Epilepsy Society, British Heart Foundation.<br><br>**Advisory group**: key stakeholders<br>**Prior and new community engagement work**<br>**Tested participatory approaches during COVID-19** and associated expertise/resources<br>**Existing research-ready infrastructures** including ActEarly,<br>**UCL panel and cohort data**<br>**Born in Bradford and East London survey (ActEarly)**<br>**Lay co-researchers**<br>**Long covid**<br><br>**Pandemic-related changes**	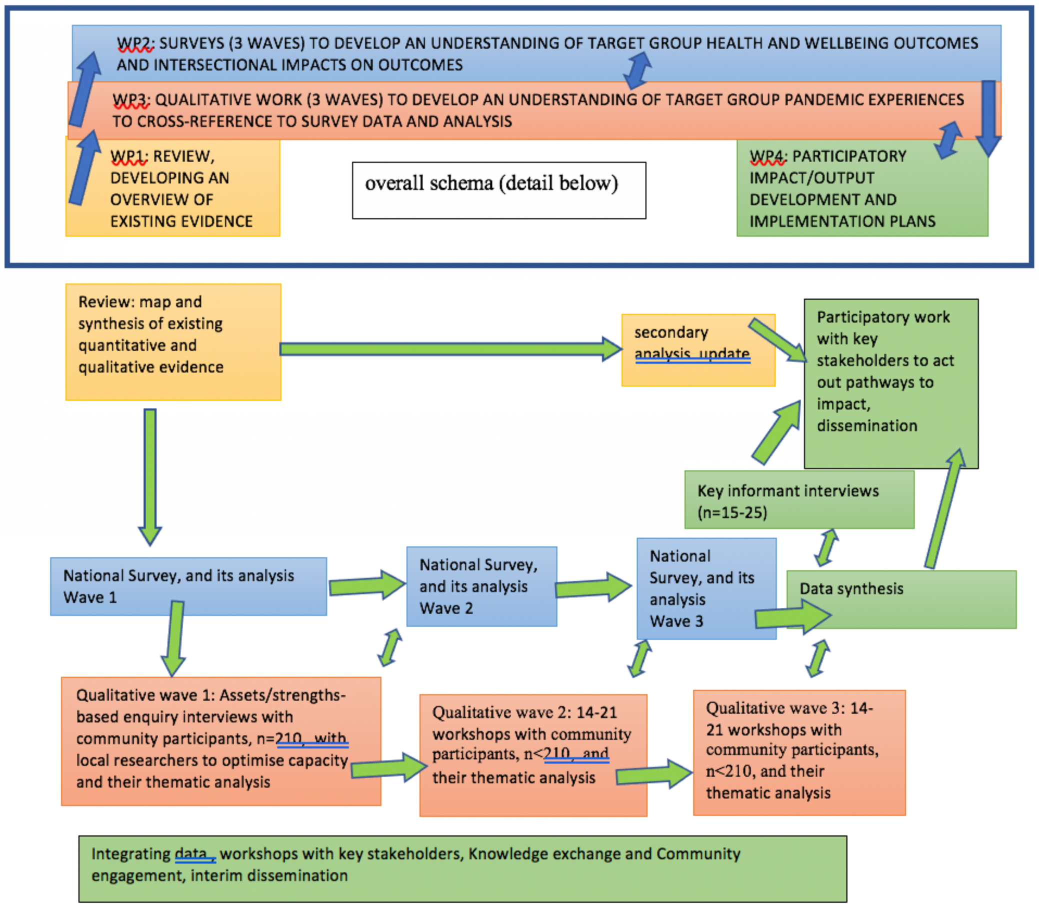<br><br><br>**Dissemination/ policy outputs**<br>**Impact platforms:** collaborator Abou-Saleh, third sector collaborators, other team networks, clinical co-As/ collaborators<br>**Policy and Clinical direct pathways:** Ball, Dobson, Goff, Abou-Saleh, Saravanan, Eccles, Bromley-by-Bow, ActEarly	Network, asset, strengths and pandemic response analyses<br><br>A toolbox of co-created strategies/ guidance/recommendations/intervention ideas<br><br>Implementation analysis for decision-makers and practitioners<br><br>Contributions to the new National Plan for Neurology (Dobson); UCL/QMUL/Bromley-by-Bow ActEarly site direct paths to policy/practice; WHO (through Abou-Saleh), Ball Royal College/clinical pathways<br><br>NIHR and other reports and disseminations<br><br>Policy: guidance, policy briefs, policy/arms-length-body meetings, All-Parliamentary-Government-Group-on-Coronavirus<br><br>KNOWLEDGE AND EVIDENCE	**Early/medium (duration of project):** Locally improved access to and use of appropriate support, health and social care and resources for chronic conditions/ disabilities including long covid for BAME groups.<br><br>Enhanced wellbeing, coping, mental and physical health<br><br>Influence on pandemic local, national policy, practice and decision making<br>Better-informed pandemic policy and practice<br><br>**Later (during and in the year after the project):** National scale-up<br><br>Empowered communities<br><br>Reinforced assets and strengths<br><br>Health/social care services job satisfaction<br><br>Enhanced relationships between services and communities

References

1. Loubaba M, Jones T. The impact of COVID-19 on black, Asian and minority ethnic communities, NIHR special report, 20/05/2020, UoB_COVID19004
2. Orcutt M, Spiegel P, Kumar B, Abubakar I, Clark J, Horton R. Lancet Migration: global collaboration to advance migration health. *Lancet* 2020; 395:317–19.
3. Kuper H, Banks LM, Bright T et al. Disability-inclusive COVID-19 response. *Wellcome Open Res* 2020, 5:79
4. Abuelgasim SE, Saw L.J, Shirke M. , Zeinah M. , Harky A. . COVID-19: unique public health issues facing Black, Asian and minority ethnic communities. *Curr Probl Cardiol*, 2020, 45, p. 100621
5. Marmot Michael, Allen Jessica, Goldblatt Peter, Herd Eleanor, Morrison Joana. Build Back Fairer: The COVID-19 Marmot Review. UCL Institute of Health Equity, London, Decem,eber 2020
6. Mathur R, Rentsch CT, Morton C, Hulme WJ, Schultze A, MacKenna B, Eggo RM, Bhaskaran K, Wong AYS, Williamson EJ, Forbes H, Wing K, McDonald HI, Bates C, Bacon S, Walker AJ, Evans D, Inglesby P, Mehrkar A, Curtis HJ, DeVito NJ, Croker R, Drysdale H, Cockburn J, Parry J, Hester F, Harper S, Douglas IJ, Tomlinson L, Evans S, Grieve R, Harrison D, Rowan K, Khun K, Chaturvedi N, Smeeth L, Goldacre B. Ethnic differences in COVID-19 infection, hospitalisation, and mortality: an OpenSAFELY analysis of 17 million adults in England. medRxiv 2020;doi:10.1101/2020.09.22.20198754
7. Mirza M., Unmet needs and diminished opportunities: disability, displacement and humanitarian healthcare, Research report 212, 2011, UNHCR: Geneva
8. Clarke, S.K., Kumar, G.S., Sutton, J. *et al.* Potential Impact of COVID-19 on Recently Resettled Refugee Populations in the United States and Canada: Perspectives of Refugee Healthcare Providers. *J Immigrant Minority Health* (2020). https://doi.org/10.1007/s10903-020-01104-4
9. Putz C, Ainsl D. ONS Coronavirus (COVID-19) related deaths by disability status, England and Wales: 2 March to 14 July 2020
10. Hankivsky Olena, Kapilashrami Anuj, Intersectionality offers a radical rethinking of covid-19 *BMJ.* May 15, 2020
11. Anderson B. *Citizenship: What is it and why does it matter?* https://migrationobservatory.ox.ac.uk/resources/primers/citizenship-what-is-it-and-why-does-it-matter/
12. NIHR. Living with covid-19. A dynamic review of the evidence around ongoing covid-19 symptoms (often called long covid). October 2020. https://evidence.nihr.ac.uk/themedreview/living-with-covid1
13. NHS England (2020) *After-care needs of inpatients recovering from COVID-19* www.england.nhs.uk/coronavirus/publication/after-care-needs-of-inpatients-recovering-from-COVID-19/
14. The Office for National Statistics (ONS). The prevalence of long COVID symptoms and COVID-19 complications.16 December 2020 https://www.ons.gov.uk/news/statementsandletters/theprevalenceoflongcovidsymptomsandcovid19complications[accessed dec 18 2020]
15. Harding S, Balarajan R. Limiting long-term illness among Black Caribbeans, Black Africans, Indians, Pakistanis, Bangladeshis and Chinese. *Ethnicity & Health*,2000;5,41-46
16. Razaq A et al. *BAME COVID-19 deaths – What do we know?* Rapid Data & Evidence Review. Centre for Evidence-Based Medicine, Oxford, May 2020
17. Sheridan Rains, L., Johnson, S., Barnett, P. *et al.* Early impacts of the COVID-19 pandemic on mental health care and on people with mental health conditions: framework synthesis of international experiences and responses. *Soc Psychiatry Psychiatr Epidemiol* (2020). https://doi.org/10.1007/s00127-020-01924-7
18. Frank P, Iob E, Steptoe A, Fancourt D. Trajectories of depressive symptoms among vulnerable groups in the UK during the COVID-19 pandemic. *medRxiv* 2020.06.09.20126300
19. Gholami R. Coronavirus: Social distancing.*The Conversation* 6/4/2020.
20. PHE. Beyond the data: Understanding the impact of COVID-19 on BAME groups. June 2020 PHE publications, UN gateway number: GW-1307
21. Hombrados-Mendieta I et al. Positive influences of social support on sense of community, life satisfaction and health of immigrants. Front Psychol 2019;10:2555.
22. Iyengar, K., Jain, V. K., & Vaishya, R. Pitfalls in telemedicine consultations in the era of COVID 19 and how to avoid them. *Diabetes & metabolic syndrome*, 2020,*14*(5), 797–799. Advance online publication. https://doi.org/10.1016/j.dsx.2020.06.007
23. Cavagna L, Zanframundo G, Codullo V, Pisu MG, Caporali R, Montecucco C. Telemedicine in rheumatology: a reliable approach beyond the pandemic. Rheumatology (Oxford). 2020 Sep 7:keaa554. doi: 10.1093/rheumatology/keaa554. Epub ahead of print. PMID: 32893293; PMCID: PMC7499691.
24. ISARIC Global COVID-19 follow up study protocol v. 1.0 17 Nov. 2020 1 *ISARIC: International Severe Acute Respiratory and emerging Infection Consortium www.isaric.og What is the recovery rate and risk of long-term

consequences following a diagnosis of COVID19? – a harmonised, global longitudinal observational study protocol. Protocol registration number: osf.io/c5rw3/ version: 1.0 17 November 2020 EuroQol ID: 37035
25. Fang ML, Sixsmith J, Lawthom R. et al. Experiencing 'pathologized presence and normalized absence'. BMC Public Health 2015;15:923
26. NICE, RCGP and SIGN: Covid-19 guideline scope: management of the long-term effects of COVID-19, October 2020 tinyurl.com/NICE-postcovid19
27. Dennis, Andrea, Wamil, Malgorzata, Kapur, Sandeep et al. Multi-organ impairment in low-risk individuals with long COVID. medRxiv 2020.10.14.20212555; doi:https://doi.org/10.1101/2020.10.14.20212555
28. Baptist, Alan P. Desmond Lowe, Nadeen Sarsour, Hannah Jaffee, Sanaz Eftekhari, Laurie M. Carpenter, Priya Bansal. Asthma Disparities During the COVID-19 Pandemic: A Survey of Patients and Physicians. The Journal of Allergy and Clinical Immunology: In Practice, 8, Issue 10, 2020, Pages 3371-3377.e1,ISSN 2213-2198
29. Campos-Castillo, Celeste, Denise Anthony, Racial and ethnic differences in self-reported telehealth use during the COVID-19 pandemic: a secondary analysis of a US survey of internet users from late March, *Journal of the American Medical Informatics Association*, , ocaa221, https://doi.org/10.1093/jamia/ocaa221
30. Slewa-Younan S, Uribe Guajardo MG, Heriseanu A et al. A systematic review of Post-traumatic Stress Disorder and depression amongst Iraqi refugees located in Western countries. J Immigrant Minority Health 17 2015;1231–1239
31. Harris J. 'All Doors are Closed to Us', Disability & Society 2003;18:395-410
32. Ottosdottir G, Evans R. Ethics of care in supporting disabled forced migrants. British Journal of Social Work 2014; 44:suppl 1,i53-i69.
33. Sandhu P, Ibrahim J, Chinn D. 'I wanted to come here because of my child' J Applied Res in Intellectual Dis 2017;30:371-382
34. Yeo R. The deprivation experienced by disabled asylum seekers in the UK. Disability & Soc 2017;32:657–77
35. Quinn N. Participatory action research with asylum seekers and refugees experiencing stigma and discrimination. Disability & Soc 2014;29:58-70
36. Goff, L. M., Moore, A. P., Rivas, C., & Harding, S. (2019). Healthy Eating and Active Lifestyles for Diabetes (HEAL-D) BMJ Open, 9 (2), e023733
37. Rivas C, Kelly M, Seale C. The interpreted diabetes consultation. Journal of Diabetes Nursing 2014: 18: 422–4 (First publ. in Diabetes & Primary Care 2013 16: 31–3)
38. Mehta B, Jannat-Khah D, Fontana MA*, et al.* Impact of COVID-19 on vulnerable patients with rheumatic disease: results of a worldwide survey. *RMD Open* 2020;6:e001378. doi: 10.1136/rmdopen-2020-001378
39. Boldrini P, Garcea M, Brichetto G, Reale N, Tonolo S, Falabella V, Fedeli F, Cnops AA, Kiekens C. Living with a disability during the pandemic. Eur J Phys Rehabil Med. 2020 Jun;56(3):331-334. doi: 10.23736/S1973-9087.20.06373-X. Epub 2020 May 14. PMID: 32406226.
40. Cheong, J.L.-Y., Goh, Z.H.K., Marras, C., Tanner, C.M., Kasten, M., Noyce, A.J. and (2020), The Impact of COVID-19 on Access to Parkinson's Disease Medication. Mov Disord. https://doi.org/10.1002/mds.28293
41. Al-Hashel, J.Y., Ismail, I.I. Impact of coronavirus disease 2019 (COVID-19) pandemic on patients with migraine: a web-based survey study. *J Headache Pain* 21, 115 (2020). https://doi.org/10.1186/s10194-020-01183-6
42. Ziadé, N, el Kibbi, L, Hmamouchi, I, et al. Impact of the COVID-19 pandemic on patients with chronic rheumatic diseases: A study in 15 Arab countries. *Int J Rheum Dis*. 2020; 23: 1550– 1557.
43. Baranidharan G, Bretherton B, Eldabe S, et al. The impact of the COVID-19 pandemic on patients awaiting spinal cord stimulation surgery in the United Kingdom: a multi-centre patient survey. *British Journal of Pain*. August 2020. doi:10.1177/2049463720948092
44. Umucu E, Tansey TN, Brooks J, Lee B. The Protective Role of Character Strengths in COVID-19 Stress and Well-Being in Individuals With Chronic Conditions and Disabilities: An Exploratory Study. *Rehabilitation Counseling Bulletin*. October 2020. doi:10.1177/0034355220967093
45. Ciaffi, J., Brusi, V., Lisi, L. *et al.* Living with arthritis: a "training camp" for coping with stressful events? A survey on resilience of arthritis patients following the COVID-19 pandemic. *Clin Rheumatol* 39, 3163–3170 (2020). https://doi.org/10.1007/s10067-020-05411-x
46. Brewer G. & K. Stratton (2020) Living with Chronic Fatigue Syndrome during lockdown and a global pandemic, Fatigue: Biomedicine, Health & Behavior, 8:3, 144-155, DOI: 10.1080/21641846.2020.1827503
47. Theoharides, TC. COVID-19, mast cells and beneficial effects of luteolin. BioFactors. 2020 May; in press.
48. NHS England. Integrating care. Next steps to building strong and effective integrated care systems across England. NHS England, December 2020.

49. Keith RE, Crosson JC, O'Malley AS. *et al.* Using the Consolidated Framework for Implementation Research (CFIR) to produce actionable findings: a rapid-cycle evaluation approach to improving implementation. *Implementation Sci* 2017,12, 15. https://doi.org/10.1186/s13012-017-0550-7
50. Pereira A. Long-Term Neurological Threats of COVID-19 Front Neurol. 2020;11:308
51. Oliver Mike (2013) The social model of disability: thirty years on, Disability & Society, 28:7, 1024-1026, DOI: 10.1080/09687599.2013.818773
52. Bronfenbrenner U. Toward an experimental ecology of human development. Am Psych 1977;32:513-531
53. Glover RE, van Schalkwyk MC, Akl EA, Kristjannson E, Lotfi T, Petkovic J, Petticrew MP, Pottie K, Tugwell P, Welch V, A framework for identifying and mitigating the equity harms of COVID-19 policy interventions, *J Clin Epidemiol*, 2020, doi: https://doi.org/10.1016/ j.jclinepi.2020.06.004.
54. Srijith PK, Hepple M, Bontcheva K & Preotiuc-Pietro D (2017) Sub-story detection in Twitter with hierarchical Dirichlet processes. *Information Processing & Management*, 53(4), 989-1003.
55. Wright J, Hayward AC, West J *et al.* ActEarly: a City Collaboratory approach to early promotion of good health and wellbeing [version 1; peer review: 2 approved]. *Wellcome Open Res* 2019, 4:156 (https://doi.org/10.12688/wellcomeopenres.15443.1)
56. Dickerson J, Kelly B, Lockyer B *et al.* Experiences of lockdown during the Covid-19 pandemic: descriptive findings from a survey of families in the Born in Bradford study [version 1; peer review: 1 approved, 1 approved with reservations]. *Wellcome Open Res* 2020, 5:228 (https://doi.org/10.12688/wellcomeopenres.16317.1)
57. Aksoy, Ozan, David Bann, Meg E Fluharty, Alita Nandi. Religiosity and mental wellbeing among members of majority and minority religions: findings from Understanding Society, The UK Household Longitudinal Study. medRxiv 2020.02.25.20027904; doi:https://doi.org/10.1101/2020.02.25.20027904
58. WHO QOL DISABILITIES Group. WHO QOL Disabilities. 2011; WHO, Geneva
59. NHCR. Vulnerability Assessment Framework Questionnaire Validation Workshop Summary 2016, UNHCR:Geneva https://bit.ly/2YQ1G4P [accessed 30 June 2020]
60. Stansfeld, Stephen, Michael Marmot. Deriving a survey measure of social support: The reliability and validity of the close persons questionnaire, Social Science & Medicine, Volume 35, Issue 8,1992, Pages 1027-1035,
61. Sharma VK et al. The Global Mental Health Assessment Tool--Primary Care Version (GMHAT/PC). Development, reliability and validity. World Psychiatry. 2004;3(2):115-119
62. Golnar A-A et al. *Lancet Psych* 2019;6(3):257-266
63. Sutherland C, Cheng Y. Participatory-action research with (im)migrant women in two small Canadian cities. J Immigr Refug Stud 2009;7:290–307
64. Rivas C, Sohanpal R, MacNeill V, *Steed L, Edwards E, Griffiths C, Eldridge S, Taylor SJC, Walton R*. Determining counselling communication strategies associated with successful quits in the National Health Service community pharmacy Stop Smoking programme in east London: a focused ethnography using recorded consultations. *BMJ Open,* Sept 2017
65. Engman Athena, Embodiment and the foundation of biographical disruption, Social Science & Medicine, Volume 225, 2019, Pages 120-127, ISSN 0277-9536, https://doi.org/10.1016/j.socscimed.2019.02.019.
66. Oteros-Rozas, E., B. Martín-López, T. Daw, E. L. Bohensky, J. Butler, R. Hill, J. Martin-Ortega, A. Quinlan, F. Ravera, I. RuizMallén, M. Thyresson, J. Mistry, I. Palomo, G. D. Peterson, T. Plieninger, K. A. Waylen, D. Beach, I. C. Bohnet, M. Hamann, J. Hanspach, K. Hubacek, S. Lavorel and S. Vilardy 2015. Participatory scenario planning in place-based social-ecological research: insights and experiences from 23 case studies. Ecology and Society 20(4):32. http://dx.doi.org/10.5751/ES-07985-200432
67. Booth A, Noyes J, Flemming K*, et al.* Formulating questions to explore complex interventions within qualitative evidence synthesis. *BMJ Global Health* 2019;**4:**e001107.
68. Wolf EJ, Harrington KM, Clark SL, Miller MW. Sample Size Requirements for Structural Equation Models: An Evaluation of Power, Bias, and Solution Propriety. *Educ Psychol Meas*. 2013;76(6):913-934.
69. Calderwood Lisa. Reducing non-response in longitudinal surveys by improving survey practice,PhD, 1 Jun 2016
70. Enders, C.K., 2010. *Applied missing data analysis*. Guilford press.
71. Edwards A Refugee and migrant rights. https://www.unhcr.org/uk/news/latest/2016/7/ 55df0e556/unhcr-viewpoint-refugee-migrant-right.html 2016 (accessed 15 June 2020).
72. The Migration Observatory. *Migrants in the UK: An overview. 2019*, The Migration Observatory: Oxford https://bit.ly/2VydjeE [accessed 30 June 2020]
73. Downing A, et al. Protocol for a UK-wide patient-reported outcomes study*, BMJ Open* 2016;6:e013555.

74. Alyammahi, Shatha K., Shifaa M.Abdin, Dima W.Alhamad, Sara M.Elgendy, Amani T.Altell,Hany A.Omar, The dynamic association between COVID-19 and chronic disorders: An updated insight into prevalence, mechanisms and therapeutic modalities. Infection, Genetics and Evolution, Volume 87, January 2021, 104647
75. McEachan RRC, Dickerson J, Bridges S *et al.* The Born in Bradford COVID-19 Research Study: Protocol for an adaptive mixed methods research study to gather actionable intelligence on the impact of COVID-19 on health inequalities amongst families living in Bradford [version 1; peer review: 3 approved]. *Wellcome Open Res* 2020, 5:191 (https://doi.org/10.12688/wellcomeopenres.16129.1)

NIHR132914 - Dr Carol Anne Rivas - Institute of Education, University of London. Response to Funding Committee; Lay summary

Lay summary

Many people from Black, Asian and Minority Ethnic groups (BAME), especially those with underlying (chronic) conditions/disabilities, face barriers to accessing networks of appropriate support, health and social care or vital ‘resources’, such as medicine and food. Around 50% lived in poverty in 2019; the pandemic has worsened their plight, highlighting the need for these barrriers to be removed. To determine where and how best to intervene to ensure this, we need to understand the problems - and successes - these groups have experienced, especially as health and social care tries to return to normal. We also need to understand what affects their vaccine uptake and if pandemic-related service changes that are retained further exclude them. Our approach is 'intersectional'. This means we recognise everyone is affected differently by the pandemic, according to the intersection (interplay) of factors such as ethnicity, citizenship, age, gender, their work, and health or disability.

To understand this complex situation, we will survey 4,000 UK BAME people and for contrast 1000 white British, 3 times over 15 months. We will compare their health, social networks (who they have contact with) and how these help or hinder them, ways they cope with pandemic changes and associated access to support, care and resources. We will consider how various intersectional factors affect this.

After Survey 1 we will interview 210 more people in 5 diverse sites in England about the same topics, probing for coping strategies and ideas to inform health and social care policy and practice. Interviewees will also describe their networks using special brief questionnaires, photos and maps. We use remote working. We will find people for the study via social media, NHS clinics, charities, special patient and migrant groups, our own networks, and large databases of adults interested in health research across the UK.

We will focus on migrants from the Middle East, India, Pakistan, Poland or Africa, or whose parents were born there, as the most likely to have problems (e.g. to have limited citizenship rights or to die from COVID-19). We will look at the impact of also having a chronic condition/disability including 'long covid'. Our PPI leads will train local community members to do some of the research.

After surveys 2 and 3, interviewees will be invited to workshops to discuss findings and more recent changes. We will also review published and informal (e.g. blog) articles about pandemic BAME and disability experiences, and data from other complementary COVID-19 surveys. All our findings will be combined. Over the 18-month study we will hold 5 participatory sessions with members of different BAME communities and key informants working together to help analyse our data and co-design solutions to issues, pragmatically including ‘life hacks’ and service adaptations for rapid impact. At 16 months we will interview 15-25 key informants such as support staff and community leaders to help us put our work into immediate practice.

Deleted: before
Deleted: the pandemic
Deleted: . T
Deleted: which has
Deleted: ed
Deleted: a pandemic legacy of
Deleted: improved access to appropriate support, health and social care for these groups
Deleted: those affected by the pandemic and
Deleted: where they live,
Deleted: issues
Deleted: ¶
Deleted: continuing changes in 'normality'
Deleted: ¶
Deleted: be
Deleted: augmented with
Deleted: and exploration of
Deleted: about participant networks
Deleted: .¶
Deleted: ¶
Deleted: interview
Deleted: and analyse
Deleted: , and combine these with our other findings
Deleted: mixed stakeholder
Deleted: health/care delivery

Findings and solutions will be shared as they emerge at each of the 3 data waves, for early benefit. We will report changes over time in experiences, outcomes and solutions and consider how to apply our work across the UK. We aim for immediate, readily implemented, relevant useful change in UK pandemic health and social care service delivery. We include training outputs, strong networks and Co-As with direct influence on policy and practice.

CO-APPLICANT INFORMATION

Co-applicant Information

**Name**	Dr Victoria Redclift
**Role and organisation**	Associate Professor of Political Sociology
Department	Social Research Institute
Organisation	Institute of Education, University College London
Email	v.redclift@ucl.ac.uk

Co-applicant Information – Qualifications

Degree/subject professional Qualification	Awarding body, date of award
PGCHE - Postgraduate Certificate in Higher Education	Higher Education Academy - 28/06/2012
PhD - Sociology	University of London - 01/09/2011
MSc - Population and Development	London School of Economics - 28/09/2006
BA (Hons) - Ancient History	University of Bristol - 21/06/2002

Patient/Service user or carer applicants

Patient / service users or carer applicants information

RECENT PUBLICATIONS AND RESEARCH GRANTS

**Commitment to this Research Project**	5 % FTE

Recent Relevant Publications

Books

Redclift, V., (2013). Statelessness and citizenship: Camps and the creation of political space, London: Routledge. ISBN 978-0415631358

Shortlisted for the BSA Philip Abrams Memorial Prize.

Edited Books

James, T.M, Redclift, V and H. Kim (Eds) (2015) New Racial Landscapes: Contemporary Britain and the Neoliberal Conjuncture. London: Routledge. ISBN 978-1138795570

Journal articles and book chapters

Redclift, V., and Rajina, F (2019) 'The hostile environment, Brexit and 'reactive' or 'protective' transnationalism', Global Networks.

Redclift, V., (2018) 'Re-bordering camp and city: 'Race', space and citizenship in Dhaka', The Sage Handbook of the 21st Century City. London: Sage.

Redclift, V. and Rajina, F (2017), 'Rethinking Muslim Migration: Frameworks, flux and fragmentation', Ethnic and Racial Studies Review, 40 (3)

Redclift, V., (2017), 'The de-mobilization of diaspora: History, memory and 'latent identity'', Global Networks, 17 (4)

Research Grants Held

UCL Health of the Public Grant - £17,596.15

January - July 2021

'Multiple Minority Identities and Mental Health - A Mixed method approach to addressing health inequalities'

ESRC Future Research Leaders [ES/N000986/1] - £216,995

2016-2020 - current (until 30/12/20)

'Transnational Practices in Local Settings: Experiences of citizenship among Bangladesh-origin Muslims in London and Birmingham'

Philip Leverhulme Prize [PLP-2014-221] - £100,000

2015-2021 - current (until 31/03/21)

'From Brick Lane to Little Bangladesh: Transnational Political Space in London and Los Angeles'

British Academy/Leverhulme Small Research Grant [SG121376] - £10,000

2013-2015<br>‘Intra-minority relations and transnational political space: North Indian Muslims in the UK and the US’

NIHR | National Institute for Health Research

NIHR Health Services and Delivery Research Programme

CO-APPLICANT INFORMATION

Co-applicant Information

**Name**	Dr Ruth Dobson
**Role and organisation**	
Department	
Organisation	The Barts and The London School of Medicine and Dentistry
Email	ruth.dobson@qmul.ac.uk

Co-applicant Information – Qualifications

**Degree/subject professional Qualification**	**Awarding body, date of award**
PhD - Neuroscience	University of London - 04/10/2013
Member - MRCP	Royal College of Physicians of London - 05/03/2007
MBBS - Medicine	UCL - 05/07/2004
MA (Hons) - Social Anthropology	University of Cambridge - 02/07/2001

Patient/Service user or carer applicants

**Patient / service users or carer applicants information**

RECENT PUBLICATIONS AND RESEARCH GRANTS

Commitment to this Research Project	5 % FTE

Recent Relevant Publications

Pakpoor J, Schmierer K, Cuzick J, Giovannoni G, and Dobson R: "Estimated and projected burden of multiple sclerosis attributable to smoking and childhood and adolescent high body-mass index: a comparative risk assessment" – Int J Epidemiol 2020 Aug 26 epub ahead of print (DoI 10.1093/ije/dyaa151)

Jacobs B, Taylor T, Awad A, Baker D, Giovanonni G, Noyce AJ, and Dobson R: "Summary-data-based mendelian randomisation prioritises potential druggable targets for Multiple Sclerosis" – Brain Commun. 2020 Aug 14;2(2):fcaa119.

Dobson R, Jitlal M, Marshall C, Noyce A, Cuzick J and Giovannoni G: "Ethnic and socioeconomic associations with multiple sclerosis risk" - Ann Neurol. 2020;87(4):599-608.

Dobson R, Dassan P, Roberts M, et al: "UK Consensus on pregnancy in multiple sclerosis – ABN guidelines" – Practical Neurology 2019 Apr;19(2):106-114 (updated December 2019)

Dobson R, Cock H, Brex P and Giovannoni G: "Vitamin D supplementation: how to do it" – Practical Neurology 2018 Feb;18(1):35-42

Ramagopalan SV, Dobson R, Meier UC and Giovannoni G: "Multiple Sclerosis: Prodromes, Endophenotypes and Causal Pathways" – Lancet Neurology, 2010 Jul;9(7):727-39.

Research Grants Held

Developing a UK MS Pregnancy Register (current) – Horne Family Charitable Foundation - £135,000 (Chief Investigator)

Assessing the vitamin D status of the UK MS population (October 2018 – October 2019) – MS Society - £150,000 (Chief Investigator)

Famciclovoir as an anti-EBV treatment in MS (current) – Horne Family Charitable Foundation - £125,000 (Chief Investigator)

A prospective, real-world pharmacovigilance study in MS (OPTIMISE-MS) (current) – Biogen/Merck/Celgene - £7,000,000 (Principal Investigator; Chief Investigator Prof. P. Matthews)

Towards an endophenotype in MS (2010 – 2013) – MS Society - £250,000

CO-APPLICANT PPI - INFORMATION

Co-applicant PPI Information

**Name**	Dr Sarabajaya Kumar
**Role and organisation**	Lecturer (teaching)
Department	Political science
Organisation	University College London
Email	sarabajaya.kumar@ucl.ac.uk

Co-applicant PPI Information – Qualifications

Degree/subject professional Qualification	Awarding body, date of award
PhD - Accountability (Public Policy and Management)	Aston University, Birmingham - 01/07/2003
MSc - Public Sector Management	Aston University, Birmingham - 01/10/1988
BA (Hons) - Sociology and Religion	University of London Goldsmiths' College - 01/10/1985

Patient/Service user or carer applicants

Patient / service users or carer applicants information
I have a PhD in accountability to health and welfare service users as well as lived experience of using health and welfare services due to my complex hereditary medical conditions. I am an experienced qualitative researcher and have completed several research projects using qualitative methods. I am an expert patient and active in multiple patient networks.

RECENT PUBLICATIONS AND RESEARCH GRANTS

**Commitment to this Research Project**	5 % FTE

**Recent Relevant Publications**

**Research Grants Held**
1) International Comparative Project of Disabled Students Experiences of Covid-19. Lead applicant: Sarabajaya Kumar, UCL Strategic Fund Global Engagement Office. Sept 2020 - July 2021. £12,000<br><br>2) Ableism in the Labour Market. Lead applicant: Sarabajaya Kumar, The Association of Disabled Professionals. June 2020 - November 2021. £44,000

NIHR | National Institute for Health Research

NIHR Health Services and Delivery Research Programme

CO-APPLICANT INFORMATION

Co-applicant Information

**Name**	Dr Ozan Aksoy
**Role and organisation**<br>***Department***<br>***Organisation***<br>***Email***	 <br>Institute of Education, University College London<br>ozan.aksoy@ucl.ac.uk

Co-applicant Information – Qualifications

Degree/subject professional Qualification	Awarding body, date of award
PhD - Quantitative Sociology	Utrecht University - 30/08/2013
MSc - Sociology and Social Research	Utrecht University - 30/06/2007
BA (Hons) - Business Administration and Management Science	Bogazici University - 30/07/2004

Patient/Service user or carer applicants

Patient / service users or carer applicants information

RECENT PUBLICATIONS AND RESEARCH GRANTS

**Commitment to this Research Project**	20 % FTE

Recent Relevant Publications

Aksoy, O., Bann, D., Fluharty, E.M. & Nandi, A. (2020) Religiosity and mental wellbeing among members of majority and minority religions: findings from Understanding Society, forthcoming in American Journal of Epidemiology, preprint at: https://www.medrxiv.org/content/10.1101/2020.02.25.20027904v1

Aksoy, O. & Gambetta, D. (2020) The politics behind the veil, forthcoming in European Sociological Review, preprint at: https://osf.io/preprints/socarxiv/ezpaq/.

Przepiorka, W. & Aksoy, O. (2020) Does herding undermine the trust enhancing effect of reputation? An empirical investigation with online-auction data. Social Forces (online first) https://doi.org/10.1093/sf/soaa057.

Aksoy, O. (2020) Social identity and social value orientations, forthcoming in Buskens, V., Corten, R. & Snijders, C. Ed. Advances in the Sociology of Trust and Cooperation, De Gruyter-Oldenbourg, preprint and replication material at: https://osf.io/preprints/socarxiv/83rzv/.

Aksoy, O. (2019) Crosscutting circles in social dilemmas: effects of social identity and inequality on cooperation. Social Science Research, 82: 148-163, preprint and replication material at: https://osf.io/preprints/socarxiv/ah3g2/.

Aksoy, O. & Billari, C. F. (2018) Political Islam, marriage, and fertility: Evidence from a natural experiment. American Journal of Sociology 123(5): 1296-1340.

Research Grants Held

2020 – 2022: British Academy/Leverhulme Small Research Grant: “Text-as-data analysis of the content and consequences of Turkey's Friday Khutbas”, PI (£5.692)

2013 – 2015: RUBICON grant by the Netherlands Organization for Scientific Research (NWO): “Understanding modern societal problems: The effects of diversity, inequality, and segregation on social cohesion”, PI (€163.000)

NIHR | National Institute for Health Research

NIHR Health Services and Delivery Research Programme

CO-APPLICANT INFORMATION

Co-applicant Information

**Name**	Dr Josie Dickerson
**Role and organisation**	Research Director
Department	Born in Bradford
Organisation	Bradford Teaching Hospitals NHS Foundation Trust
Email	josie.dickerson@bthft.nhs.uk

Co-applicant Information – Qualifications

Degree/subject professional Qualification	Awarding body, date of award
D Phil - Psychology	University of York - 01/09/2001
BSc (Hons) - Psychology	University of Liverpool - 30/06/1998

Patient/Service user or carer applicants

Patient / service users or carer applicants information

RECENT PUBLICATIONS AND RESEARCH GRANTS

Commitment to this Research Project	0.1 % FTE

Recent Relevant Publications

Dickerson J, Kelly B, Lockyer B et al., 'When will this end? Will it end?' The impact of the March-June 2020 UK Covid-19 lockdown response on mental health: a longitudinal survey of mothers in the Born in Bradford study. medRxiv 2020.11.30.20239954; https://medrxiv.org/cgi/content/short/2020.11.30.20239954v1

Dickerson J, Kelly B, Lockyer B et al. Experiences of lockdown during the Covid-19 pandemic: descriptive findings from a survey of families in the Born in Bradford study [version 1; peer review: 1 approved]. Wellcome Open Res 2020, 5:228 (https://doi.org/10.12688/wellcomeopenres.16317.1)

Dickerson, J, Bird PK, Bryant M, et al. Integrating research and system-wide practice in public health: lessons learnt from Better Start Bradford BMC Public Health. 2019 DOI 10.1186/s12889-019-6554-2

Dickerson J. Bird P, McEachan RRC, et al. Born in Bradford's Better Start: an experimental birth cohort study to evaluate the impact of early life interventions. BMC Public Health, 2016, 16(1), pp.1–14. Available at: http://dx.doi.org/10.1186/s12889-016-3318-0.

Bowyer-Crane C, Nielsen D, Bryant M, Heald R, Storr C, Dickerson J. The oTTer project - A feasibility and process evaluation protocol of Talking Together. Pilot and Feasibility Studies. 2019: 119.

Islam S, Small N, Bryant M, Yang T, Cronin de Chavez A, Saville F, Dickerson J. Addressing obesity in Roma communities: a community readiness approach, International Journal of Human Rights in Healthcare, 2018 https://doi.org/10.1108/IJHRH-06-2018-0038

Research Grants Held

Dickerson J, McEachan R, Pickett K. et al. Understanding the impact of covid-19 on pregnant women and new parents: The Born in Bradford 2020 Families Study. MRC (UKRI Covid Grant), £325,147.

Dickerson J, McEachan R, Pickett K. The Better Start Bradford Innovation Hub, Phase 2. 2020-2025. The Community Fund, £4,889,000

Dickerson J, Prady S, Willan K et al. Inequalities in Perinatal Mental Health identification & access to services. 2020-202. West Yorkshire Integrated Care Services, £225,000

McEachan R, Dickerson J, Pickett et al. The Born in Bradford COVID-19 Research Study: An adaptive mixed methods longitudinal study of the impact of COVID-19 on health inequalities in families living in Bradford. The Health Foundation, £174,139

Wright J & Dickerson J et al. Bradford Inequalities Research Unit 2019-2024. Bradford City CCG, £831,163

Cameron C, Bedford H, Dickerson J et al. COVID-19: Families, children aged 0-4 and pregnant women: vulnerabilities, resources and recovery in Tower Hamlets. Je-S (UKRI Covid Research grant). £302,154<br><br>Wright, J et al. ActEarly: a City Collaboratory approach to early promotion of good health and wellbeing. UK Prevention Research Partnership Consortium Award. £6.3 million.<br><br>Wright J, McEachan et al. NIHR Applied Research Collaboration: Yorkshire and Humber. 2019-2024. £9 million (Co-Investigator on Early Life and Prevention Theme)<br><br>MaCleod, J, Wright J, Dickerson J et al. Cohorts as Platforms for Mental Health research (CaP:MH) 2018-2020. Medical Research Council. £1,497,192. (Ref: MC_PC_17210).<br><br>Bowyer-Crane C, Nielsen D, Bird P, Bryant M, Dickerson J. Talking Together: An early evaluation of a home-based language support programme for families of children aged 2 years. 2018-2021. Nuffield Health. £226,991.

NIHR | National Institute for Health Research

NIHR Health Services and Delivery Research Programme

CO-APPLICANT INFORMATION

Co-applicant Information

**Name**	Dr Jessica Eccles
**Role and organisation**	Clinical Senior Lecturer
Department	Neuroscience
Organisation	Brighton and Sussex Medical School
Email	J.Eccles@bsms.ac.uk

Co-applicant Information – Qualifications

**Degree/subject professional Qualification**	**Awarding body, date of award**
PGCHE - Higher Education	University of Sussex - 02/10/2017
PhD - Neuroscience	Brighton and Sussex Medical School - 15/02/2016
Diploma - Cognitive Analytic Therapy	Sheffield Hallum University - 01/10/2014
MSc - Psychiatry	University of Brighton - 05/08/2013
Member - Psychiatry	Royal College of Psychiatrists - 15/06/2011
MA (Hons) - Cantab	University of Cambridge - 14/10/2006
MB ChB - Medicine	University of Oxford - 15/07/2006
BA (Hons) - History and Philosophy of Science	University of Cambridge - 15/07/2003

Patient/Service user or carer applicants

**Patient / service users or carer applicants information**

RECENT PUBLICATIONS AND RESEARCH GRANTS

**Commitment to this Research Project**	0.1 % FTE

Recent Relevant Publications

Eccles JA, Davies KA (2021) The challenges of chronic pain and fatigue. Clinical Medicine. In press.

Csecs J LL, Iodice V, Themelis K, Rae CN, Dowell NG, Simmons R, Brooke A, Prowse F, Themelis K, Critchley HD, Eccles JA. (2020) Increased rate of joint hypermobility in autism and related neurodevelopmental conditions is linked to dysautonomia and pain MEDRXIV/2020/194118

Eccles JA, Scott HE , Davies KA, Bond R, David AS, Harrison NA, Critchley HD (2020) Joint hypermobility and its relevance to common mental illness in adolescents: a population-based longitudinal study MEDRXIV/2020/191130

Eccles JA, Thompson B, Themelis, K, Amato ML, Stocks R, Pound A, Jones A-M, Cipinova Z, Shah-Goodwin L, Timeyin J, Thompson CR, Batty T, Harrison NA, Critchley HD, Davies KA. (2020) Beyond bones - the relevance of variants of connective tissue (hypermobility) to fibromyalgia, me/cfs and controversies surrounding diagnostic classification: an observational study. Cinical Medicine (in press)

Eccles JA, Owens AP, Harrison NA, Grahame R, Critchley HD (2016) Joint hypermobility and autonomic hyperactivity: an autonomic and functional neuroimaging study. Lancet 25;387:S40

Eccles JA, Beacher F, Gray M, Jones C, Minati L, Harrison N, Critchley H (2012) Brain structure and joint hypermobility: Relevance for the expression of psychiatric symptoms British Journal Psychiatry 200(6):508-9

Research Grants Held

05/19-08/21 Fibroduck Foundation Support for ‘Viscero-sensory processes and neural responses to inflammation: mechanisms of pain and fatigue in fibromyalgia’ (£18, 000) PI

10/18-10/21 Dysautonomia International ‘Brain Fog in Postural Tachycardia Syndrome: Multi-modal neural correlates’(USD 100,000) PI

06/18-06/21 Action for ME (match funding from BSMS) ‘Using high quality HCP (Human Connectome Project) MRI to investigate effects of mild inflammation on brain function in ME/CFS’ (£92,000) Co-applicant. (PI: Prof Neil Harrison)

05/18-12/21 MQ/Versus Arthritis Fellows Award ‘Reducing anxiety in joint hypermobility: A novel targeted proof of concept study’ (£225,000) PI

02/17-08/21 Academy of Medical Sciences ‘Applying leading-edge multimodal neuroimaging and neural connectivity tools to characterise brain mechanisms that link joint hypermobility to anxiety’(£30,000) PI

04/16-09/21 Versus Arthritis ‘Viscero-sensory processes and neural responses to inflammation: mechanisms of pain and fatigue in fibromyalgia’ (£250,000) PI

NIHR | National Institute for Health Research

NIHR Health Services and Delivery Research Programme

CO-APPLICANT INFORMATION

Co-applicant Information

**Name**	Dr Alison Thomson
**Role and organisation**	Lecturer in Public Engagement and Patient Public Involvement
Department	Centre for Neuroscience and Trauma
Organisation	Queen Mary University London
Email	a.thomson@qmul.ac.uk

Co-applicant Information – Qualifications

**Degree/subject professional Qualification**	**Awarding body, date of award**
PhD - Design	Goldsmiths - 02/09/2019
MA - Design Interactions	Royal College of Art - 01/07/2010
BSc (Hons) - Interactive Media Design	Dundee University - 02/06/2008

Patient/Service user or carer applicants

**Patient / service users or carer applicants information**

RECENT PUBLICATIONS AND RESEARCH GRANTS

**Commitment to this Research Project**	3 % FTE

**Recent Relevant Publications**
Thomson, A, Rivas, C and Giovannoni, G. (2015) MS Outpatient Future Groups. BMC Health Services Research. Vol 15: 105.<br><br>Thomson A, Giovannoni G, Marta M et al. (2016). Importance of upper limb function in advanced multiple sclerosis. Multiple Sclerosis Journal. vol. 22, 676-676.<br><br>Reyes, S., Giovannoni G and Thomson, A. (2018) Social Capital: Implications for neurology. Brain and Behaviour. vol. 9.<br><br>Thomson, A., Horne, R., Chung, C., Marta, M., Giovannoni, G., Palace, J and Dobson, R. (2019) Visibility and representation of women in multiple sclerosis research. Neurology.<br><br>Thomson A, Dobson R, Baker D, Giovannoni G. Digesting science: Developing educational activities about multiple sclerosis, prevention and treatment to increase the confidence of affected families. Mult Scler Relat Disord. 2020 Nov 13;47:102624.

**Research Grants Held**
£99000. GENZYME SANOFI. Digesting Science Phase 3<br>£39000. ROCHE. MS Decisions Web APP.<br>£95674. ROCHE. Upper limb rehab tool<br>££156,118.46. ROCHE.Hand Function Rehab tool (Phase 2)

NIHR | National Institute for Health Research

NIHR Health Services and Delivery Research Programme

CO-APPLICANT INFORMATION

Co-applicant Information

**Name**	Dr Elizabeth Ball
**Role and organisation**	Consultant Gynaecologist
Department	Obstetrics and Gynaecology
Organisation	Barts Health NHS Trust
Email	eball69@gmail.com

Co-applicant Information – Qualifications

Degree/subject professional Qualification	Awarding body, date of award
Member - MRCOG	The Royal College of Obstetricians and Gynaecologists, London - 01/09/2004
PhD - Trophoblast Invasion in Pregnancy Failure	University of Newcastle - 01/03/2004
PhD - Trophoblast invasion in pregnancy failure	Newcastle University, U.K - 01/03/2004
MD - Perception of heath in east and west Germany	Hanover Medical School - 03/03/1997
Doctorate Medicine - Lay definitions of Health-Comparison between East and West Germany	Medizinische Hochschule Hannover - 01/03/1997

Patient/Service user or carer applicants

Patient / service users or carer applicants information

RECENT PUBLICATIONS AND RESEARCH GRANTS

**Commitment to this Research Project**	5 % FTE

Recent Relevant Publications

Development and validation of Clinical Prediction Models for Surgical Success in patients with Endometriosis: a research protocol

Ball, E., Karavadra, B., Jade Kremer-Yeatman, B., Mustard, C., Lee, K.M. Bhogal,S.,

Dodds, J., Horne, A. W. Allotey, J., Rivas, C. submitted to Journal of Medical Internet Research (Oct 20)

MEMPHIS: a smartphone app using psychological approaches for women with chronic pelvic pain presenting to gynaecology clinics: a randomised feasibility trial.

Forbes G, Newton S, Cantalapiedra Calvete C, Birch J, Dodds J, Steed L, Rivas C, Khan K, Röhricht F, Taylor S, Kahan BC, Ball E.

BMJ Open. 2020 Mar 12;10(3):e030164.

mHealth: providing a mindfulness app for women with chronic pelvic pain in gynaecology outpatient clinics: qualitative data analysis of user experience and lessons learnt.

Ball E, Newton S, Rohricht F, Steed L, Birch J, Dodds J, Cantalapiedra Calvete C, Taylor S, Rivas C.

BMJ Open. 2020 Mar 12;10(3):e030711.

Recent advances in understanding and managing chronic pelvic pain in women with special consideration to endometriosis.

Ball E, Khan KS.

F1000Res. 2020 Feb 4;9:F1000 Faculty Rev-83.

Smartphone App Using Mindfulness Meditation for Women With Chronic Pelvic Pain (MEMPHIS): Protocol for a Randomized Feasibility Trial

Ball E, Newton S, Kahan BC, Forbes G, Wright N, Cantalapiedra Calvete C, Gibson HAL, Rogozinska E, Rivas C, Taylor SJC, Birch J, Dodds J.

JMIR Res Protoc. 2018 Jan 15;7(1)

MRI versus laparoscopy to diagnose the main causes of chronic pelvic pain in women: a test-accuracy study and economic evaluation Khan KS, Tryposkiadis K, Tirlapur SA, Middleton LJ, Sutton AJ, Priest L, Ball E, Balogun M, Sahdev A, Roberts T, Birch J, Daniels JP, Deeks JJ. Health Technol Assess. 2018 Jul;22(40):1-92.

Music as an aid for postoperative recovery in adults: a systematic review and meta-analysis

Hole J, Hirsch M, Ball E, Meads C.

Lancet. 2015 Oct 24;386(10004):1659-71. Erratum in: Lancet. 2015 Oct 24;386(10004):1630.

Research Grants Held

RPFP NIHR: MEMPHIS (£237K)

RPFP NIHR: CRESCENDO (£136K)

ELLY BARTS CHARITY (£5000)

NIHR | National Institute for Health Research

NIHR Health Services and Delivery Research Programme

CO-APPLICANT INFORMATION

Co-applicant Information

**Name**	Dr Bilal Nasim
**Role and organisation**	
Department	
Organisation	Institute of Education, University College London
Email	b.nasim@ucl.ac.uk

Co-applicant Information – Qualifications

**Degree/subject professional Qualification**	**Awarding body, date of award**
PhD - Economics	Imperial College London - 01/09/2014
BSc - Economics and Philosophy	University of Bristol - 01/09/2007

Patient/Service user or carer applicants

**Patient / service users or carer applicants information**

RECENT PUBLICATIONS AND RESEARCH GRANTS

**Commitment to this Research Project**	5 % FTE

**Recent Relevant Publications**
Nasim, B, Schoon, I, Sehmi, R (Forthcoming, 2021). "Inequalities in childhood and their consequences for later educational outcomes" British Education Research Journal, Special Issue
Nasim, B (2020) "Changes in the relationship between social housing tenure and child outcomes over time: Comparing the Millennium and British Cohort Studies" Journal of Children and Poverty
Gambaro, L, Moulton, V, Nasim, B (2020) "Parental wealth and children's cognitive skills, mental and physical health: evidence from the UK" Child Development

**Research Grants Held**
The Effects of Teacher Pay Reforms on Teacher Pay, Teacher Careers and Student Attainment - ESRC Research Grant ES/R00367X/1 (PI, joint w/ J. Anders, A. Bryson and H. Horvath)

NIHR | National Institute for Health Research

NIHR Health Services and Delivery Research Programme

CO-APPLICANT INFORMATION

Co-applicant Information

**Name**	Dr Vadivelu SARAVANAN
**Role and organisation**	Consultant
Department	Rheumatology
Organisation	Gateshead Health NHS Foundation Trust
Email	vadivelu.saravanan@nhs.net

Co-applicant Information – Qualifications

Degree/subject professional Qualification	Awarding body, date of award
MD - Medicine	Newcastle University - 12/02/2010
Fellow - Medicine	Royal College of Physicians, London - 25/06/2008
MBBS - Medicine	The Tamilnadu Dr MGR Medical University - 30/08/1994

Patient/Service user or carer applicants

Patient / service users or carer applicants information

RECENT PUBLICATIONS AND RESEARCH GRANTS

Commitment to this Research Project	0.01 % FTE

Recent Relevant Publications

1. Saravanan V, Pugmire S, Smith M, Kelly C. Patient-reported involvement of the eighth cranial nerve in giant cell arteritis. Clinical Rheumatology. 2019;38(12):3655-3660.

2. Hackett K, Davies K, Tarn J, Bragg R, Hargreaves B, Miyamoto S et al. Pain and depression are associated with both physical and mental fatigue independently of comorbidities and medications in primary Sjögren’s syndrome. RMD Open. 2019;5(1):e000885.

3. Bezzina O, Gallagher P, Mitchell S, Bowman S, Griffiths B, Hindmarsh V et al. Subjective and Objective Measures of Dryness Symptoms in Primary Sjögren's Syndrome: Capturing the Discrepancy. Arthritis Care & Research. 2017;69(11):1714-1723.

4. Howard Tripp N, Tarn J, Natasari A, Gillespie C, Mitchell S, Hackett K et al. Fatigue in primary Sjögren's syndrome is associated with lower levels of proinflammatory cytokines. RMD Open. 2016;2(2):e000282.

5. Kelly C, Saravanan V, Nisar M, Arthanari S, Woodhead F, Price-Forbes A et al. Rheumatoid arthritis-related interstitial lung disease: associations, prognostic factors and physiological and radiological characteristics--a large multicentre UK study. Rheumatology. 2014;53(9):1676-1682.

6. Newton J, Frith J, Powell D, Hackett K, Wilton K, Bowman S et al. Autonomic symptoms are common and are associated with overall symptom burden and disease activity in primary Sjögren's syndrome. Annals of the Rheumatic Diseases. 2012;71(12):1973-1979.

Research Grants Held

2019 - Academic Health Science Network North East and North Cumbria - £5,000 to develop an app for Patient Reported Outcome Measures in Polymyalgia Rheumatica

2018 - Polymyalgia Rheumatica and Giant Cell Arteritis (UK) patient support group - £10,000 to develop GP Education program for managing Polymyalgia Rheumatica and Giant Cell Arteritis

NIHR | National Institute for Health Research

NIHR Health Services and Delivery Research Programme

CO-APPLICANT PPI - INFORMATION

Co-applicant PPI Information

**Name**	Ms Jenny Camaradou
**Role and organisation**	
Department	
Organisation	PPI based in England
Email	jenny@rethinkfunding.co.uk

Co-applicant PPI Information – Qualifications

Degree/subject professional Qualification	Awarding body, date of award
Other - EUPATI European Patient's Academy on Therapeutic Innovation	EUPATI/IMI - 30/04/2021
MSc - Psychology (graduate basis of registration BPS)	University of Plymouth - 10/12/2010
BSc (Hons) - Law with European Study LLB	University of Exeter - 03/07/2001

Patient/Service user or carer applicants

Patient / service users or carer applicants information

RECENT PUBLICATIONS AND RESEARCH GRANTS

Commitment to this Research Project	% FTE

Recent Relevant Publications

Research Grants Held
